# Perils and opportunities in using large language models in psychological research

**DOI:** 10.1093/pnasnexus/pgae245

**Published:** 2024-07-16

**Authors:** Suhaib Abdurahman, Mohammad Atari, Farzan Karimi-Malekabadi, Mona J Xue, Jackson Trager, Peter S Park, Preni Golazizian, Ali Omrani, Morteza Dehghani

**Affiliations:** Department of Psychology, University of Southern California, Los Angeles, CA 90089, USA; Brain and Creativity Institute, University of Southern California, Los Angeles, CA 90089, USA; Department of Human Evolutionary Biology, Harvard University, Cambridge, MA 02138, USA; Department of Psychological and Brain Sciences, University of Massachusetts Amherst, Amherst, MA 01003, USA; Department of Psychology, University of Southern California, Los Angeles, CA 90089, USA; Brain and Creativity Institute, University of Southern California, Los Angeles, CA 90089, USA; Department of Human Evolutionary Biology, Harvard University, Cambridge, MA 02138, USA; Department of Psychology, University of Southern California, Los Angeles, CA 90089, USA; Brain and Creativity Institute, University of Southern California, Los Angeles, CA 90089, USA; Department of Physics, Massachusetts Institute of Technology, Cambridge, MA 02139, USA; Brain and Creativity Institute, University of Southern California, Los Angeles, CA 90089, USA; Department of Computer Science, University of Southern California, Los Angeles, CA 90089, USA; Brain and Creativity Institute, University of Southern California, Los Angeles, CA 90089, USA; Department of Computer Science, University of Southern California, Los Angeles, CA 90089, USA; Department of Psychology, University of Southern California, Los Angeles, CA 90089, USA; Brain and Creativity Institute, University of Southern California, Los Angeles, CA 90089, USA; Department of Computer Science, University of Southern California, Los Angeles, CA 90089, USA

**Keywords:** psychology, large language models, natural language processing, psychological diversity, psychological text analysis

## Abstract

The emergence of large language models (LLMs) has sparked considerable interest in their potential application in psychological research, mainly as a model of the human psyche or as a general text-analysis tool. However, the trend of using LLMs without sufficient attention to their limitations and risks, which we rhetorically refer to as “GPTology”, can be detrimental given the easy access to models such as ChatGPT. Beyond existing general guidelines, we investigate the current limitations, ethical implications, and potential of LLMs specifically for psychological research, and show their concrete impact in various empirical studies. Our results highlight the importance of recognizing global psychological diversity, cautioning against treating LLMs (especially in zero-shot settings) as universal solutions for text analysis, and developing transparent, open methods to address LLMs’ opaque nature for reliable, reproducible, and robust inference from AI-generated data. Acknowledging LLMs’ utility for task automation, such as text annotation, or to expand our understanding of human psychology, we argue for diversifying human samples and expanding psychology’s methodological toolbox to promote an inclusive, generalizable science, countering homogenization, and over-reliance on LLMs.

**Significance Statement**

Not only are large language models (LLMs) such as ChatGPT becoming increasingly embedded in people’s everyday life in many societies, they are becoming an important tool in psychological research. Here, we highlight the risks associated with the rushed application of these technologies to psychological research, a practice we call “GPTology.” We review and conduct a comprehensive analysis of both the benefits and risks associated with using LLMs in psychological research and advocate for the development of reliable applications and the use of open, interpretable models. We also quantify, and warn against, cultural biases of LLMs. A more inclusive approach is critical to ensuring reproducible, generalizable, and unbiased scientific insights, when employing LLMs to study the human mind.

## Introduction

Technological innovations have enabled social and behavioral scientists to gather diverse forms of data about human psychology, paving the way for significant breakthroughs in psychological science and neighboring fields. These advancements have played a pivotal role in expanding our understanding of psychological processes. The development of neuroimaging (e.g. fMRI), online survey platforms (e.g. Mechanical Turk), and eye-tracking technology are just a few examples that have revolutionized psychological research in the last few decades. The digital revolution and emergence of “big data” facilitated the establishment of new fields such as computational social science (1). More recently, there has been a notable paradigm shift in artificial intelligence (AI) with the emergence of large language models (LLMs): neural networks characterized by their deep layers and extensive scale, typically consisting of billions to over a hundred trillion parameters and trained on vast text datasets, enabling an unprecedented ability to understand, generate, and translate human language with remarkable subtlety and complexity. These AI models are trained on extensive collections of unlabeled text using self-supervised or semi-supervised learning methods, contributing to their remarkable language understanding and generation capacity. This new technology has been argued to possess the capacity to transform social science research (2).

Language models have advanced significantly through interdisciplinary collaboration, including contributions from psychology, which laid the foundation for modern language modeling. For example, inspired by the psychology of feedback and learning mechanisms, McClelland and Rumelhart (3) explored connectionist models demonstrating the potential of neural networks, and Rumelhart et al. (4) introduced key algorithms like backpropagation. Building upon these developments and motivated by theories in working memory and cognitive processes, Elman (5) introduced recurrent neural networks, enabling researchers to model sequential data in language models.

The availability of large textual corpora, increased computing power, and advancements in deep-learning techniques have recently contributed to the progress and refinement of language models. Notably, the Transformers architecture (6), which enables understanding of intricate relationships between different input components in a remarkably efficient and precise manner substantially improved natural language understanding and generation capabilities. Subsequently, LLMs, like ChatGPT (7), based on different versions of Generative Pre-trained Transformer (GPT; (8, 9)), have had sizable implications in various domains, powering applications such as human-sounding chatbots and language translation systems. Their success lies in their impressive language generation capabilities and public accessibility, which has permeated research in various fields from medicine (10) to politics (11).

Studying how LLMs operate offers promising opportunities to gain previously inaccessible insights into cognition as a whole, and perhaps even human cognition (12, 13). Additionally, these models’ capabilities in text analysis and generation could possibly be harnessed by researchers as an easy-to-use method for analyzing textual data, such as coding texts for mental health assessment (14). There has recently been a rapid string of psychological research output related to and facilitated by these models (13, 15–17). In particular, ChatGPT has been employed in a range of social and behavioral applications, from hate-speech classification (18) to sentiment analysis (19), often with promising results.

However, drawing parallels with the turbulent integration of previous technological innovations into psychological research suggests that the hurried or negligent implementation of LLMs in psychology could give rise to unintended consequences. For example, when fMRI techniques first appeared, some researchers began haphazardly applying these techniques, resulting in many nonsensical, but statistically significant neural correlates: a phenomenon cleverly illustrated in an fMRI of post-mortem Atlantic Salmon (20), where the fish displayed apparent significant brain activity during the experiment despite being deceased, and a study on the high likelihood of finding spurious correlations in fMRI research due to statistical misapplications (21). These two episodes now serve as a lesson for psychology students and researchers more broadly: that new technologies should be cautiously integrated into psychological research. Specifically, integrating LLMs in the psychological research pipeline, such as in substitution for human participants, necessitates critically examining its limitations. Researchers should not simply ask themselves *how* they can use a new technology; they need to also ask themselves *whether* and *why* they should do so (22).

By no means do we uniformly cast doubt on the usefulness of LLMs in psychological research. Instead, we aim to contextualize current and future opportunities that LLMs may offer psychological research and suggest possible ways to navigate their limitations. While acknowledging their potential utility to improve psychological science, we advise caution regarding the unchecked application of LLMs, at least in their current state, in psychological studies. To prevent issues like nonsensical, but statistically significant correlations, it is essential to approach LLMs with caution, keeping in mind similar challenges the field has faced in recent decades (e.g. the credibility revolution; (23)). The following section provides an overview of the downsides of the hurried use of LLMs in psychology, and how it could negatively affect psychological findings if not applied critically and cautiously.

## LLMs should not replace human participants

Many studies of state-of-the-art LLMs have concluded that their outputs are highly “human-like” (24–26). For instance, Webb et al. (26) examined the analogical reasoning abilities of ChatGPT and found that it had developed an emergent capacity for zero-shot reasoning, allowing it to solve a wide range of analogy problems without explicit training. Some have argued that if LLMs, such as ChatGPT, can indeed produce human-like responses to common measures in psychological science (e.g. judgments of actions, endorsements of values, perceptions of social issues), they might as well, at some point in the future, replace the human subject pool. For example, based on a substantive correlation between moral judgments made by humans and a language model (GPT-3.5), Dillion et al. (27) asked whether LLMs can replace human participants in psychological research. While they do acknowledge limitations of the “AI as human participants” position (which is typically supported by showing an LLM-human correlation in some psychological domain; e.g. (17, 27)), such as their issues with representing different populations, capturing variability in human responses, and oversimplifying complex judgments and behaviors, it is important to stress the ethical and epistemic risks associated with this practice (28). Similarly, Ref. (29) warns against the anthropomorphism of AI systems, noting that such tendencies can mislead us into expecting human-like performance from systems that operate on fundamentally different principles. This caution is crucial as we consider the implications of deploying LLMs in roles traditionally reserved for human participants.

First, research using LLMs to simulate human participants needs to pay more attention to the substantial cross-cultural variation in cognitive processes—including moral judgments—around the globe (see Refs (30, 31)) and develop robust ways to *mimic* them using synthetic agents. Models like GPT have been trained chiefly on WEIRD (Western, Educated, Industrialized, Rich, Democratic; (32)) people’s textual data primarily in English, perpetuating the English-centricity of psychology (33), hindering efforts to take linguistic diversity seriously. Hence, these models may struggle with accurately representing diverse populations (see a discussion of LLMs inherently struggling to represent identity groups due to their training procedures in Wang et al. (34)). For example, ChatGPT has shown gender biases favoring male perspectives and narratives (35, 36), cultural biases toward American perspectives (37) or majority populations in general (38), and political biases favoring liberal, environmental, and left-libertarian viewpoints (11, 39, 40). These biases also extend to personality, morality, and stereotypes (41–43).

Generally, these models’ outputs reflect a WEIRD psychology such that the AI-human link substantially weakens as we collect “human” data from less WEIRD populations. In other words, the high AI-human correlation does not replicate when the human sample is less WEIRD (44). This is especially concerning because the WEIRD-people problem was originally devised as an awareness-raising rhetorical device to push researchers away from heavily relying on WEIRD human research participants (e.g. undergraduate students in North America; see Ref. (45)). Substituting human participants with LLM outputs would, therefore, be a step backward. Ignoring human diversity in psychology, amplified by the convenience of online samples like MTurk, has already led psychology research to be tunnel-visioned toward an extremely thin slice of human diversity (34). GPTology will constitute a move toward an even more myopic and less generalizable discipline.

Second, LLMs seem to have a “correct answer” bias (17, 40). Specifically, LLMs fail to produce much variance in their answers to psychology survey questions: even if these questions pertain to topics—like moral judgment—for which there is no actual correct answer, and for which human answers would have diversity-of-thought. Simulating human diversity-of-thought using LLMs might generally be nontrivial. For example, simply prompting an LLM to respond to a question multiple times and then measuring the response variance, a common strategy in the social sciences (17, 44, 46), does not equate to meaningful variance that can be compared with humans. Generative language models, such as ChatGPT, compute a probability distribution over possible next words to produce an output sequence in an autoregressive manner (6, 47). Specifically, the probability of a word being predicted next is given as $P(z_{i})=\frac{e^{z_{i}/T}}{\sum_{k=1}^{n}e^{z_{k}/T}}$, where $z_{i}$ represents the logit value associated with a potential output word $w_{i}$ given an input sequence $\left\{w_{1},\ldots ,w_{i-1}\right\}$, which is, i.e. the model’s internal representation of the word after processing it (17). The “temperature” parameter (*T*) expresses whether the model chooses the most likely word according to the distribution (T→0), expressing deterministic behavior, or samples the words according to the distribution (T→1), supposedly expressing more creative nondeterministic behavior. However, the output variance when repeating the same prompt using a temperature of 1 then simply reflects the output probability over the response options and therefore how sure the model is about its response, which itself is affected by the correct answer bias observed by Park et al. (17) and others.

Conceptually, this is akin to repeatedly asking a question to the same participant instead of different participants. However, psychologists are usually interested in studying variance across participants to make inferences about behavioral patterns and the robustness of psychological phenomena (see discussion of LLM outputs resembling group averages instead of individual differences in, e.g. (27)). Thus, researchers using LLMs to study human behavior need to move beyond methods that simulate single responses—such as merely predicting group averages or simulating an individual’s response across various tasks (e.g. (24, 25, 27))—and instead develop robust methods to emulate the complexity of human samples. Furthermore, LLMs are trained on vast amounts of data, which can contain many of the items and tasks used in psychological experiments, thus leading the model to rely on its memory instead of making inferences, exacerbating the problem above. To get unbiased evaluations of human-like LLM behavior, researchers need to make sure that their tasks are not part of the model’s training data (48) or adjust the models not to affect the outcome of their experiments, such as by “unlearning” data from an LLM (49).

**Fig. 1.**
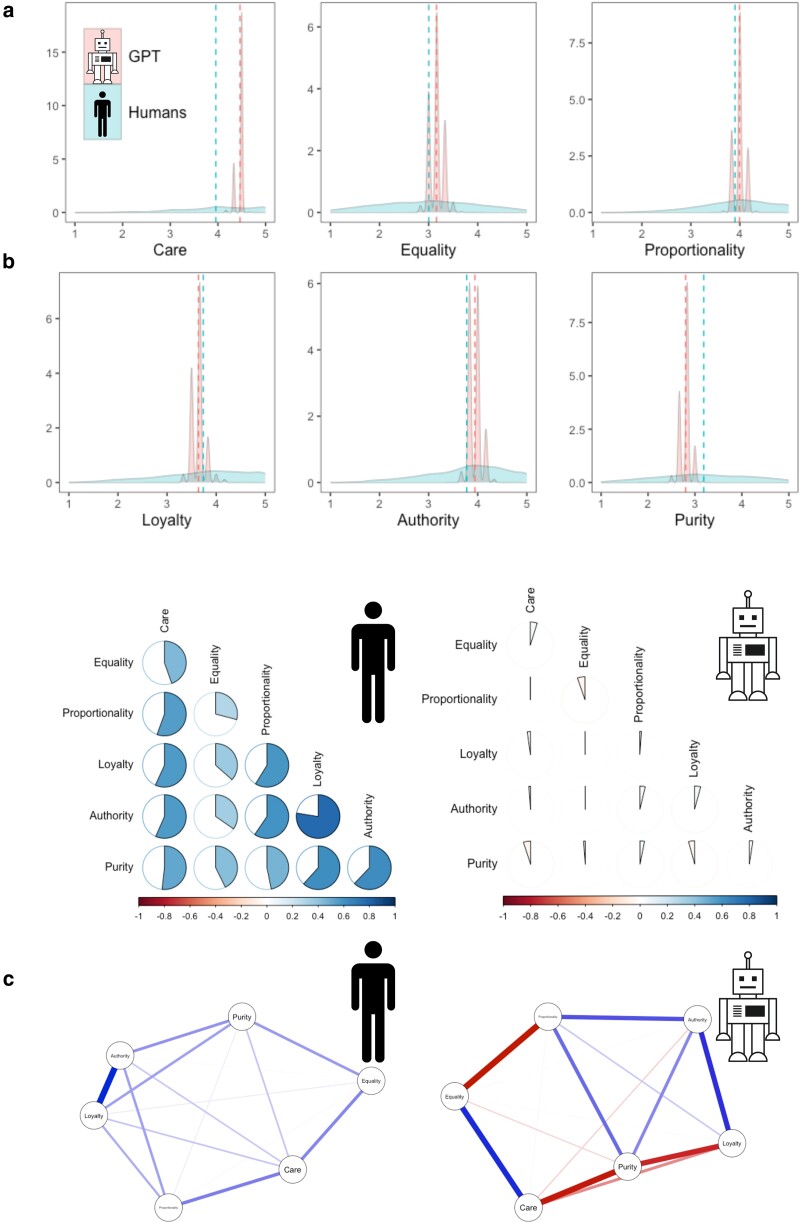
ChatGPT vs. human moral judgments. Note: a) Distributions of moral judgments of humans (light blue) and GPT (light red) in six moral domains. Dashed lines represent averages. b) Inter-correlations between moral values in humans (N=3,902) and ChatGPT queries (N=1,000). c) Network of partial correlations between moral values based on a diverse sample of humans from 19 nations (30) and 1,000 queries of GPT. Blue edges represent positive partial correlations and red edges represent negative partial correlations.

To showcase how these issues concretely manifest, we analyze LLM responses in the domain of human morality, highlighting how simple prompting strategies fail to capture human response patterns and particularly human variance. We designed a prompt and gave the Moral Foundations Questionnaire-2 (MFQ-2; (30)) to GPT-3.5 1,000 times. The MFQ-2 is a new measure published in 2023, and its items are unlikely to exist verbatim in the corpora on which GPT was trained; hence, it does not have the problem of extensively appearing in the training corpora. We then compared GPT’s responses to the distributions of a culturally diverse sample of humans from 19 populations. As can be seen in Fig. 1(a), GPT produces substantially smaller variance in six moral domains (i.e. care, equality, proportionality, loyalty, authority, and purity) compared with actual human populations (all Ps<0.001). GPT-3.5’s variance was 43–121 times smaller than human data in different moral domains, even when using parameter settings for maximum variability in generated responses. Our results, in line with recent evaluations of strategies to induce demographic variance in LLM outputs (40, 50, 51) and other emerging works (17, 52), indicate that arguments such as that “silicon sampling allows researchers to simulate a diverse population of participants,” (27) could be premature and that the so-called “silicon sampling,” at least currently, fails in mimicking a diverse population of humans: a finding which is consistent with that of Atari et al. (44).

**Fig. 2.**
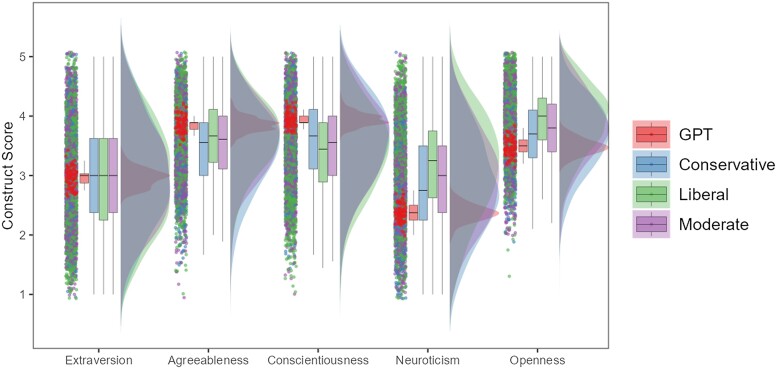
Comparing ChatGPT against humans grouped by political opinion for responses on the Big Five Inventory. Note: Figure shows the response distribution of humans and ChatGPT across the five-factor personality constructs and for different human demographics. Figure shows that ChatGPT gives significantly higher responses on Agreeableness, Conscientiousness and significantly lower responses on Openness and Neuroticism. Importantly, ChatGPT shows significantly less variance compared with all demographic groups on all personality dimensions.

**Fig. 3.**
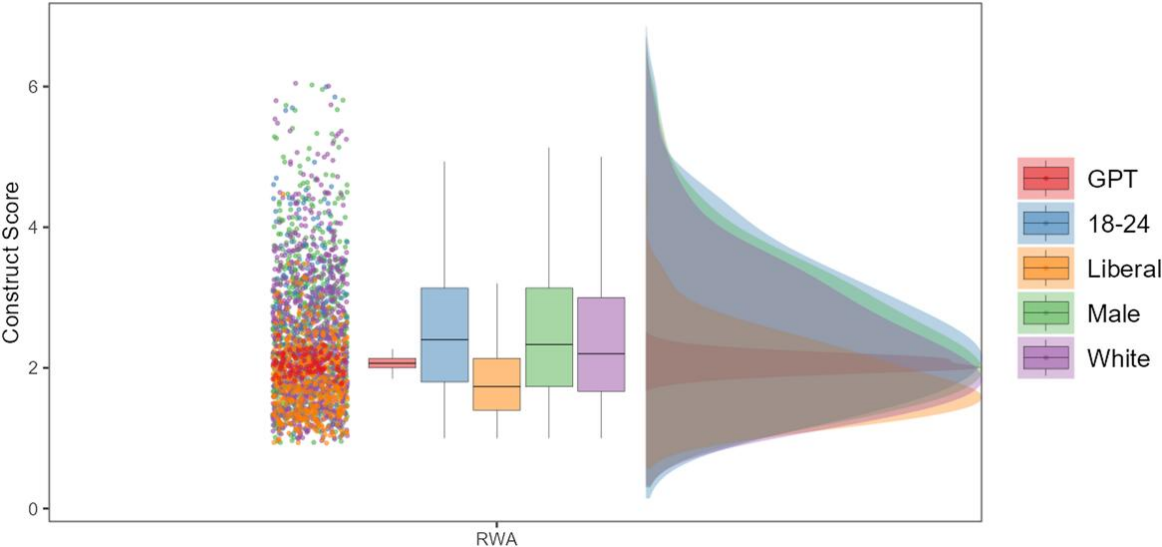
Comparing ChatGPT against humans across various demographic variables for the Right-Wing-Authoritarianism scale. Note: Figure shows the response distribution of humans and ChatGPT on the RWA scale for different human demographics. ChatGPT shows significantly lower average RWA than male, white, and young participants but not explicitly liberal participants. Importantly, ChatGPT shows significantly less variance compared with all demographic groups.

To show that this phenomenon is robust across domains, we extended this analysis of GPT vs. human responses to a broad range of self-report measures, with participants from over 43 countries and spanning various psychological domains, such as personality, cognition, political orientation, and emotions: Big Five Inventory (53): N=3,924, Need for Closure (54): N=315, Need for Cognition (55): N=900, Right-wing authoritarianism Scale (56): N=1,020, Emphasizing–Systemizing Scale (57): N=3,141, Rational–Experiential Inventory Scale (58): N=1,456. Across these psychological constructs, we consistently found that ChatGPT responses generally showed significantly less variance across all measures (see Table S20 for an overview of variance differences across all surveys) and differed significantly between various demographics. For example, when responding to personality surveys, ChatGPT was significantly more agreeable than politically liberal individuals (d=−0.230, P<0.001, 95% CI [−0.369, −0.091]), conservatives (d=−0.403, P<0.001, 95% CI [−0.550, −0.257]), and moderates (d=−0.285, P<0.001, 95% CI [−0.424, −0.145]) as shown in Fig. 2. Beyond personality dimensions, ChatGPT, for example, also endorsed significantly less right-wing authoritarianism than male participants (d=0.44, P<.001, 95% CI [0.24, 0.65]), White participants (d=0.35, P<0.001, 95% CI [0.16, 0.54]), and younger participants (18–24; d=0.49, P<0.001, 95% CI [0.24, 0.74]), but significantly more than explicitly liberal participants (d=−0.23, P=0.003, 95% CI [−0.39, −0.07]) as shown in Fig. 3. Differences of this kind and strength were observed across all surveys and demographic groups. See the Supplementary Materials for a detailed summary of all demographic differences, across a variety of psychological constructs.

Third, we are skeptical that GPT responses can make nomological networks in well-established theoretical frameworks. Dillion et al. (27) mention that “researchers can give LLMs different questions and see if they act as expected within a nomological net (e.g. form a reliable scale).” To demonstrate how GPT constructs a moral psychological nomological network, we looked at inter-correlations between moral domains (Fig. 1(b)) and the network of moral domains based on partial correlations (Fig. 1(c)) in a diverse human sample (*N* = 3,902; (30)) and 1,000 GPT queries. As shown in Fig. 1(b) and (c), the networks of moral values are substantially different, indicating that GPT struggles to produce previously established nomological networks.

In sum, probing LLMs can be a fruitful direction for future research (2, 59). However, we caution against the replacement of human participants with LLMs, because (i) synthetic AI-simulated sampling would ignore psychological and linguistic diversity that the field desperately needs in order to investigate beyond WEIRD psychology (e.g. (32–34, 60)); (ii) LLMs often fail to show meaningful variance (or diversity) in their judgments; (iii) the outputs of current LLMs do not seem to replicate previously established nomological networks; and (iv) reliance on LLMs may foster epistemic complacency and illusions of understanding, potentially resulting in scientific monocultures that suppress diverse methods, curtail innovation, and increase vulnerability to errors (28).

Overall, probing the psychology of LLMs is scientifically meaningful and practically important. However, it should not replace the scientific study of *Homo sapiens* but rather supplement it (e.g. (27, 28)). Of course, these limitations do not imply that all uses of LLMs should be avoided in psychological research. For example, some researchers have argued that AI could be regarded as an agent of “assistance,” “improvement,” or “augmentation” (e.g. (22)). Framed in the latter way, LLMs can be thought of as tools to help improve the research process in psychology. It is indeed exciting to try different psychological measures (traditionally developed for humans) on AI tools, but such examinations should be cautious and exploratory, and their results should not be hyperbolized. Similarly, LLMs have the potential to serve in a role analogous to that of agent-based models. For example, some recent studies present proofs-of-concept of how AI systems could simulate human behaviors (e.g. (61–63)). Therefore, LLMs show promise to become powerful simulators in psychological research, offering novel possibilities for enhancing our understanding through accessible and potentially scalable simulations, particularly when traditional participant-based methods are impractical. However, unlike traditional agent-based models, which define the environment, agents, interactions, and their consequences to allow for inferences based on these parameters, LLMs largely operate as black-boxes. Combined with issues of the outputs being often misaligned from real human behaviors and responses, as discussed above, this remains a serious concern: a collection of nebulous agents that can give misaligned/misleading responses. Thus, more work, particularly around validating LLM simulations, is required to establish LLMs as a viable alternative to or extension of traditional agent-based models. Such careful considerations may lead to the development of more robust procedures to implement LLMs in psychology, similar to how psychologists have addressed issues with other technologies. When researchers decide to use LLM outputs as a proxy for a human sample, they should, for example, take into account task-relevant biases of the models in question (e.g. check for implicit biases against agents involved in a moral scenario when investigating moral judgments). This can be done in conjunction with efforts to reduce LLM biases (64).

## LLMs are not a panacea for text analysis

In this section, we discuss the perception of LLMs as a quasi-universal off-the-shelf text analysis tool in psychology. We begin by explaining key distinctions in how LLMs can be applied for text analysis. This is followed by an exploration of the currently common practice of overly focusing on zero-shot capabilities of LLMs and an examination of their potential drawbacks. We then contrast LLMs with non-LLM-based, top-down, theory-based text analysis tools, discussing the advantages of these smaller, interpretable methods and how they can benefit researchers. We highlight that researchers must carefully consider the particular demands and subtleties of their study topics when selecting natural language processing (NLP) methods for text analysis in psychology. This might include leveraging LLM-based approaches in scenarios where large-scale, general-purpose language modeling is necessary. Conversely, in situations requiring theory-driven explorations, top-down methods such as thematic content analysis or dictionary-based techniques (e.g. LIWC) may be more suitable. By thoughtfully combining these methods based on the study’s needs, researchers can achieve a more nuanced and comprehensive understanding of their textual data.

NLP methods using pretrained language models generally fall into two categories: those involving parameter updates (e.g. gradient-based) and those that do not. Fine-tuning is a primary method involving parameter updates, where the pretrained LLM is further trained on a task-specific dataset. This can range from updating a subset of parameters to adjusting the entire model, depending on task complexity and data availability (65–68) In contrast, zero-shot, one-shot, and few-shot learning, can be used without gradient updates by leveraging a pretrained model’s ability to generalize from limited or no task-specific data (i.e. examples), using the model’s pre-existing knowledge and understanding (8, 69, 70).

Recently, there has been a surge of studies using LLMs zero-shot capabilities, that is their capability to perform tasks without any prior training on that specific task (8, 71–73), for psychological text analysis, presumably due to their ease of use and accessibility. For example, Markowitz (74), Rathje et al. (75) and Zhu et al. (19), reported high performance of ChatGPT as an automated text analysis tool, such as for sentiment analysis, offensive language, thinking style, or emotion detection. Rathje et al. (75) further concluded that LLMs constitute a viable all-purpose method for psychological text analysis, arguably more convenient than small(er) language models and traditional techniques in NLP, due to their ability to handle diverse tasks within a single model without needing task-specific adjustments, and their user-friendly design that minimizes the need for complex coding, making them more accessible to psychologists and potentially encouraging broader research engagement. However, this perspective of LLMs, as a convenient and comprehensive tool in psychological text analysis, is challenged by recent critiques emphasizing their limitations, such as inconsistencies in text annotations, difficulties in explaining complex constructs such as implicit hate speech, and a potential lack of depth in specialized or sensitive areas (18, 76, 77).

Furthermore, Reiss (77) demonstrated that LLMs may produce inconsistent outputs when subjected to minor prompt variations and can fall short of scientific thresholds for reliability, sometimes even when pooling outputs from multiple repetitions and prompts. Kocoń et al. (76) found that LLMs may struggle with complex, subjective tasks such as emotion recognition, and Huang et al. (18) found that LLMs can reinforce lay perspectives when detecting implicit hate speech and may thus mislead nonexperts with their explanations in cases where they make incorrect decisions. Furthermore, the difference in convenience between zero-shot applications of LLMs and fine-tuning of models may not be as stark as commonly perceived. Fine-tuned small(er) language models for various tasks are now increasingly publicly available. Similarly, there are more and more high-quality and specialized datasets available to researchers for fine-tuning language models themselves. Examples of such corpora span across fields, such as morality, personality, or sentiment analysis (78–83).

Notably, even smaller fine-tuned models can in many cases, where data for fine-tuning is available, fare equally well or even better than zero-shot applications of LLMs. For instance, in most reported cases in Rathje et al. (75), small fine-tuned language models, including older models such as base-BERT (Bidirectional Encoder Representations from Transformers; (65)), outperform even the newest generation of ChatGPT (GPT-4) on a variety of text annotation tasks. Notably, in Rathje et al. (75) GPT-4 (zero-shot) was outperformed on English language sentiment analysis by a fine-tuned model developed before the breakthrough Transformers architecture (84). Additionally, these smaller language models have since then further undergone significant improvement, such as Sentence-BERT (85), RoBERTa (86), and their subsequent iterations, highlighting additional potential performance advantages. While zero-shot applications of LLMs might offer immediate accessibility, the most expedient choice is often not the most effective. Thus, researchers should be cautious of leaning too heavily on the allure of convenience. We explicitly highlight here that appropriate methods do not exclude LLMs. There are various alternative ways to utilize LLMs, such as by fine-tuning the LLM on the respective task, or via few-shot prompting which includes few examples of solving a task in a prompt so that the LLM can generalize to the given examples (8, 72, 87).

Building on this perspective, we extend these recent studies by investigating ChatGPT’s ability to annotate moral language across three distinct settings: zero-shot, few-shot, and fine-tuned. Extracting moral values from (online) texts is a difficult task, where even trained human annotators show significant variance. This is because moral values are often only implicitly signaled in language, and online posts often contain little contextual information due to length constraints. For example, past works (e.g. (83, 88)) achieved average F1 scores of 0.50 and below despite training and evaluating their classifier for each moral value. Even when adding considerable background knowledge about posts’ content, such as in Lin et al. (89), reported F1 score remain moderate (between 0.54 and 0.76). Human morality has also been argued to be a particularly difficult concept to grasp for language models, making it an interesting test case for comparing powerful LLMs against less complex smaller language models (90), as well as testing the efficacy of different LLM application strategies. Specifically, we gave ChatGPT 2,983 social media posts that contained moral or nonmoral language and prompted it to determine if any and what specific type of moral language was used. We then compared it to a small BERT model that was fine-tuned on a separate subset of social media posts (see Supplementary Materials for details). To supplement our comparison of smaller vs. larger and complex vs. less complex models, we repeated this analysis using the Linguistic Inquiry and Word Count (LIWC; (91)), which is a commonly used dictionary-based text-analysis method producing psychologically validated, and importantly interpretable features, from a given text. We compared the annotations of all models against the “ground truth” of human raters.

We find that fine-tuned BERT vastly outperformed ChatGPT applied in a zero-shot setting, achieving an F1 score of 0.48 vs. ChatGPT’s 0.22. Additionally, ChatGPT was more extreme in over- or under-predicting a moral sentiment, while BERT did in all but one case not significantly deviate from trained human annotators. Notably, even the LIWC-based approach outperformed ChatGPT (zero-shot) with an F1 score of 0.27 and was significantly less likely and less extreme in deviating from trained human annotators, despite being a magnitudes smaller, less complex, and cheaper model. Notably, ChatGPT (zero-shot) showed significantly more false positives, being over 10 times more likely Δodds=+1,240%,P<0.001 to predict a text as moral compared to human raters (see Table S2). As anticipated, both few-shot learning and fine-tuning increased the performance of ChatGPT in our experiments. When applied in a few-shot setting, ChatGPT achieved an F1 score of 0.32. Notably, fine-tuning ChatGPT on the same data used for the BERT model resulted in a superior F1 score of 0.53 outperforming the fine-tuned BERT model. Particularly, we find that fine-tuning ChatGPT leads to significant improvement in identifying the Loyalty foundation (about 25% increase in F1 score) and Care foundation (about 10% increase in F1 score). See the full overview of foundation-level F1 scores for all classifiers in Table S1. However, note that in our experiments, ChatGPT was still more extreme in over- or under-predicting individual moral sentiments compared with BERT, which replicated the distribution of moral foundations most similar to human annotators. For example, ChatGPT (few-shot) was about twice as likely Δodds=+90%,P<0.001 more likely to predict a text as nonmoral compared with human raters. See Table S2 for an overview of the predicted foundation distributions compared with human ground truth.

Additionally, the performance of both zero-shot and few-shot ChatGPT was extremely poor on several foundations, often reaching F1 scores of nearly zero. Rathje et al. (75) report similarly low performances for the current flagship versions, GPT-4 and GPT-4-Turbo, on the Authority, Purity, and Proportionality foundations. Our fine-tuned models also performed substantially better on average, with fine-tuned BERT achieving an average F1 score of 0.48 and fine-tuned ChatGPT-3.5 an average score of 0.53, compared with reported F1 scores of 0.34 for GPT-4 and 0.29 for GPT-4-Turbo. In fact, our fine-tuned BERT model demonstrated superior performance over both GPT-4 and GPT-4-Turbo across all foundations except the Loyalty foundation, and fine-tuned GPT-3.5 outperformed GPT-4 and GPT-4-Turbo (zero-shot) on all foundations. See Table S3 comparing our fine-tuned model to the GPT-4 results presented by Rathje et al. (75) on the same moral foundations. Interestingly, even our zero-shot application of GPT-3.5 outperformed the reported GPT-4 results on three foundations (Care, Proportionality, Authority), indicating inconsistent advancements between newer and older models. Further comparisons (Tables S7 and S8 in Rathje et al. (75)) on another moral foundations dataset, on which the BERT model was not fine-tuned (MFTC; (81)), showed that BERT, despite being applied cross-context, outperformed GPT-4 on the Authority and Purity foundations, and GPT-4-Turbo also on the Loyalty foundation. GPT-4 performed slightly better than BERT on the Loyalty foundation and significantly better only on the Care foundation. Rathje et al. (75) argue that the change in performance for the fine-tuned BERT models between datasets is indicative of fine-tuned models’ inflexibility across different contexts or datasets. We suggest a slightly different narrative, given that the fine-tuned model still outperformed GPT-4-Turbo on three out of four foundations (and two out of four for GPT-4) and performed better on average than both. These findings show that, first, LLMs’ supposed cross-context and flexibility advantages may not always hold, and second, that using LLMs conveniently “out-of-the-box” can sometimes drastically fail while highlighting that fine-tuning can mitigate the very same issues.

It should be noted here that while fine-tuning ChatGPT or applying a few-shot paradigm can yield better results, such processes are significantly more resource-intensive and expensive compared with LIWC or even BERT. See also the discussion of environmental costs of LLMs in the Supplementary Materials, as training and deploying LLMs is energy intensive and therefore connected to significant carbon emissions (92, 93). Overall, our results show that LLMs can be a superior tool but they also show that achieving this level of performance might not always be trivial and convenient. For example, fine-tuning can be costly, might not always work, and negates many of the conveniences of using LLMs in a zero-shot setting by requiring a dataset for training and expertise with training models. As such, researchers should weigh the benefits and drawbacks of each method before choosing one, similar to how they would choose a statistical method for their data analysis.

Additionally, ChatGPT and other closed-source models require (if possible at all) fine-tuning on a corporate platform, which introduces constraints compared with models like BERT which provide full control about the fine-tuning process and can be trained offline without additional costs (see the following section for a detailed discussion of issues with closed-source and proprietary vs. open-source models). Few-shot learning, although promising, hinges on the relevance and quality of examples, potentially making it less stable than fine-tuning (94). As such, researchers should choose a model and how to apply it based on the availability of task-specific data, available computing resources, and research-specific considerations (e.g. interpretability, control over the model). Fine-tuning is preferable if training data are available. Smaller models (e.g. BERT) are then preferable to larger models (e.g. ChatGPT) if computing resources (or financial, for commercial services) are limited. In scenarios where gathering extensive task-specific data is impractical or impossible, LLM’s zero-shot or few-shot capabilities are advantageous.

To further highlight the limitations of zero-shot LLM applications, we examined to what extent ChatGPT’s zero-shot annotations were biased toward specific demographics. We did so by using human annotator demographics and psychometrics provided for our test data (see Supplementary Materials for a detailed description of the experimental procedures). We found that ChatGPT is less likely to agree with conservative (Δodds=−4%, P<.001) or collectivist (Δodds=−30%, P<0.001) annotators and more likely to agree with individualistic (Δodds=+142%, P<0.001) annotators. Additionally, it is biased toward younger ($\Delta odds=\frac{-1\%}{year}$, P=0.033), more open-minded (Δodds=+1244%, P<0.001), and agreeable (Δodds=+156%, P<0.001) annotators. Interestingly in terms of the six moral foundations (30), it is biased toward annotators who have a lower preference for equality (Δodds=−97%, P<0.001) and loyalty (Δodds=−96%, P<0.001) and those who endorse more care (Δodds=+31,159%, P<0.001) and proportionality (Δodds=+21,215%, P<0.001) values. This aligns with our additional findings of ChatGPT over-weighing the care foundation when responding to the MFQ-2 (see additional analyses in Supplementary Materials). Potentially, this could be linked to post-hoc measures by OpenAI to avoid an AI that endorses harm or does not care about people’s emotional well-being. Agreement with proportionality and equality endorsing annotators aligns with past findings that ChatGPT is (left-)libertarian-leaning (11, 39, 40). See Tables S4–S10 for a summary of annotation biases.

Finally, we analyzed how LLMs, at least at this stage of their development, fare against theory-based, top-down constrained methods. This is relevant because LLMs are primarily considered language analysis tools due to their broad capabilities stemming from their vast parameter sets, training data, and training procedures. However, this flexibility and performance come at the price of reduced interpretability and reproducibility (i.e. the trade-off between a higher-performing black box and a lower-performing interpretable method). An often-stated reason for why some researchers of psychological text analysis prefer neural-network-based models over simple, theory-driven methods is the purportedly superior predictive power of the former. If LLMs cannot outperform top-down methods in zero or few-shot settings, this would be another reason for psychologists (and other social scientists) to consider using top-down models rather than LLMs (at least the existing ones), because in social sciences, theory and interpretability are of prime concern (95).

We compared ChatGPT against Contextualized Construct Representation (CCR; (52)), which is a method that combines psychometric scales with small language models (e.g. S-BERT; (85)) to extract psychological information from texts. This includes assessments of individuals’ values, political ideology, cultural norms, religiosity, and need for cognition. By relying on validated psychometric scales, CCR places strong, theory-based, top-down constraints on its underlying small language model, which provides better interpretability and easy application. Comparing ChatGPT and CCR, we found that ChatGPT (zero-shot) fails to outperform CCR in inferring psychological outcomes from human-written essays. CCR substantially outperformed ChatGPT ratings when prompted to infer psychological constructs directly (Dunnett’s Test; d=−2.25, P=0.005, 95% CI [−3.88, −0.62]), that is predicting the construct scores directly. See Figs. S30 and S31 for an overview of model performances across all self-report measures (i.e. cultural orientation, personal values, moral judgments, political ideology, need for cognition, and norm violations). CCR and ChatGPT performed on par when inferring psychological variables from essays about everyday life (Fig. S31) when prompted on the item-level, that is, when predicting each scale item separately and then calculating the construct score (Dunnett’s Test; d=0.21, P=0.9404, 95% CI [−1.42, 1.84]).

Taken together, past work and our present findings consistently demonstrate that for many use cases, smaller (fine-tuned) models can be more powerful and less biased than the current large (generative) language models, particularly in zero-shot and few-shot settings. For example, consider a study examining the language used in online support fora for individuals with anxiety disorders. A researcher using a smaller, specialized language model may be able to uncover subtle nuances and specific patterns of language that are directly relevant to the domain of interest (e.g. worry, intolerance of uncertainty). This targeted approach can yield more profound insights into the experiences of individuals with anxiety, shedding light on their unique challenges and potential interventions. By leveraging specialized language models or top-down methods such as CCR, or LIWC, researchers can strike a balance between comprehensiveness and granularity, enabling a more nuanced exploration of textual data.

Crucially, we do not discourage the use of LLMs as a text-analysis tool in all respects. Particularly in cases where data for fine-tuning is scarce, such as with new constructs or understudied populations, LLMs’ zero-shot capabilities may still offer acceptable performances and allow researchers to investigate urgent research questions. Methods such as few-shot prompting can be effective and efficient in these cases as they only demand a handful of representative examples. Across our experiments, we found that LLMs can achieve high performances but stress that this cannot always be achieved using the model’s zero-shot capabilities and instead requires exploring techniques, such as few-shot prompting or outright fine-tuning of the LLM (see also work on optimizing prompt design, such as “Chain-of-Thought” (71, 96), a recent technique for eliciting complex multistep reasoning). Similarly, our additional analyses, showing increased performance for ChatGPT when predicting at the item level instead of the construct level, highlight that LLMs can benefit from integrating theory-driven approaches. Extending this line of work, developing methods that can combine the benefits of both approaches is a promising endeavor for future work. With the constant and rapid development of LLMs that address performance and bias issues, these concerns could already be mitigated in the near future.

We emphasize the importance of researchers constantly assessing these limitations and opportunities while exercising caution when defaulting to the most convenient choice. As with all empirical methods, LLM-based methods need to be validated and benchmarked. We recommend benchmarking LLM-based findings against more established text-analytic methods to make LLMs more useful for psychological inference. For example, the gold-standard measure for text annotation is human annotation, and LLM-based annotations should be comprehensively validated—on a task-by-task basis—against a small labeled corpus before using these models at scale (97). This approach helps establish the validity of the analysis by reducing the potential biases that may arise from relying solely on automated techniques. Additionally, validation allows researchers to assess the inter-rater reliability of the annotations, providing a measure of the robustness of the analysis. The human element in validation brings valuable insights, subjective judgments, and contextual understanding that may be challenging for LLMs to capture accurately.

## Reproducibility matters

Reproducibility pertains to replicating and verifying results using the same data and methods (98). However, particular challenges arise when applying these principles to LLMs, particularly proprietary ones, whose black-box nature impedes the reproducibility of findings that pertain to them. This limitation poses a significant obstacle to achieving reproducibility in studies that rely on LLM-generated data or analyses beyond the often discussed nondeterministic nature of LLMs (see discussions of nondeterministic outputs and “temperature” parameter of LLMs in Refs. (46, 75, 99)) and LLMs’ black-box nature impeding interpretability (e.g. (99)). Importantly, these issues can interact to cause further obstacles for the application in psychological research.

For example, LLM biases can change over time for LLMs that undergo updates over time. Each time modifications are made to ChatGPT’s algorithms for performance enhancement, the nature and scope of biases embedded in the model may change. This could impact the effectiveness of previously established “best practices” and debiasing strategies (15, 100). Currently, ChatGPT, and to our best knowledge any other closed-source model, does not freely provide past versions that allow researchers to use the model from specific points in time (e.g. “gpt3.5-January-2023”) to reproduce research results. After each major update, it only provides a snapshot, which is then deprecated within 3 months to 1 year. This means that even when updates address and mitigate detected biases, they also introduce the potential for “process reproducibility failure” (98) in the generated data and impede reproducibility, critical for scientific rigor (15, 100, 101). This also affects currently emerging practices for reproducibility, such as researchers sharing prompts and Application Programming Interface (API) parameters because the effect of these parameters (or the parameters themselves) and the outputs generated from these prompts can change over time. Importantly, new iterations do not guarantee equal or better performance across all tasks. For example, Rathje et al. (75) report inconsistent results between GPT-3.5 and GPT-4 performance on various text-analysis tasks—for example, GPT-4 sometimes performs more poorly than GPT-3.5—which lends support to our concern that nontransparent changes to the model can cause unforeseen challenges. Additionally, current state-of-the-art LLMs come with additional opaque fine-tuning, such as reinforcement learning using human feedback (RLHF; (102, 103)), for which usually neither the training method nor the training data are known. Therefore, researchers should carefully consider the trade-off between forgoing control over these procedures (100) and fine-tuning models themselves.

Researchers should be aware of the current black-box nature of LLMs (15, 100) not only from an open-science standpoint. More generally, researchers should be interested in access to high-quality, informative semantic representations and the algorithms used to generate outputs, instead of only the outputs. One of the main advantages of computational models is that they allow us to “look under the hood” and thus make inferences about psychological processes that may be difficult to test otherwise. Thus, using proprietary LLMs that do not provide this level of access may constitute a missed opportunity for theory-based work in psychology that aims to leverage innovations in computer science. Notably, the lack of transparency in how ChatGPT generates responses implies that researchers cannot ascertain the underlying mechanisms and origin of biases that may be influencing the outputs (101, 104, 105). Such lack of transparency is antithetical to the scientific principles of openness and replicability that should be central to computational–psychological research (106).

Computational–psychological research that aims to achieve replicable findings with LLMs can use models that have a publicly available open architecture, such as BigScience Large Open-science Open-access Multilingual Language Model (BLOOM; (107)) and Large Language Model Meta AI (LLaMA; (108)). These open-source models provide researchers with access to network architecture, including datasets and parameters such as pretrained weights (109). The less black-box nature of these open-source models can help researchers make the exact version used in their works available to others (100), thus facilitating transparency and reproducibility. In the Supplementary Materials, we detail how a complete research pipeline using a LLaMA-based model can be conducted and show that the results are comparable to a pipeline using a closed-source LLM. Despite the model’s significantly smaller size (7 billion vs. 175 billion parameters) and while being self-hosted on consumer-grade hardware, it achieved a comparable performance in a text annotation task discussed above (F1 of 0.23 vs. 0.22) and a similar response pattern when answering self-report psychological measures. Compared with closed-source LLMs, open-source LLMs may thus come with a replicability benefit and increased control over the model when it comes to computational–psychological research. Additionally, certain approaches aimed to allow for more robust inferences regarding LLM outputs, such as “unlearning” (49) data and tasks used for evaluating LLM capabilities from the training data, require an open model architecture (e.g. the ability to access and manipulate the probability distribution over the output tokens).

However, the primary focus should not be a debate of closed-source vs. open-source LLMs, but instead addressing the specific problems for scientific research using either approach. Closed-source models, such as ChatGPT, may introduce ways to save and reload or share model weights and modify their models. For example, OpenAI continuously adds (but also deprecates) available model parameters. As of writing, increased reproducibility has been announced as in beta development, and a “seed” parameter increasing deterministic behavior as well as a “system_fingerprint” value to help track model changes was recently added. However, the “seed” does not guarantee reproducibility, and while the “system_fingerprint” increases transparency, allowing researchers to identify potential reasons for failure to replicate, it does not increase reproducibility itself. Moreover, some problems that might be more pronounced with proprietary models, such as economic motivations, can still affect open-source models (e.g. LLaMA being developed by Meta, a for-profit company). Additionally, openness is a spectrum requiring researchers to closely monitor to what extent LLMs fulfill relevant openness criteria for their projects. See Liesenfeld et al. (110) discussing of LLM openness in detail as well as their openness ratings of current LLMs https://opening-up-chatgpt.github.io, including their specific openness criteria and evaluation strategies https://github.com/opening-up-chatgpt/opening-up-chatgpt.github.io/tree/main/projects#criteria. As such, researchers should consider to what extent each model and the organization behind it conflicts with scientific and ethical principles of research (111–113). We caution against mistakenly treating the use of open-source rather than closed-source LLMs as sufficient to solve the black-box problem: a problem that remains largely unsolved, even for open-source LLMs. The overconfidence with which researchers may trust open-source LLMs’ findings as replicable and transparent can systematically cause such researchers to over-rely on these findings, even in out-of-distribution settings where the findings may not generalize.

The reproducibility concerns surrounding computational–psychological research done via LLMs are further intensified by their ability to adopt different perspectives through *prompting*: explicit instructions to the model on which the output is conditioned on (42, 114, 115). Prompting has been shown to be a promising technique to increase the range of applications and versatility of LLMs (116) and is also discussed as a strategy to mitigate some of the biases in these models’ outputs (117–119). One idea is to condition LLMs’ outputs on explicit instructions to take the perspective of different groups or demographics. For example, some work has shown that prompting can improve the alignment of group-relevant responses (e.g. if prompted to respond like a Democrat, ChatGPT will answer opinion poll questions in the direction steered). However, at the moment, prompting nonetheless falls short in closing the gap in demographic group biases (40) with some studies observing overly simplistic cultural (outgroup) stereotypes instead of perspective-taking (34, 51). Wang et al. (34) argued that due to their training processes, LLMs inherently struggle with accurately representing identity groups, often leading to distorted, simplified, and stereotypical portrayals of these groups. Consequently, relying solely on prompting strategies might be insufficient to resolve these issues. Moreover, prompting also introduces significant challenges to reproducibility in psychological research, because prompts can be constructed in numerous ways. Past work has shown that slight alterations and modifications in phrasing, context, or order can lead to substantially different responses (17, 74, 120–123).

We extended this literature by directly testing if changes in prompts would affect the results of our previously presented experiments. We repeated the moral sentiment analysis with a modified prompt (see Supplementary Materials) and tested whether this led to different classification outputs. Furthermore, we repeated the survey responses collection using several modified prompts (see Supplementary Materials) and tested whether the responses changed. The changes to the prompts were derived from past work that showed how minor changes to the self-report design, such as adding contextual information or changing the response scale, can elicit different response patterns (124). Our findings demonstrate, in line with recent works on the effect of prompting (17, 74, 120–122), that minor changes in prompts lead to significant differences in outputs. For example, adding a study introduction when prompting ChatGPT to respond to the Big Five Inventory (BFI; (53)), significantly reduced the scores for Openness (d=−0.30; P<0.001; 95% CI [−0.34, −0.26]), Extraversion (d=−0.54; P<0.001; 95% CI [−0.58, −0.50]), and Agreeableness (d=−0.14; P<0.001; 95% CI [−0.18, −0.10]). Similarly, modifying the order in which the types of moral foundations are defined in the prompt changed how texts were classified. Using the modified prompt, ChatGPT was significantly less likely to annotate the care foundation (−56%, P<0.001), the equality foundation (−62%, P<0.001), the loyalty foundation (−33%, P=0.009), and significantly more likely to annotate the authority foundation (+71%, P<0.001), and purity foundation (+164%, P=0.020). See the Supplementary Materials for extended results and a detailed overview of our procedures.

Our research emphasizes the importance of considering context and specific prompts when using ChatGPT to emulate diverse human behaviors (125). Continuous algorithmic changes necessitate ongoing validation of prompting strategies, a task made challenging by the complex and opaque nature of LLMs (72, 123, 126). While these challenges do not negate the applicability of LLMs in psychological research, they do highlight the need for robust, standardized methods akin to those used in traditional human research (124, 127). As this is a rapidly evolving field, new AI models may account for all of the above concerns of diversity, variation, transparency, accuracy, and robustness. The progress that is expected to be made by LLMs in the near future requires that empirical work be completed to highlight their strengths, weaknesses, and promises for further enhancement (74).

Lastly, there is a point to be made about the democratization and accessibility of computational tools. The newest generation of LLMs offered as an online service (e.g. ChatGPT, Gemini, Claude) provide many researchers with an accessible yet powerful tool—representing a pivotal advancement. However, with this greater accessibility comes the responsibility to stay educated on both the capabilities and limitations of these models. Researchers need to maintain a critical perspective, *especially* given the impressive performance and interactive nature of LLMs on some tasks, which might lead to a presumption that they are *always* the best option as the subject of study or as an assistant for automated text analysis. This can result in the oversimplification of these powerful tools, such as avoiding necessary fine-tuning for convenience or due to a lack of awareness, thereby not leveraging their full capabilities and potentially leading to less desirable results compared with other methods, or ignoring unique challenges related to transparency and replication (as discussed above). It is further crucial to recognize that many advantages attributed to LLMs are also present in other models. For instance, models such as BERT or open-source LLMs can be accessed via APIs, providing a convenient and low-cost option for researchers who cannot self-host these technologies. This makes them comparably accessible and usable without extensive coding or technical expertise. Additionally, OpenAI offers embedding models, such as “text-embedding-ada-3,” that can be used in a similar manner to BERT for downstream tasks. Ultimately, the responsible use of any computational tool hinges on a thorough understanding of its capabilities and the thoughtful consideration of whether it is the most appropriate method for the task at hand. This balanced approach ensures that technological advancements are leveraged effectively and responsibly in research.

## Conclusion

*GPTology*—which we define as the hurried and unjustified application of LLMs either as “replacements” for human participants, or as an off-the-shelf “one-size-fits-all” method in psychological text analysis—can lead to a proliferation of low-quality research, especially if the convenience of using LLMs such as ChatGPT leads researchers to rely too heavily on them. While LLMs, especially fine-tuned ones, can achieve impressive performances on many tasks, the presence of a WEIRD bias, along with the opaque and often irreproducible nature of these models, particularly the proprietary ones, makes them a double-edged sword for psychological research. This does not mean that LLMs are inadequate to aid psychological research, but researchers must actively exercise caution and critically evaluate the limitations of these models before incorporating them into their research paradigms. In the present work, we empirically quantified some of the biases and limitations of these models across multiple psychological domains including moral judgments, personality traits, cultural orientation, and political ideology, among others. Psychological science, which has witnessed multiple adverse consequences when new technologies were haphazardly and heedlessly used, needs to strive for diversification of research samples, validation of different methods against one another, transparency, and ethical considerations in deploying LLMs to ensure that the findings are robust, generalizable, and free from demographic biases. A commitment to rigor and replicability should guide the integration of AI into psychological research, not convenience.

## Supplementary Material

pgae245_Supplementary_Data

## Data Availability

All data, supplementary study materials, and supporting code used in this work are publicly available through an Open Science Framework (OSF) repository at https://doi.org/10.17605/OSF.IO/NAFZY. The repository includes detailed instructions for replicating our findings.

## Preprint

This manuscript was posted as a preprint: https://osf.io/preprints/psyarxiv/d695y.
