# Supplementary material for: Perils and opportunities in using large language models in psychological research
Source: PNAS Nexus. 2024 Jul 16;3(7):pgae245. doi: 10.1093/pnasnexus/pgae245 (PMC11249969; doi:10.1093/pnasnexus/pgae245)
Supplement: pgae245_Supplementary_Data [file pgae245_supplementary_data.pdf]

Supplementary Information

S1. Text Annotations

We compared ChatGPT's automated text annotation performance against a fine-tuned BERT-based model and a Linguistic Inquiry and Word Count (LIWC) based model. We further analyzed whether ChatGPT exhibited biases, that is, whether it aligned more with specific human annotator demographics on the test data.

Methods

We make all data, study materials, and analysis code available at https://osf.io/nafzy/. The repository contains all the necessary instructions to replicate our analyses.

Data. We utilized the Moral Foundations Reddit Corpus (MFRC; Trager et al., 2022b), a collection of 16,123 Reddit comments that have been hand-annotated by at least three trained annotators for 8 categories of moral sentiment (i.e., Care, Proportionality, Equality, Purity, Authority, Loyalty, Thin Morality, Non-morality) based on the updated Moral Foundations Theory (MFT; Atari, Haidt, et al., 2023a) framework. The MFRC further gathered demographic and psychometric information about each human annotator. We randomly divided the MFRC into a test set (2,983 samples) to evaluate the annotation accuracy of ChatGPT and a BERT-based small language model (fine-tuned with a training set of 13,140 samples). See the code repository for instructions to replicate the training and test data.

BERT-model. We used a Bidirectional Encoder Representations from Transformers (BERT)-based (Devlin et al., 2018b) classifier to determine the moral language in each MFRC post using only the post text as input. Specifically, we used the pre-trained BERT model "small BERT" ("bert_uncased_L-12_H-512_A-8"; Turc et al. (2019)) with $L = 12$ hidden layers (i.e., Transformer blocks), a hidden size of $H = 256$, and $A = 4$ attention heads. We then added a downstream (multi-label) classification layer to

the language model to predict which of the eight moral sentiments were expressed in a given post. We simultaneously trained the classification layer and fine-tuned the embedding layers on the training set, which was not used to evaluate the annotation accuracy. Model training and fine-tuning was conducted in python 3.9 (Van Rossum & Drake, 2009) using the tensorflow (v2.12.0; Abadi et al. (2016)) and keras (v2.12.0; Chollet et al. (2015)) libraries. See the code repository for instructions to replicate the training and fine-tuning procedures.

LIWC-model. Supplementing our BERT-based analysis, we leveraged the Linguistic Inquiry and Word Count (LIWC; version 22) to determine moral sentiments in the MFRC texts. LIWC quantifies the frequency of specific word categories, shedding light on underlying emotional, cognitive, and structural markers in textual content (Pennebaker et al., 2001) and is a widely used theory-based psychological text analysis method (Garten et al., 2018; Pennebaker et al., 2015). LIWC allows for analysis regarding the contribution of psychologically validated features and facilitates a comparative analysis between two distinct paradigms: the deep learning approach using BERT's and ChatGPT's contextual, data driven, embeddings and top-down, theory-motivated approach via LIWC. We extracted the LIWC features, spanning 118 dimensions (e.g., linguistic dimensions, psychological processes, personal concerns), using the LIWC-22 software www.liwc.app. We then trained a Support Vector Classifier (SVC; Cortes and Vapnik (1995)), which is a classifier known for its flexibility, its simplicity compared to complex neural networks, and its effectiveness in high-dimensional spaces. We used the SVC to predict moral sentiment using the LIWC features on the same subset of MFRC texts as the BERT model. We trained the model in python 3.10 (Van Rossum & Drake, 2009) using the sklearn (v1.3.0; Pedregosa et al. (2011)) library. See the code repository for instructions to replicate the feature extraction and model training procedure.

ChatGPT. We prompted ChatGPT to annotate posts from the MFRC test data via the OpenAI API (https://platform.openai.com/docs/api-reference?lang=python). To

access the API and collect ChatGPT's text annotations, we used the OpenAI library (v0.27.8; OpenAI (2023)) in Python. We deployed the "gpt-3.5-turbo-0301" model with a temperature of 0 (for maximal deterministic and thus replicable behavior). Temperature is a ChatGPT parameter expressing how words are generated based on the underlying probability distribution computed by the model. A lower temperature indicates that ChatGPT chooses the most likely words when generating a response and a higher temperature indicates that ChatGPT samples words based on their probability (making it more variable and "creative", see https://platform.openai.com/docs/guides/gpt/faq).

For the zero-shot application of ChatGPT, we used the following prompt, which matched the definitions of each moral foundation according to MFT and the human annotators' instructions, to collect the annotations:

"Determine which moral sentiments are expressed in the following text. "care" if the text is about avoiding emotional and physical damage to another individual, "equality" if the text is about equal treatment and equal outcome for individuals, "proportionality" if the text is about individuals getting rewarded in proportion to their merit or contribution, "loyalty" if the text is about cooperating with ingroups and competing with outgroups, "authority" if the text is about deference toward legitimate authorities and the defense of traditions, all of which are seen as providing stability and fending off chaos, "purity" if the text is about avoiding bodily and spiritual contamination and degradation, "thin morality" if the text has a moral sentiment but cannot be categorized as either of the above, "non-moral" if no moral sentiment is expressed in the text. Respond only with these words. Respond with all words that apply, comma separated. Here is the text: [TEXT TO ANNOTATE]"

For the few-shot application of ChatGPT, we add two representative texts as examples for each moral sentiment when prompting the model to determine the presence of moral sentiments in a given text. The examples were chosen from the same data used to fine-tune the BERT model and manually verified to explicitly contain the respective moral

sentiment. Additionally, we split the annotation task into seven separate sub-tasks each indicating whether a specific moral sentiment is or is not expressed in a given text. For example, we ask ChatGPT whether, “care” sentiment is expressed in a text and include two examples of texts that contain “care”. The model then indicates whether the moral sentiment is present in the given text to annotate. We repeat this procedure for each moral sentiment (care, equality, proportionality, loyalty, authority, thin-morality) resulting in a binary list for each text indicating which of the moral sentiments are expressed in this text. This change in procedure was made because adding multiple examples for each of the seven possible moral sentiments would lead to extremely long prompts (task description, sentiment definition, 14 example texts and responses) deteriorating model performance. Additionally, splitting the annotations into separate binary annotation tasks for each sentiment likely improves model performance because the model does not have to factor in other potential sentiments. However, this comes at the cost of increased API requests since the model has to be prompted for each sentiment separately, leading in our case to 7-times higher costs. The following prompt template was used for all moral sentiments:

[ROLE USER]: Determine the presence of [MORAL SENTIMENT] in the following text. The text contains [MORAL SENTIMENT] if [MORAL SENTIMENT DEFINITION]. Respond with “yes” if the sentiment is expressed in the text and “no” if it is not. Respond only with a single word and do not elaborate. Here is the text: [EXAMPLE 1]

[ROLE ASSISTANT]: “yes”

[ROLE USER]: Determine the presence of the moral sentiment of [MORAL SENTIMENT] in the following text: [EXAMPLE 2]

[ROLE ASSISTANT]: “yes”

[ROLE USER]: Determine the presence of the moral sentiment of [MORAL SENTIMENT] in the following text: [TEXT TO ANNOTATE]

Note that [ROLE USER] and [ROLE ASSISTANT] are API parameter, indicating whether a prompt is the input of a user or the output of the model. This is necessary for

the few-shot examples to show the model what model output should be given to a user's request.

For fine-tuning ChatGPT, we utilize OpenAI's dedicated service documented at https://platform.openai.com/docs/guides/fine-tuning. Due to the service's current limitations, our customization was restricted to setting the number of epochs. We chose the default setting of $epoch = 2$ appropriate for our dataset size ($N = 13,140$). Although ChatGPT and BERT were both fine-tuned on identical data, their data processing methods differ: BERT processes raw texts and emits a binary vector representing the presence of various moral sentiments, whereas ChatGPT relies on a prompt-response mechanism. Therefore, we transformed our training texts into prompts, each directing the model to detect moral sentiments in a given text and converted the annotation labels to comma-separated lists indicating the moral sentiments present, e.g., *"care,loyalty"*. The specific template employed was:

[ROLE USER]: Determine which moral sentiments are expressed in the following text. The text contains "care" if the text is about avoiding emotional and physical damage to another individual, "equality" if the text is about equal treatment and equal outcome for individuals, "proportionality" if the text is about individuals getting rewarded in proportion to their merit or contribution, "loyalty" if the text is about cooperating with ingroups and competing with outgroups, "authority" if the text is about deference toward legitimate authorities and the defense of traditions, all of which are seen as providing stability and fending off chaos, "purity" if the text is about avoiding bodily and spiritual contamination and degradation, "thin morality" if the text has a moral sentiment but cannot be categorized as either of the above, "non-moral" if no moral sentiment is expressed in the text. Respond only with these words. Respond with all words that apply, comma separated. Here is the text: [TEXT TO ANNOTATE]

[ROLE ASSISTANT]: [MORAL SENTIMENTS COMMA-SEPARATED]

Performance Evaluation. To evaluate the annotation accuracy of the BERT-based model, LIWC-based model, and ChatGPT (zero-shot, few-shot, fine-tuned), we calculated each model's F1 score (Goutte & Gaussier, 2005), which is a widely used single metric that assesses the model's ability to make accurate positive predictions while minimizing false positives and false negatives. We calculated the F1 scores with the human annotations as ground truth using the Sklearn library (v1.3.0; Pedregosa et al. (2011)) in Python. We calculated the macro averaged F1 score, that is, the unweighted average of the F1 scores for the prediction of each type of moral sentiment, which expresses the model's accuracy across all moral sentiments. Finally, we fit a logistic regression model predicting the presence of moral sentiment in an annotation (binary, yes/no) as a function of annotator type (Human, ChatGPT, BERT, LIWC). This model expressed how much more likely the computational models were to annotate a moral sentiment compared to human ground truth. This model was used to evaluate whether ChatGPT, BERT, or LIWC significantly over-predicted or under-predicted (e.g., false positives, false negatives) the specific moral sentiments.

We conducted all statistical analyses in R (v4.1.2; R Core Team (2021)) using the stats (v4.1.2; R Core Team (2021)) package (R Core Team, 2021). See the code repository for instructions to replicate the statistical analyses.

Bias Analyses. To determine biases in ChatGPT's annotations, we investigated how much ChatGPT aligned with annotators of specific demographics. To that end, we create a binary variable expressing whether ChatGPT's annotation and the human annotators' rating aligned (1 if a ChatGPT and human annotation were equal; 0 if not). If there were multiple sentiments in one post, we compared each one separately. We then fit logistic regression models that predicted human-ChatGPT alignment as a function of annotator demographics and psychometric information. These models describe how likely ChatGPT is to align with an annotator of certain demographics (e.g., race, gender, age). In total, we tested for biases toward annotators' age, sex, religion, moral values (based on

MFT), personality (Five-Factor Model), creativity (creative imagination, aesthetic sensitivity, intellectual curiosity), mental health (anxiety, depression, emotional volatility), political orientation (collectivism, individualism, cultural-tightness, social-conservatism, economical-conservatism), interpersonal attitudes (compassion, trust, respectfulness), and efficiency (productiveness, organization, responsibility).

We conducted all statistical analyses in R (v4.1.2; R Core Team (2021)) using the stats (v4.1.2; R Core Team (2021)) package (R Core Team, 2021). See the code repository for instructions to replicate the statistical analyses.

Results

Overall, we found that fine-tuned BERT outperformed zeroshot-ChatGPT by a large margin. Specifically, BERT achieved an F1 score of 0.49, while ChatGPT achieved an F1 score of 0.22. Additionally, ChatGPT was more extreme in over or under-predicting the moral sentiments. Notably, ChatGPT extremely under-predicted nonmoral texts. Essentially, ChatGPT classified every text as moral. Additionally, it vastly over-classified “Thin-morality”, which may be connected to its over-sensitivity to moral language detection (e.g., nonmoral texts are misclassified as “Thin-morality”). See Table S2 for an overview of BERT and ChatGPT deviation in class distributions from human ground truth. Interestingly, even the LIWC-based approach outperformed ChatGPT with an F1 score of 0.27 and LIWC was significantly less likely and less extreme to over-predict or under-predict a moral sentiment except for Care and Loyalty, comparable to the BERT-based model. Note, that we did not conduct any elaborate preprocessing or parameter tuning (beyond the regularization weights) or attempted to train more powerful complex classifiers for the LIWC-based method. Additionally, the foundation-level F1 scores in Table S1 show that zero-shot ChatGPT completely failed to capture most foundations (Proportionality, Loyalty, Authority, Authority, Purity, thin-morality, non-moral), with F1 scores of near zero on Purity and recognizing non-moral posts.

Applying ChatGPT with more elaborate methods, such as few-shot prompting or outright fine-tuning, noticeably increased model performance. Fewshot-ChatGPT achieved an F1 score of 0.32 and fine-tuned ChatGPT achieved an F1 score of 0.54 even surpassing fine-tuned BERT. The foundation-level F1 scores show that fine-tuned ChatGPT performed best on most foundations, being on par with BERT on all foundations and outperforming it substantially on two (+10% F1 score increase on Care, +25% F1 score increase on Loyalty), while BERT was slightly better only on the Authority and Proportionality foundations. Furthermore, fine-tuned and few-shot ChatGPT were in most cases less extreme in their deviations from ground truth compared to the zero-shot application. Notably, fewshot-ChatGPT improves zeroshot-ChatGPT's vast overestimation of the presence of Thin-morality (+1,240%) to -70% and fine-tuned ChatGPT further improved this to -32%. Similarly, fewshot-ChatGPT improved zeroshot-ChatGPT's deviation from ground truth of nearly 100 percent on nonmoral texts (essentially detecting a moral sentiment in nearly all posts) to +90% and fine-tuned ChatGPT further improved upon this to -27% deviation. However, applying ChatGPT in a few-shot setting still failed to capture several foundations (Loyalty, Authority, Purity, Eqyality, Thin-morality), with near zero F1 scores on Loyalty, Equality, and Thin-morality. Lastly, Table S3 shows that our fine-tuned models outperform the zero-shot application of even the newest flagship models, GPT-4 and GPT-4-Turbo, as reported in Rathje et al. (2023).

Our results reveal a nuanced reality. While LLMs hold profound potential, their application in a zero-shot or few-shot setting can result in dramatic failures, underscoring the limitations of these models when used straight 'out-of-the-box.' Conversely, our findings also highlight that fine-tuning LLMs can drastically improve their performance, surpassing previously established methods. This emphasizes the importance of not simply choosing the most convenient option, such as assuming LLMs will automatically adapt to specific annotation tasks in a zero- or few-shot setting. Instead, it is crucial to validate LLMs performance on task-by-task basis and, if necessary, invest in fine-tuning. However,

note that fine-tuning BERT is significantly less resource-intensive and cheaper than fine-tuning ChatGPT which is also restricted to OpenAI's online platform and currently does not support modifications to their fine-tuning procedure.

Table S1

Classifier performance (F1 score) by prediction class

Class	BERT	GPT-FT	GPT-Few	GPT-Zero	LIWC
Care	0.58	**0.69**	0.55	0.55	0.40
Proportionality	0.43	0.42	**0.52**	0.15	0.14
Loyalty	0.23	**0.49**	0.09	0.24	0.11
Authority	**0.30**	0.26	0.19	0.23	0.11
Purity	0.42	**0.48**	0.21	0.03	0.06
Equality	0.63	**0.66**	0.10	0.41	0.35
Thin Morality	0.41	**0.43**	0.08	0.17	0.18
Non-moral	0.86	**0.89**	0.81	0.00	0.78

Note. Table shows F1 scores for each classifier by prediction class. The best model for each class is highlighted in bold. Table shows that fine-tuned ChatGPT performs best in all but two foundations. For the Care and Loyalty foundation, the performance increase is substantial even compared to fine-tuned BERT.

Demographic Biases. We found that ChatGPT showed significant demographic biases in it's moral annotations, aligning more so with certain groups, including younger over older (-1%, p = .033), female over male (+286 %, $p < .001$), and Christian over non-religious (-67%, $p < .001$). See Table S4 for an overview of demographic biases.

Moral Biases. We found that ChatGPT showed significant moral biases in it's annotations, aligning more so with human annotators who endorse certain moral values,

Table S2

Deviations from ground truth class distribution for each model on each prediction class

Class	BERT	LIWC	ChatGPT (Zero)	ChatGPT (Few)	ChatGPT (FT)
Care	+12% ($p = .162$)	-54% ($p < .001$)	+35% ($p < .001$)	6% ($p = .443$)	**-5% ($p = .536$)**
Equality	-11% ($p = .255$)	-23% ($p = .013$)	+55% ($p < .001$)	**+4% ($p = .701$)**	-24 ($p = .007$)
Proportionality	-20% ($p = .118$)	**-19% ($p = 0.136$)**	-55% ($p < .001$)	-86% ($p < .001$)	-48% ($p < .001$)
Loyalty	-64% ($p < .001$)	-28% ($p = .031$)	**+3% ($p < .836$)**	-64% ($p < .001$)	-45 ($p < .001$)
Authority	**-32% ($p = .003$)**	-36% ($p = .001$)	+96% ($p < .001$)	-38% ($p < .001$)	-67% ($p < .001$)
Purity	-74% ($p < .001$)	**-34% ($p = .049$)**	-86% ($p < .001$)	-59% ($p < .001$)	-59% ($p < .001$)
Thin-Morality	**+2% ($p = .861$)**	-38% ($p < .001$)	+1,240% ($p < .001$)	-70% ($p < .001$)	-31% ($p < .001$)
Nonmoral	**+18% ($p = .002$)**	+74% ($p < .001$)	-100% ($p < .001$)	+90% ($p < .001$)	+27 ($p < .001$)

Note. *Table shows how similar the distribution of moral foundations in the classifier outputs are to the distribution in human annotations. Table reports the difference in odds to predict a moral sentiment for each classifier (BERT, LIWC, ChatGPT) compared to the ground truth of trained human annotators. Table shows that BERT deviates significantly less from ground truth distributions compared to zeroshot-ChatGPT (except on the Loyalty foundation) and is comparable to LIWC. For half of the moral sentiments, BERT does* ***not*** *deviate significantly (at $p < .05$) from ground-truth while zeroshot-ChatGPT deviates significantly from ground-truth for all moral sentiments except Loyalty. Table further shows that few-shot (Few) and fine-tuning (FT) improves ChatGPT performance. However, the deviations remain higher than BERT and in all but three cases (care, equality, loyalty).*

such as care (+31,159%, $p < .001$) and proportionality (+21,215%, $p < .001$), while misaligning with the annotators who endorse other values, such as loyalty (-96%, $p < .001$) and equality (-97%, $p < .001$). See Table S5 for an overview of moral biases.

Personality Biases. We found that ChatGPT showed significant personality biases in it's annotations, aligning more so with human annotators who load highly on certain personality traits, such as openness (+1244%, $p < .001$) and agreeableness (+156%, $p < .001$), while misaligning with annotators who load highly on other personality traits, such as conscientiousness (-49%, $p < .001$) and extraversion(-68%, $p < .001$). See Table S6 for an overview of personality biases.

Table S3

Classifier performance (F1 score) by prediction class

Class	BERT	GPT-3.5 (FT)	GPT-3.5 (zero)	GPT-4	GPT-4-Turbo
Care	0.58	**0.69**	0.55	0.53	0.50
Proportionality	**0.43**	0.42	0.15	0.17	0.13
Loyalty	0.23	**0.49**	0.24	0.34	0.26
Authority	**0.30**	0.26	0.23	0.21	0.09
Purity	0.42	**0.48**	0.03	0.14	0.14
Equality	0.63	**0.66**	0.41	0.36	0.24
Thin Morality	0.41	**0.43**	0.17	-	-
Non-moral	0.86	**0.89**	0.00	0.65	0.68

Note. *Table compares our fine-tuned BERT and GPT-3.5 model with the GPT-4 models in Rathje et al. (2023). Note that for GPT-4 and GPT-4-Turbo no performances on "Thin-Morality" were reported. Table shows that fine-tuned models outperform the zero-shot models on all moral foundations, even when they are significantly smaller models (BERT) or older, less powerful versions (GPT-3.5).*

Table S4

Demographic Biases of ChatGPT annotations on the MFRC

Demographic Variable	$\Delta odds$	p
Female (vs. Male)	+286 %	$< .001$
Age (years)	-1%	.033
Non-Religious (vs. Christianity)	-67%	$< .001$

Note. *Table shows ChatGPT bias towards respective demographics during annotations. That is, ChatGPT had higher (+) or lower (-) odds (%) of aligning with human annotators of said demographic.*

Table S5

Moral Biases of ChatGPT annotations on the MFRC

Moral Foundation	$\Delta odds$	p
Care	+31,159%	< .001
Equality	-97%	< .001
Proportionality	+21,215%	< .001
Loyalty	-96%	< .001
Authority	+21%	.329
Purity	-	-

Note. *Table shows ChatGPT bias towards respective moral profiles during annotations. That is, it had higher/lower odds (%) of aligning with human annotators endorsing said moral values. All variables are 5-point Likert scales.*

Table S6

Personality Biases of ChatGPT annotations on the MFRC

Personality Variable	$\Delta odds$	p
Openness	+1244%	< .001
Conscientiousness	-49%	< .001
Extraversion	-68%	< .001
Agreeableness	+156%	< .001
Neuroticism	-15%	.327

Note. *Table shows ChatGPT bias towards respective demographics during annotations. That is, ChatGPT had higher (+) or lower (-) odds (%) of aligning with human annotators of said personalities. All variables are 5-point Likert scales.*

Socio-Political Biases. We found that ChatGPT showed significant socio-political biases in it's annotations, aligning more so with human annotators who have certain political attitudes, such as individualism (+142%, $p < .001$), while misaligning with annotators who have other political attitudes, such as collectivism (-30%, $p < .001$) tightness (-77%, $p < .001$), social conservatism (-4%, $p < .001$), and economic conservatism (-2%, $p < .001$). See Table S7 for an overview of political biases.

Table S7

Political Biases of ChatGPT annotations on the MFRC

Socio-Political Variable	$\Delta odds$	p
Collectivism	-30%	< .001
Individualism	+142%	< .001
Tightness	-77%	< .001
Social conservatism	-4%	< .001
Economical conservatism	-2%	< .001

Note. Table shows ChatGPT bias towards respective demographics during annotations. That is, ChatGPT had higher (+) or lower (-) odds (%) of aligning with human annotators of said political values. All variables are 5-point Likert scales.

Creativity-related Biases. We found that ChatGPT showed significant creativity-related biases in its annotations, aligning more so with human annotators who have certain traits, such as creative imagination (+1,351%, $p < .001$), while misaligning with the annotators who have other traits, such as openness (-50%, $p = .006$) and aesthetic sensitivity (-61%, $p < .001$). See Table S8 for an overview of creativity-related biases.

Mental Health Biases. We found that ChatGPT showed significant mental health biases in it's annotations, aligning more so with human annotators who have certain mental health traits, such as anxiety (+95%, $p < .001$) and emotional-volatility +46%, $p < .001$), while misaligning with the annotators who have other mental health traits

Table S8

Creativity-related Biases of ChatGPT annotations on the MFRC

Variable	$\Delta odds$	p
Openness	-50%	.006
Creative imagination	+1,351%	< .001
Aesthetic sensitivity	-61%	< .001

Note. *Table shows ChatGPT bias towards respective demographics during annotations. That is, ChatGPT had higher (+) or lower (-) odds (%) of aligning with human annotators of said creative inclinations. All variables are 5-point Likert scales.*

(depression (-36%, $p < .001$). See Table S9 for an overview of mental health related biases.

Table S9

Mental-Health related Biases of ChatGPT annotations on the MFRC

Mental-Health Variable	$\Delta odds$	p
Anxiety	+95%	< .001
Depression	-36%	< .001
Emotional-Volatility	+46%	< .001

Note. *Table shows ChatGPT bias towards respective demographics during annotations. That is, ChatGPT had higher (+) or lower (-) odds (%) of aligning with human annotators with said mental-health issues. All variables as 5-point Likert scales.*

Efficiency-related Biases. We found that ChatGPT showed significant efficiency-related trait biases in it's annotations, aligning more so with human annotators who have low endorsement of all efficiency-related traits, such as productiveness (-18%, $p < .001$), organization (-62%, $p < .001$) and responsibility (-52%, $p < .001$). See Table S10 for an overview of efficiency-related biases.

Table S10

Efficiency-related Biases of ChatGPT annotations on the MFRC

Efficiency Variable	$\Delta odds$	p
Productiveness	-18%	$< .001$
Organization	-62%	$< .001$
Responsibility	-52%	$< .001$

Note. Table shows ChatGPT bias towards respective demographics during annotations. That is, ChatGPT had higher (+) or lower (-) odds (%) of aligning with human annotators of said efficiency traits. All variables as 5-point Likert scales.

S2. Survey Responses

We collected responses to wide range of psychological questionnaires using ChatGPT and compared the responses to human data covering a wide range of demographic groups. We specifically, analyzed to what extent ChatGPT mirrored meaningful human data and whether it was skewed towards or against specific demographics.

Methods

Data. We conducted our tests on various questionnaires covering a broad range of psychologically relevant fields, such as personality, morality, cognition, and social and political attitudes. Specifically, we used the Big-Five Inventory (BFI; $N = 3924$; Fossati et al. (2011)), Need for Closure Survey ($N = 315$; Webster and Kruglanski (1994b)), Need for Cognition Survey ($N = 900$; Cacioppo and Petty (1982b)), Right-wing-authoritarianism Scale ($N = 1020$; Zakrisson (2005b)), Emphasizing-Systemizing Scale ($N = 3141$; Baron-Cohen et al. (2003b)), and Rational-Experiential Inventory Scale ($N = 1456$; Pacini and Epstein (1999b)). The human response data was collected on https://www.yourmorals.org and contains the participants' responses as well as various demographic information about the participants.

ChatGPT Responses. We prompted ChatGPT to respond to survey items via the OpenAI API (https://platform.openai.com/docs/api-reference?lang=python). To access the API and to collect the responses we used the OpenAI library (v0.27.8; OpenAI (2023)) in Python. We deployed the "gpt-3.5-turbo-0301" model with a temperature of 1 (to allow for maximal variability in the responses). The prompts mirrored the study instructions given to human respondents and had the general shape of "For a scientific study, please indicate your level of agreement with the following statement. Use integer numbers from 1 to 5, with 1 meaning strongly disagree, and 5 meaning strongly agree. Respond with a single number. The statement is: [Survey item]"

We adapted the scale descriptions to the surveys as necessary. We collected 100 responses for each item (that is, we repeated the same prompt 100 times) to allow for analyses regarding the variance within ChatGPT responses. See the project repository for the utilized survey items, ChatGPT prompts, and code containing the API calls.

Bias Analyses. First, we compared ChatGPT's responses to demographic-level average human responses. That is, we investigated the difference between ChatGPT's survey responses and the average response of different groups of humans based on their demographics (e.g., age, race, gender). Note, we removed all demographics with less than 100 samples in the data sets as they did not provide enough information to make inferences about said demographic. We calculated the average ChatGPT response for each construct in a given survey and then subtracted it from the group-level average human response on said construct (e.g., the difference between ChatGPT's "Openness" score and the average male or female "Openness" score). We then tested these differences for statistical significance using Dunnett's test (Dunnett, 1955), which compares each of a number of treatments with a single control, with the demographic groups as treatments and ChatGPT as the control, while adjusting for multiple comparisons. We then repeated this analysis for differences in variance. We investigated whether ChatGPT responses had less variance compared to the different human groups (e.g., the difference in the variance of ChatGPT's

"Openness" scores vs. the variance in male or female "Openness" scores). We used the Levene Test (Levene, 1960) to determine whether the variance in each human group's responses differed from ChatGPT's while accounting for multiple comparisons using Bonferroni correction. Second, we investigated the relationship between the participants' demographic characteristics and the similarity of human to ChatGPT responses. We fit a linear regression model that predicted the absolute deviation of ChatGPT and human responses (aggregated over all survey constructs) as a function of each human participant's demographic characteristic. These models expressed which participant demographics ChatGPT is more or less likely to align with and therefore showed its biases (e.g., are ChatGPT's responses more similar to older vs. younger participants).

We conducted all analyses in R (v4.1.2; R Core Team (2021)) using the stats (v4.1.2; R Core Team (2021)), car (v3.0-11; Fox and Weisberg (2019)), and DescTools (v0.99.44; Andri et mult. al. (2021)) packages.

Results

Overall, we found that ChatGPT responses significantly differed from a wide range of human demographics and in some cases produced wildly unnatural data. For example, across questionnaires and psychological constructs, ChatGPT produced significantly less variance than human subjects. On some questionnaires, such as the BFI and Need for Cognition questionnaires, ChatGPT produced responses significantly deviating from the human mean (e.g., ChatGPT was unnaturally high on Agreeableness, Conscientiousness and low on Neuroticism). Furthermore, ChatGPT was biased towards a range of demographics on most surveys, that is, ChatGPT's responses were more similar to some demographics than others. See Tables S11 - S26 and Figures S1 - S28 for an overview of ChatGPT and human differences across the various questionnaires.

Differences to political orientations. We found that ChatGPT significantly deviated from responses of various political groups on most surveys. For example,

ChatGPT was more agreeable and conscientious than liberals, moderates, and conservatives when responding to the BFI or had lower Need for Cognition than liberals, moderates, and conservatives when responding to the Need for Cognition scale. See Table S11 and Figures S1-S6) for on overview of ChatGPT's deviations from political groups across all surveys.

Table S11

Deviation of ChatGPT responses from human responses grouped by political orientation

Survey	Construct	Demographic	*d*	*p*
BFI	Extraversion	Liberal	0.017	.978
BFI	Agreeableness	Liberal	-0.230	< .001
BFI	Conscientiousness	Liberal	-0.541	< .001
BFI	Neuroticism	Liberal	0.780	< .001
BFI	Openness	Liberal	0.444	< .001
Need for Cognition	Need for Cognition	Liberal	0.659	< .001
Need for Closure	Need for Closure	Liberal	-0.004	.071
Systemizing and Emphasizing	Systemizing	Liberal	-0.318	< .001
Systemizing and Emphasizing	Emphasizing	Liberal	0.201	< .001
Rational-Experiential Inventory	Rational	Liberal	0.435	< .001
Rational-Experiential Inventory	Experiential	Liberal	0.123	.166
Right-Wing-Authoritarianism	Right-Wing-Authoritarianism	Liberal	-0.226	.003
BFI	Extraversion	Moderate	0.042	.806
BFI	Agreeableness	Moderate	-0.284	< .001
BFI	Conscientiousness	Moderate	-0.416	< .001
BFI	Neuroticism	Moderate	0.567	< .001
BFI	Openness	Moderate	0.316	< .001
Need for Cognition	Need for Cognition	Moderate	0.544	< .001
Need for Closure	Need for Closure	Moderate	-	-
Systemizing and Emphasizing	Systemizing	Moderate	-0.263	< .001

Continued on next page

Table S11 – continued from previous page

**Survey**	**Construct**	**Demographic**	***d***	***p***
Systemizing and Emphasizing	Emphasizing	Moderate	0.09	.091
Rational-Experiential Inventory	Rational	Moderate	0.344	$< .001$
Rational-Experiential Inventory	Experiential	Moderate	0.159	.059
Right-Wing-Authoritarianism	Right-Wing-Authoritarianism	Moderate	0.522	$< .001$
BFI	Extraversion	Conservative	0.061	.672
BFI	Agreeableness	Conservative	-0.403	$< .001$
BFI	Conscientiousness	Conservative	-0.378	$< .001$
BFI	Neuroticism	Conservative	0.451	$< .001$
BFI	Openness	Conservative	0.183	.005
Need for Cognition	Need for Cognition	Conservative	0.563	$< .001$
Need for Closure	Need for Closure	Conservative	-	-
Systemizing and Emphasizing	Systemizing	Conservative	-0.187	$< .001$
Systemizing and Emphasizing	Emphasizing	Conservative	-0.018	.909
Rational-Experiential Inventory	Rational	Conservative	0.432	$< .001$
Rational-Experiential Inventory	Experiential	Conservative	0.192	.045
Right-Wing-Authoritarianism	Right-Wing-Authoritarianism	Conservative	1.621	$< .001$

Note: *Positive values indicate a higher average response for the political group compared to ChatGPT. Significance determined via Dunnett's Test.*

Figure S1

Comparing ChatGPT against Humans grouped by political opinion for responses on the Big Five personality questionnaire

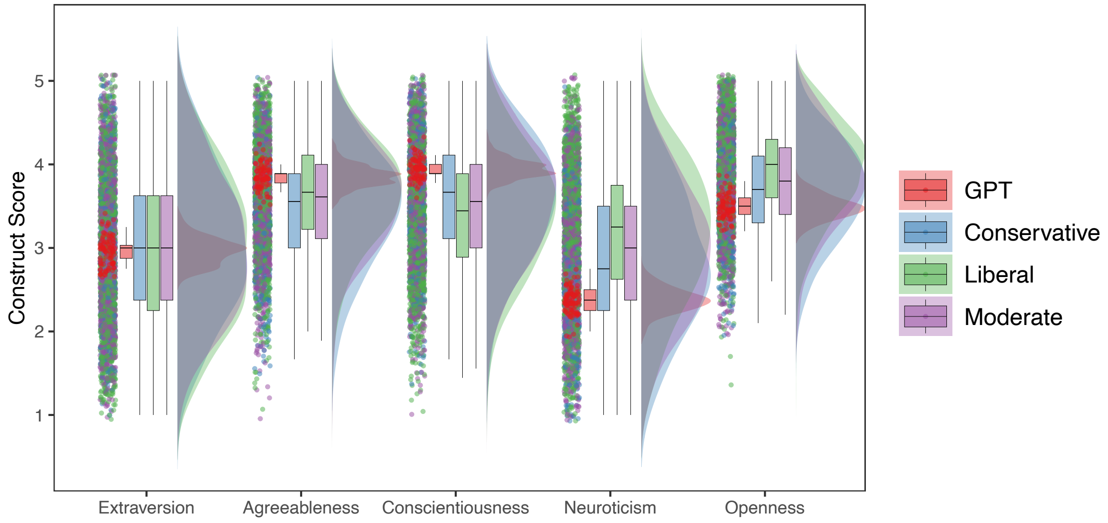

Figure S2

Comparing ChatGPT against Humans grouped by political opinion for responses on the Need for Cognition questionnaire

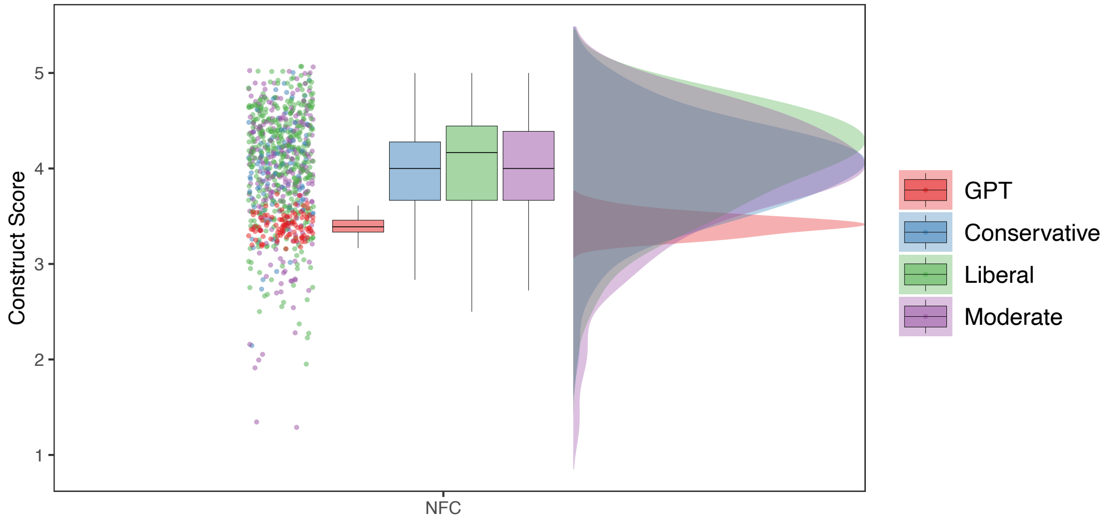

Figure S3

Comparing ChatGPT against Humans grouped by political opinion for responses on the Need for Closure questionnaire

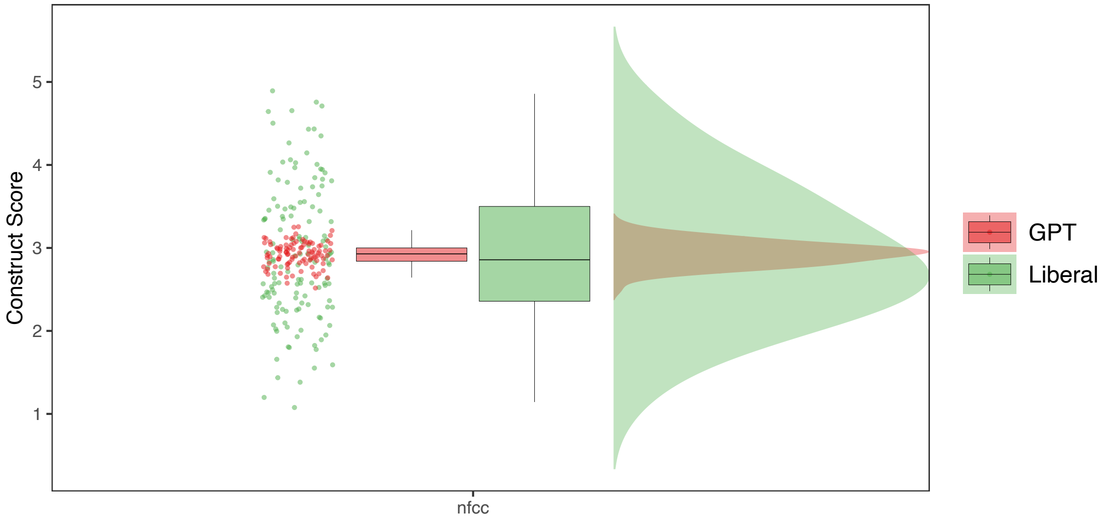

Figure S4

Comparing ChatGPT against Humans grouped by political opinion for responses on the Systematizing-Emphasizing scale

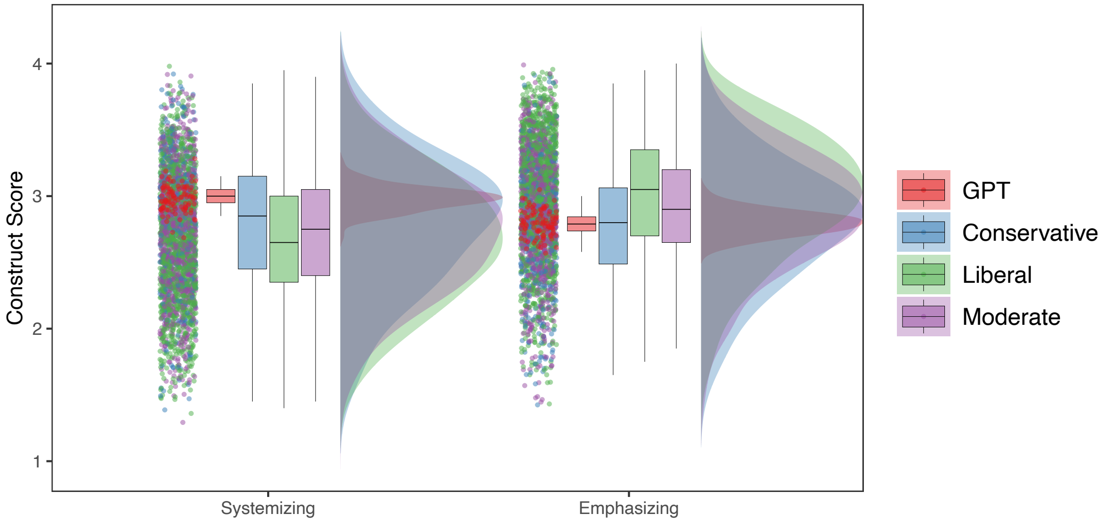

Figure S5

Comparing ChatGPT against Humans grouped by political opinion for responses on the Rational-Experiential Inventory questionnaire

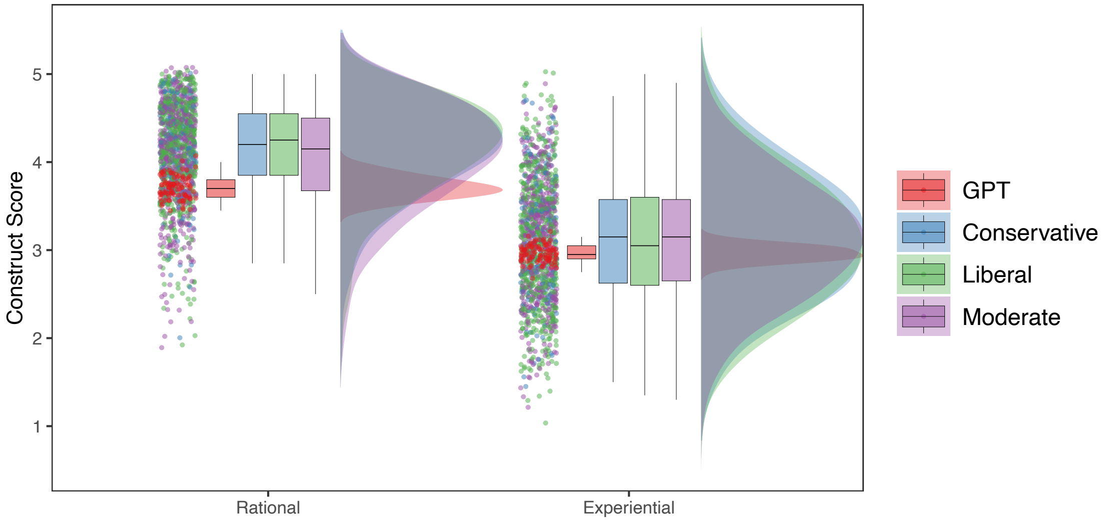

Figure S6

Comparing ChatGPT against Humans grouped by political opinion for responses on the Right-Wing-Authoritarianism questionnaire

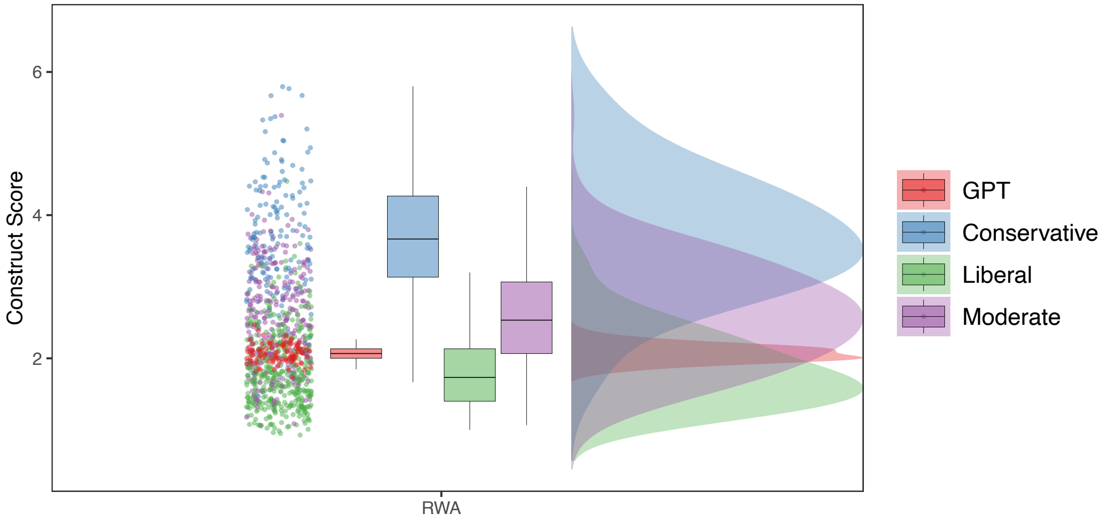

Differences to sexes. We found that ChatGPT significantly deviated from responses of both sexes on most surveys. For example, ChatGPT gave responses that are less neurotic and open-minded while more agreeable and conscientious than both Males and Females on the BFI or had lower right-wing-authoritarianism scores than both Males and Females. See Table S13 and Figures S13-S12 for on overview of ChatGPT's deviations from Males and Females across all surveys.

Figure S7

Comparing ChatGPT against Humans grouped by Sex for responses on the Big Five personality questionnaire

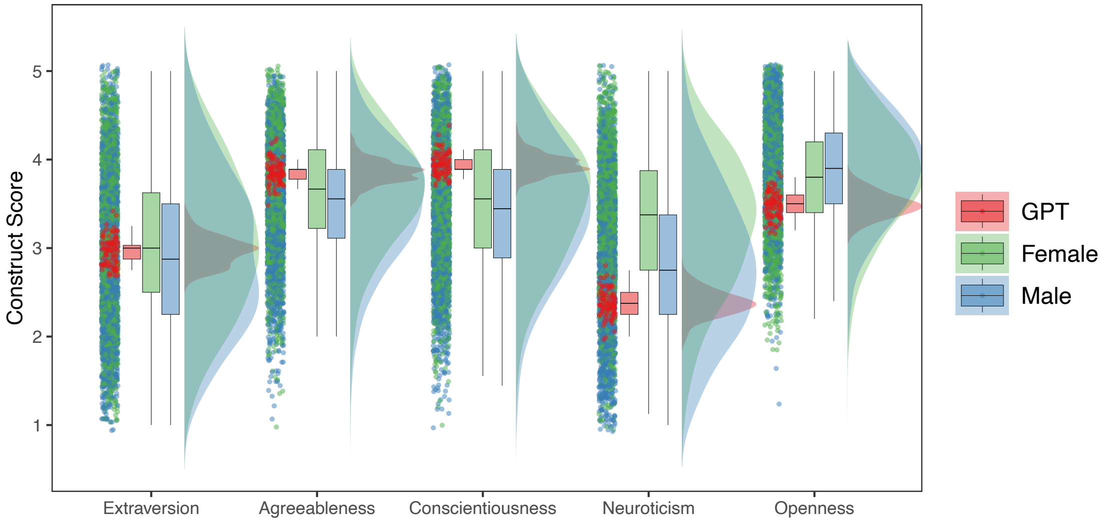

Table S13

Deviation of ChatGPT responses from human responses grouped by sex

Survey	Construct	Demographic	d	p
BFI	Extraversion	Female	0.102	.277
BFI	Agreeableness	Female	-0.206	.002
BFI	Conscientiousness	Female	-0.405	$< .001$
BFI	Neuroticism	Female	0.886	$< .001$
BFI	Openness	Female	0.311	$< .001$
Need for Cognition	Need for Cognition	Female	0.693	$< .001$
Need for Closure	Need for Closure	Female	0.124	.757
Systemizing and Emphasizing	Systemizing	Female	-0.494	$< .001$
Systemizing and Emphasizing	Emphasizing	Female	0.244	$< .001$
Rational-Experiential Inventory	Rational	Female	0.277	$< .001$
Rational-Experiential Inventory	Experiential	Female	0.302	$< .001$
Right-Wing-Authoritarianism	Right-Wing-Authoritarianism	Female	0.157	.188
BFI	Extraversion	Male	-0.043	.692
BFI	Agreeableness	Male	-0.384	$< .001$
BFI	Conscientiousness	Male	-0.532	$< .001$
BFI	Neuroticism	Male	0.435	$< .001$
BFI	Openness	Male	0.416	$< .001$
Need for Cognition	Need for Cognition	Male	0.531	$< .001$
Need for Closure	Need for Closure	Male	0.050	.260
Systemizing and Emphasizing	Systemizing	Male	0.105	.020
Systemizing and Emphasizing	Emphasizing	Male	-0.004	.985
Rational-Experiential Inventory	Rational	Male	0.502	$< .001$
Rational-Experiential Inventory	Experiential	Male	0.049	.601
Right-Wing-Authoritarianism	Right-Wing-Authoritarianism	Male	0.441	$< .001$

Note: *Positive values indicate a higher average response for the sex category compared to ChatGPT. Significance determined via Dunnett's Test.*

Figure S8

Comparing ChatGPT against Humans grouped by sex for responses on the Need for Cognition questionnaire

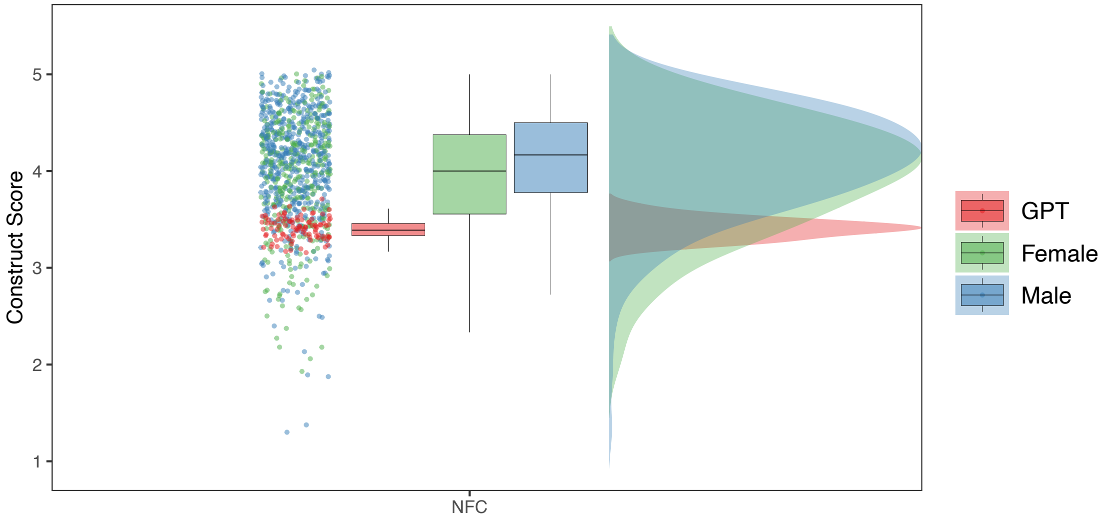

Figure S9

Comparing ChatGPT against Humans grouped by sex for responses on the Need for Closure questionnaire

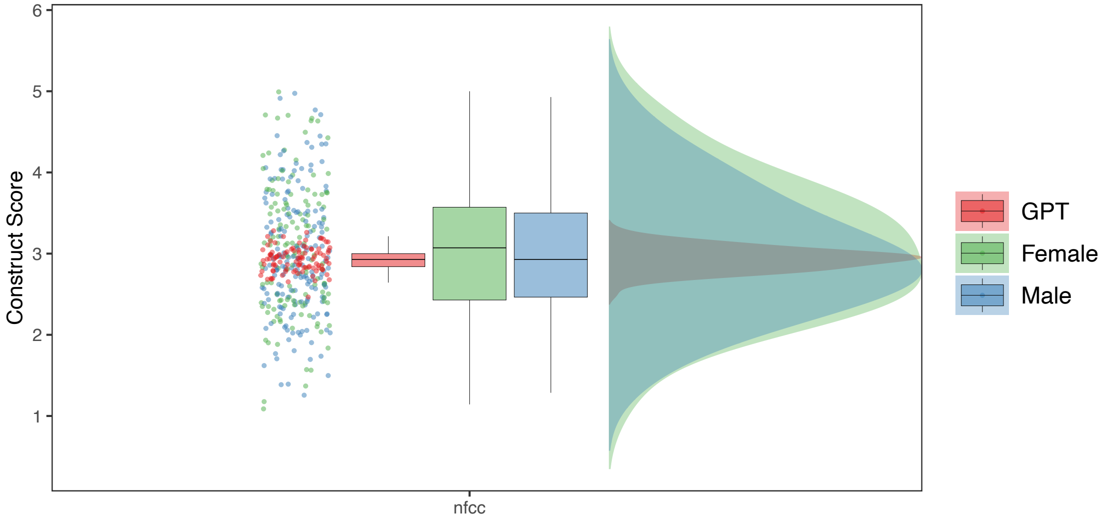

Figure S10

Comparing ChatGPT against Humans grouped by sex for responses on the Systematizing and Emphasizing questionnaire

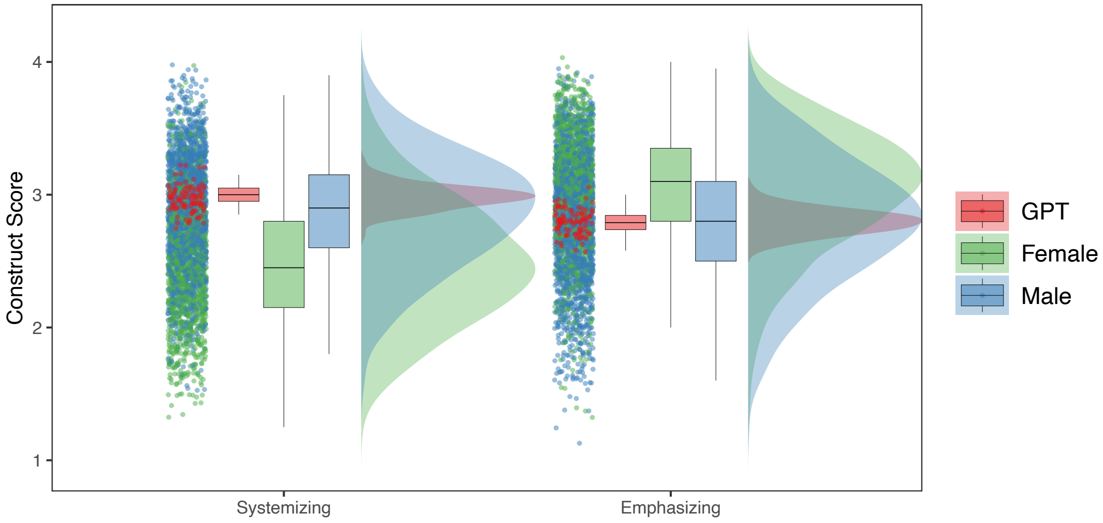

Figure S11

Comparing ChatGPT against Humans grouped by sex for responses on the Rational-Experiential Inventory questionnaire

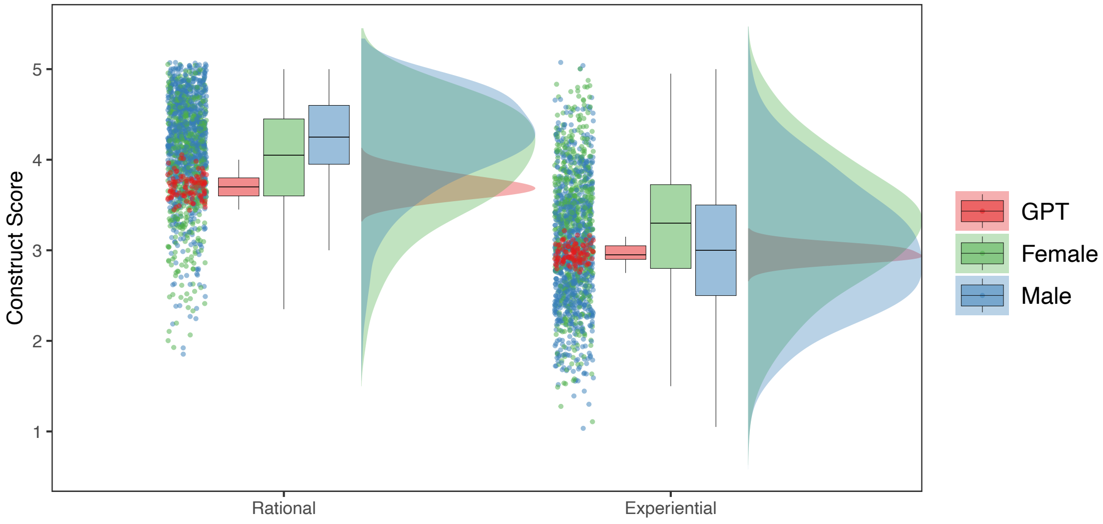

Figure S12

Comparing ChatGPT against Humans grouped by sex for responses on the Right-Wing-Authoritarianism questionnaire

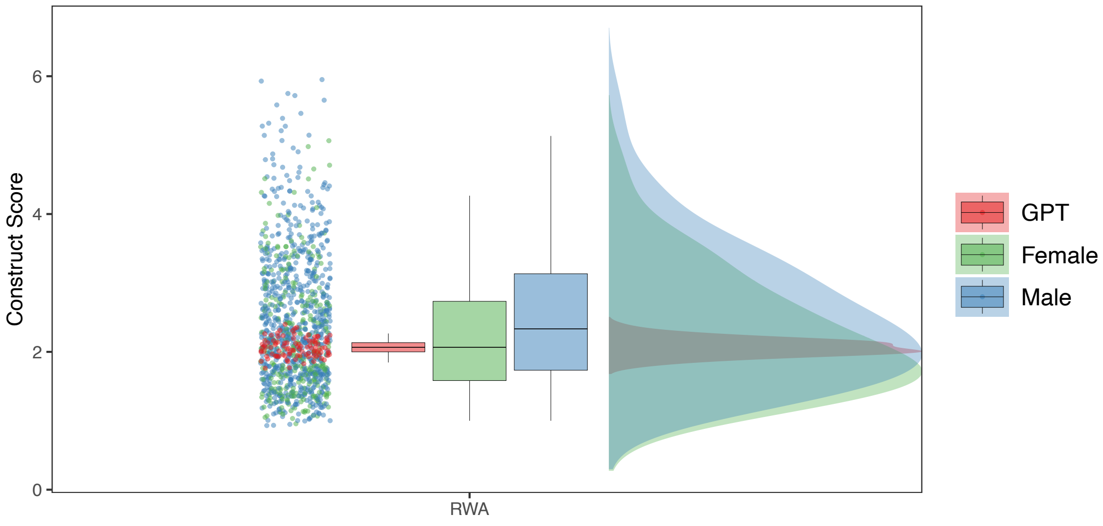

Differences to racial groups. We found that ChatGPT significantly deviated from responses of various racial groups on most surveys. For example, ChatGPT had lower experiential scores than White but not Asian participants or less neurotic and open-minded while more agreeable and conscientious than all races. See Table S14 and Figures S13-S18 for on overview of ChatGPT's deviations from racial groups across all surveys.

Table S14

Deviation of ChatGPT responses from human responses grouped by race

**Survey**	**Construct**	**Demographic**	***d***	***p***
BFI	Extraversion	Asian	-0.078	.717
BFI	Agreeableness	Asian	-0.422	$< .001$
BFI	Conscientiousness	Asian	-0.623	$< .001$
BFI	Neuroticism	Asian	0.645	$< .001$
BFI	Openness	Asian	0.263	$< .001$
Need for Cognition	Need for Cognition	Asian	-	-
Need for Closure	Need for Closure	Asian	-	-
Systemizing and Emphasizing	Systemizing	Asian	-0.259	$< .001$
Systemizing and Emphasizing	Emphasizing	Asian	0.042	.718
Rational-Experiential Inventory	Rational	Asian	0.263	$< .001$
Rational-Experiential Inventory	Experiential	Asian	0.092	.413
Right-Wing-Authoritarianism	Right-Wing-Authoritarianism	Asian	-	-
BFI	Extraversion	White	0.039	.940
BFI	Agreeableness	White	-0.287	$< .001$
BFI	Conscientiousness	White	-0.448	$< .001$
BFI	Neuroticism	White	0.630	$< .001$
BFI	Openness	White	0.396	$< .001$
Need for Cognition	Need for Cognition	White	0.668	$< .001$
Need for Closure	Need for Closure	White	-	-
Systemizing and Emphasizing	Systemizing	White	-0.251	$< .001$

Continued on next page

Table S14 – continued from previous page

**Survey**	**Construct**	**Demographic**	***d***	***p***
Systemizing and Emphasizing	Emphasizing	White	0.100	.074
Rational-Experiential Inventory	Rational	White	0.460	< .001
Rational-Experiential Inventory	Experiential	White	0.167	.027
Right-Wing-Authoritarianism	Right-Wing-Authoritarianism	White	0.347	< .001
BFI	Extraversion	Hispanic	0.013	.996
BFI	Agreeableness	Hispanic	-0.370	< .001
BFI	Conscientiousness	Hispanic	-0.511	< .001
BFI	Neuroticism	Hispanic	0.742	< .001
BFI	Openness	Hispanic	0.274	< .001
Need for Cognition	Need for Cognition	Hispanic	-	-
Need for Closure	Need for Closure	Hispanic	-	-
Systemizing and Emphasizing	Systemizing	Hispanic	-0.340	< .001
Systemizing and Emphasizing	Emphasizing	Hispanic	0.075	.376
Rational-Experiential Inventory	Rational	Hispanic	-	-
Rational-Experiential Inventory	Experiential	Hispanic	-	-
Right-Wing-Authoritarianism	Right-Wing-Authoritarianism	Hispanic	-	-
BFI	Extraversion	Black	-0.024	.996
BFI	Agreeableness	Black	-0.212	.029
BFI	Conscientiousness	Black	-0.456	< .001
BFI	Neuroticism	Black	0.555	< .001
BFI	Openness	Black	0.241	.004
Need for Cognition	Need for Cognition	Black	-	-
Need for Closure	Need for Closure	Black	-	-
Systemizing and Emphasizing	Systemizing	Black	-	-
Systemizing and Emphasizing	Emphasizing	Black	-	-
Rational-Experiential Inventory	Rational	Black	-	-

Continued on next page

Table S14 – continued from previous page

Survey	Construct	Demographic	*d*	*p*
Rational-Experiential Inventory	Experiential	Black	-	-
Right-Wing-Authoritarianism	Right-Wing-Authoritarianism	Black	-	-

Note: *Positive values indicate a higher average response for racial category compared to ChatGPT. Significance determined via Dunnett's Test.*

Figure S13

Comparing ChatGPT against Humans grouped by race for responses on the Big Five personality questionnaire

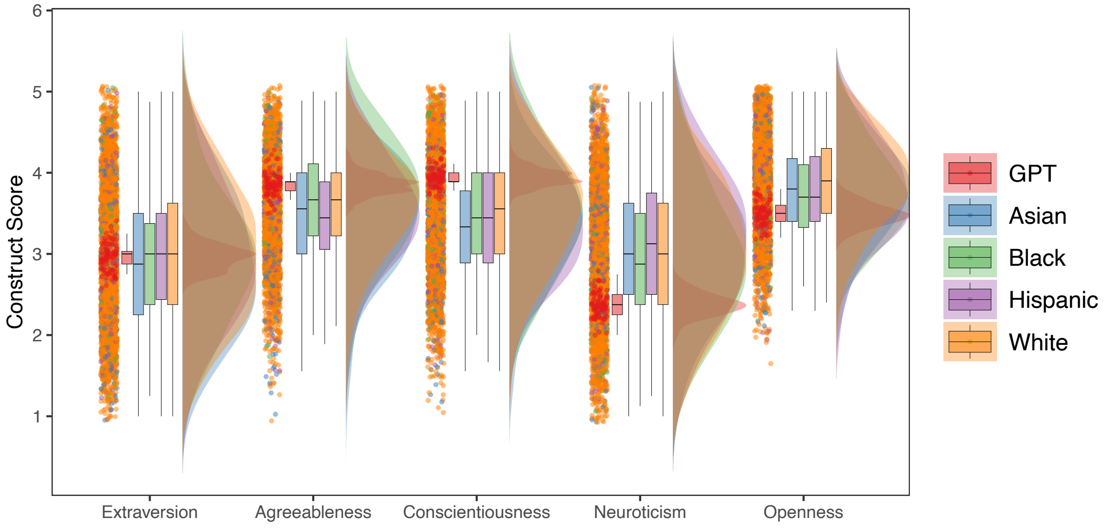

Figure S14

Comparing ChatGPT against Humans grouped by race for responses on the Need for Cognition questionnaire

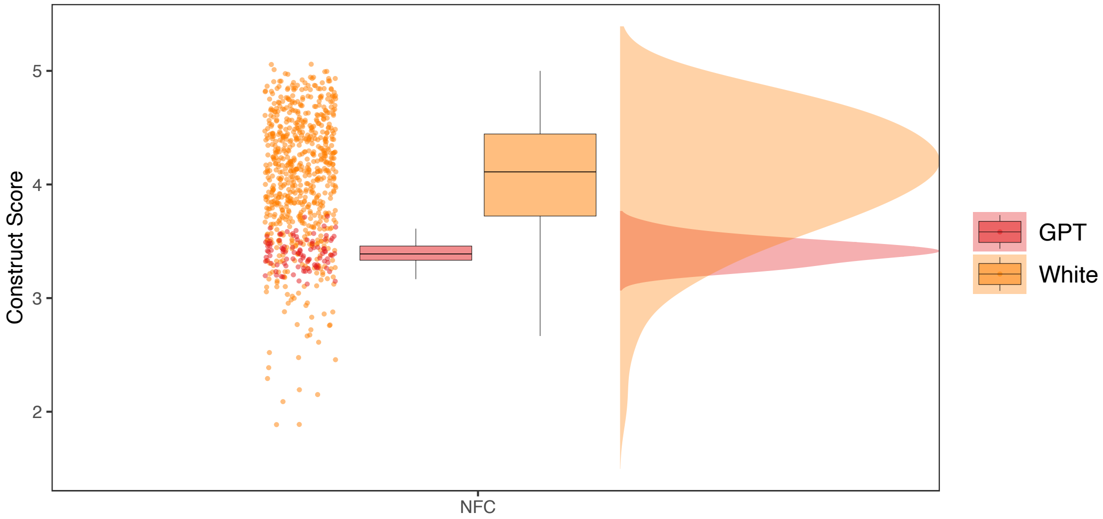

Figure S15

Comparing ChatGPT against Humans grouped by race for responses on the Need for Closure questionnaire

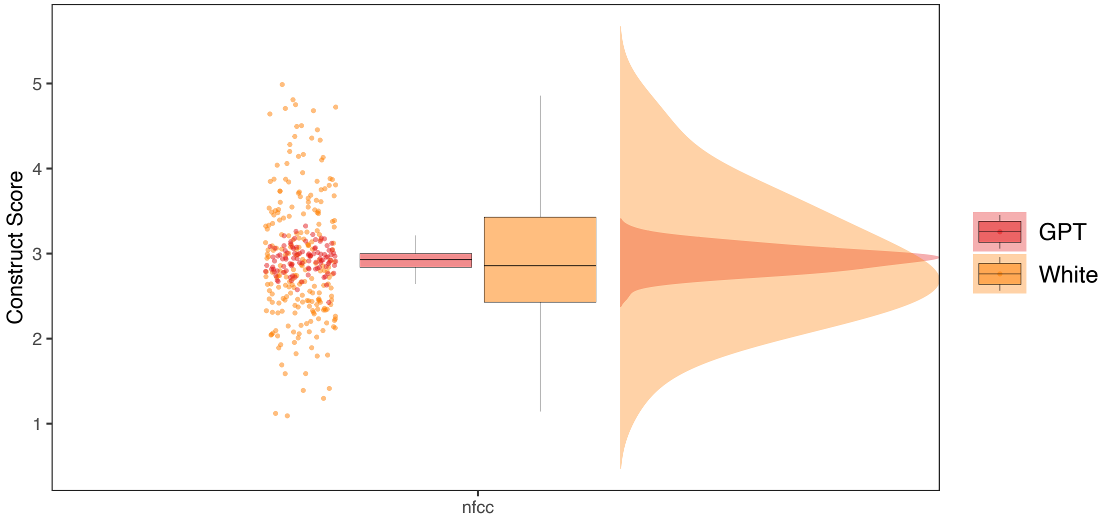

Figure S16

Comparing ChatGPT against Humans grouped by race for responses on the Systematizing-Emphasizing scale

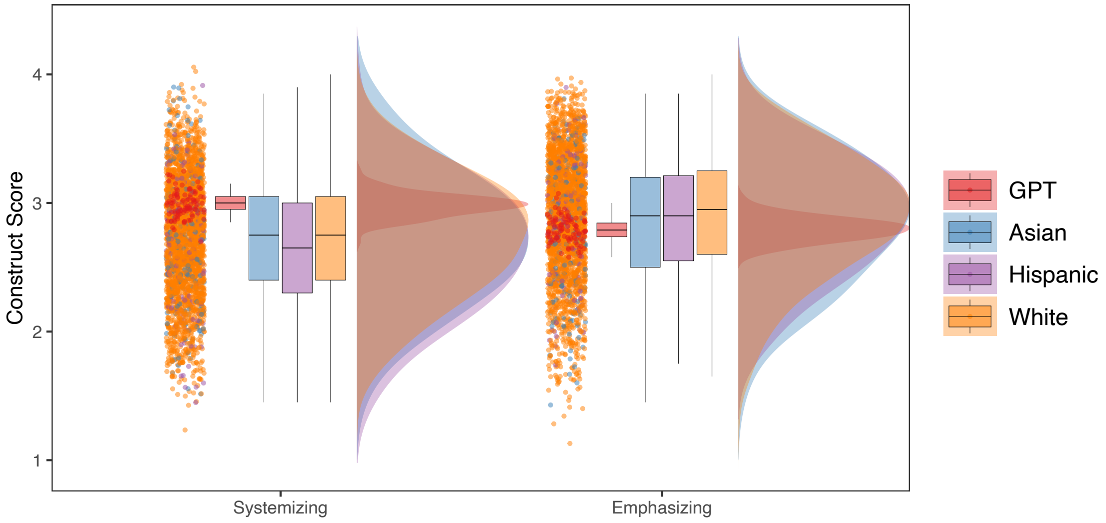

Figure S17

Comparing ChatGPT against Humans grouped by race for responses on the Rational-Experiential Inventory questionnaire

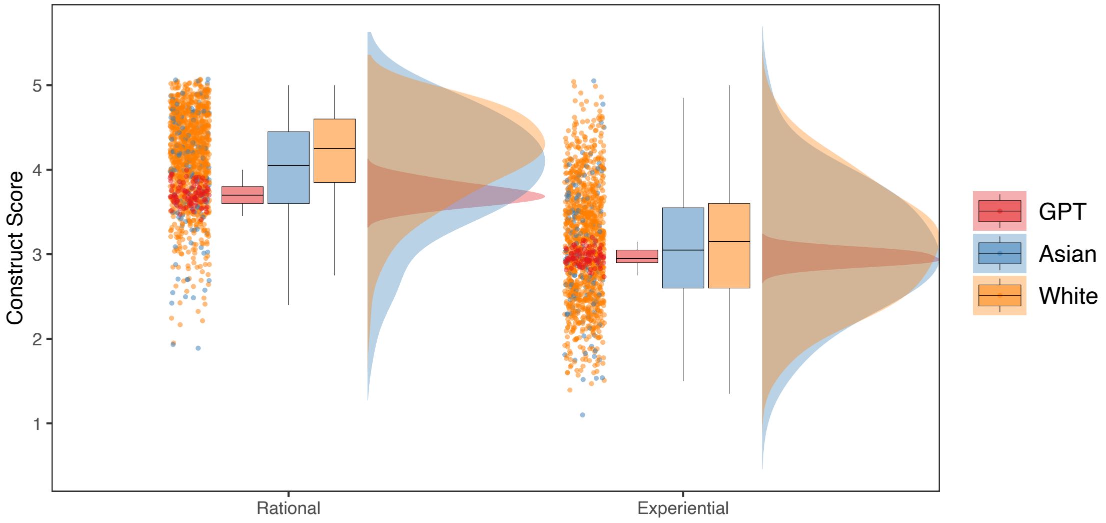

Figure S18

Comparing ChatGPT against Humans grouped by race for responses on the Right-Wing-Authoritarianism questionnaire

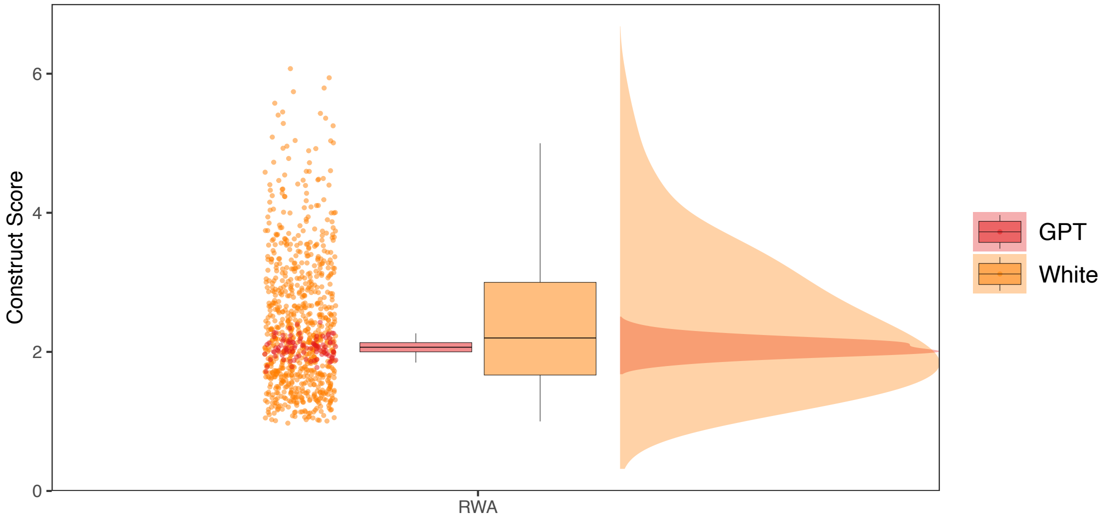

Differences to age groups. We found that ChatGPT significantly deviated from responses of various age groups on most surveys. For example, ChatGPT had significantly lower experiential scores for 18-24 and 35-54 but not for the other age groups or had less neuroticism and open-mindedness while more agreeableness and conscientiousness than all age groups. See Table S16 and Figures S19-S23 for on overview of ChatGPT's deviations from age groups across all surveys.

Table S16

Deviation of ChatGPT responses from human responses grouped by age

**Survey**	**Construct**	**Demographic**	***d***	***p***
BFI	Extraversion	55-74	0.109	.381
BFI	Agreeableness	55-74	-0.180	.021
BFI	Conscientiousness	55-74	-0.217	.009
BFI	Neuroticism	55-74	0.398	$< .001$
BFI	Openness	55-74	0.563	$< .001$
Need for Cognition	Need for Cognition	55-74	0.614	$< .001$
Need for Closure	Need for Closure	55-74	-	-
Systemizing and Emphasizing	Systemizing	55-74	-0.199	$< .001$
Systemizing and Emphasizing	Emphasizing	55-74	0.199	$< .001$
Rational-Experiential Inventory	Rational	55-74	0.452	$< .001$
Rational-Experiential Inventory	Experiential	55-74	0.076	.650
Right-Wing-Authoritarianism	Right-Wing-Authoritarianism	55-74	0.151	.445
BFI	Extraversion	35-54	0.045	.840
BFI	Agreeableness	35-54	-0.263	$< .001$
BFI	Conscientiousness	35-54	-0.364	$< .001$
BFI	Neuroticism	35-54	0.511	$< .001$
BFI	Openness	35-54	0.441	$< .001$
Need for Cognition	Need for Cognition	35-54	0.691	$< .001$
Need for Closure	Need for Closure	35-54	-	-

Continued on next page

Table S16 – continued from previous page

**Survey**	**Construct**	**Demographic**	***d***	***p***
Systemizing and Emphasizing	Systemizing	35-54	-0.180	< .001
Systemizing and Emphasizing	Emphasizing	35-54	0.125	.022
Rational-Experiential Inventory	Rational	35-54	0.505	< .001
Rational-Experiential Inventory	Experiential	35-54	0.178	.042
Right-Wing-Authoritarianism	Right-Wing-Authoritarianism	35-54	0.314	.009
BFI	Extraversion	25-34	-0.040	.884
BFI	Agreeableness	25-34	-0.349	< .001
BFI	Conscientiousness	25-34	-0.516	< .001
BFI	Neuroticism	25-34	0.636	< .001
BFI	Openness	25-34	0.380	< .001
Need for Cognition	Need for Cognition	25-34	0.648	< .001
Need for Closure	Need for Closure	25-34	-	-
Systemizing and Emphasizing	Systemizing	25-34	-0.278	< .001
Systemizing and Emphasizing	Emphasizing	25-34	0.079	.188
Rational-Experiential Inventory	Rational	25-34	0.448	< .001
Rational-Experiential Inventory	Experiential	25-34	0.123	.212
Right-Wing-Authoritarianism	Right-Wing-Authoritarianism	25-34	0.397	< .001
BFI	Extraversion	18-24	0.009	.999
BFI	Agreeableness	18-24	-0.334	< .001
BFI	Conscientiousness	18-24	-0.621	< .001
BFI	Neuroticism	18-24	0.815	< .001
BFI	Openness	18-24	0.183	.005
Need for Cognition	Need for Cognition	18-24	0.465	< .001
Need for Closure	Need for Closure	18-24	-	-
Systemizing and Emphasizing	Systemizing	18-24	-0.393	< .001
Systemizing and Emphasizing	Emphasizing	18-24	0.013	.984

Continued on next page

Table S16 – continued from previous page

**Survey**	**Construct**	**Demographic**	***d***	***p***
Rational-Experiential Inventory	Rational	18-24	0.236	< .001
Rational-Experiential Inventory	Experiential	18-24	0.197	.023
Right-Wing-Authoritarianism	Right-Wing-Authoritarianism	18-24	0.493	< .001

Note: *Positive values indicate a higher average response for demographic groups compared to ChatGPT. Significance determined via Dunnett's Test.*

Figure S19

Comparing ChatGPT against Humans grouped by age for responses on the Big Five personality questionnaire

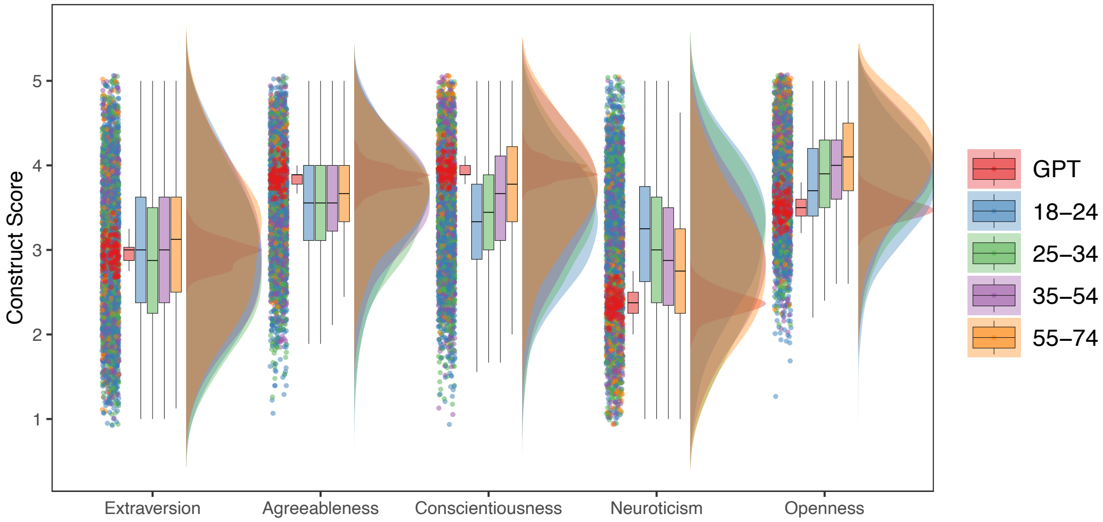

Figure S20

Comparing ChatGPT against Humans grouped by age for responses on the Need for Cognition questionnaire

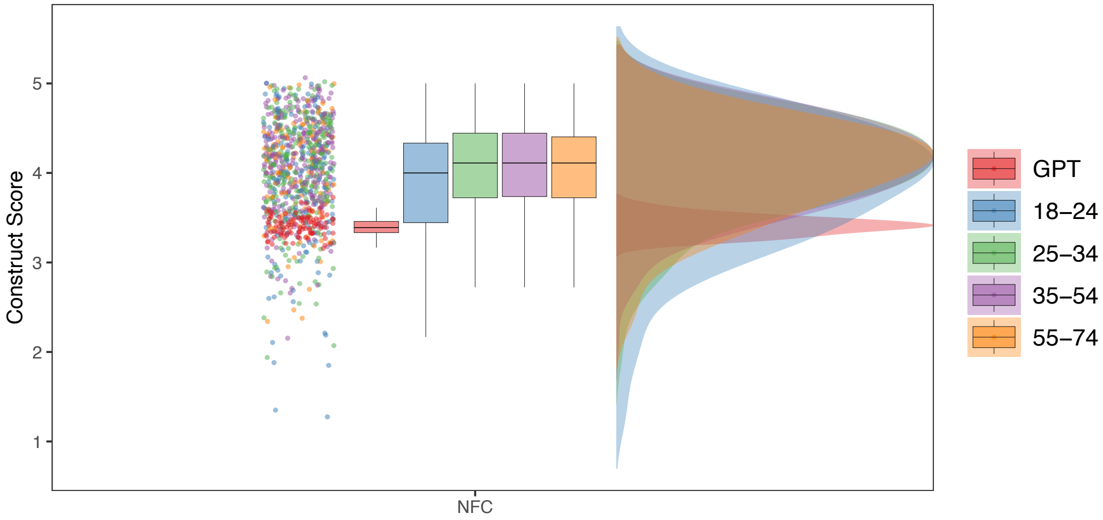

Figure S21

Comparing ChatGPT against Humans grouped by age for responses on the Systematizing-Emphasizing scale

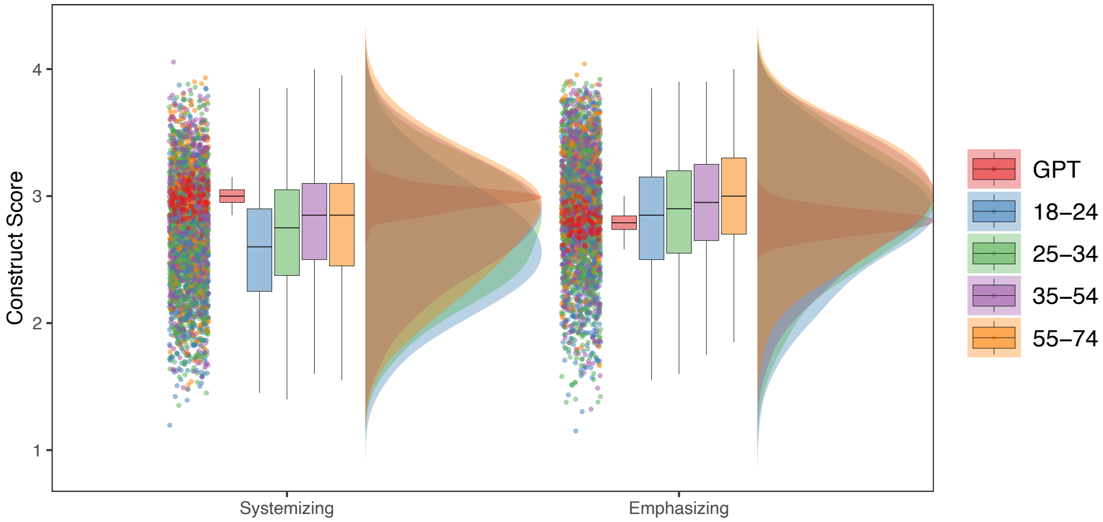

Figure S22

Comparing ChatGPT against Humans grouped by age for responses on the Rational-Experiential Inventory questionnaire

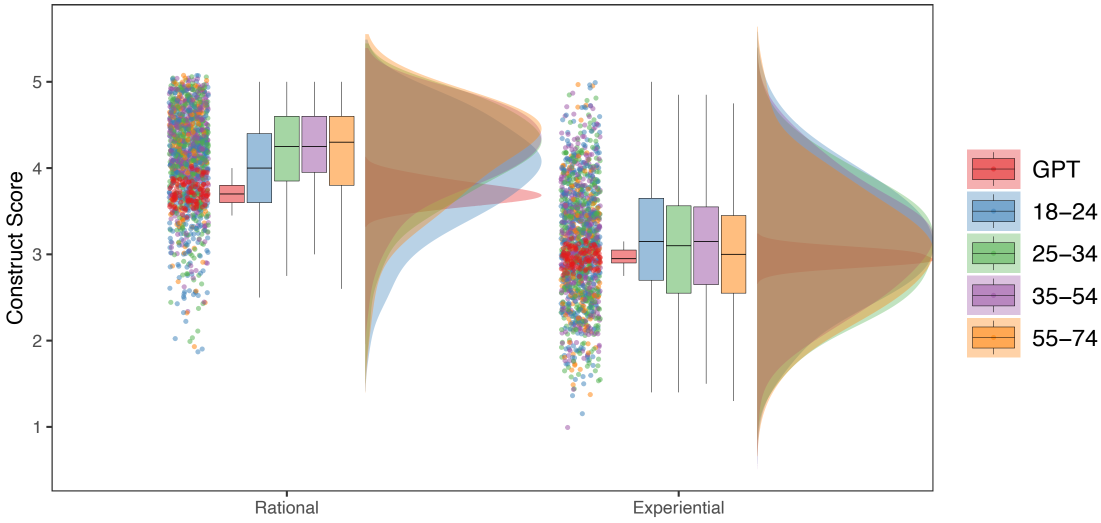

Figure S23

Comparing ChatGPT against Humans grouped by age for responses on the Right-Wing-Authoritarianism questionnaire

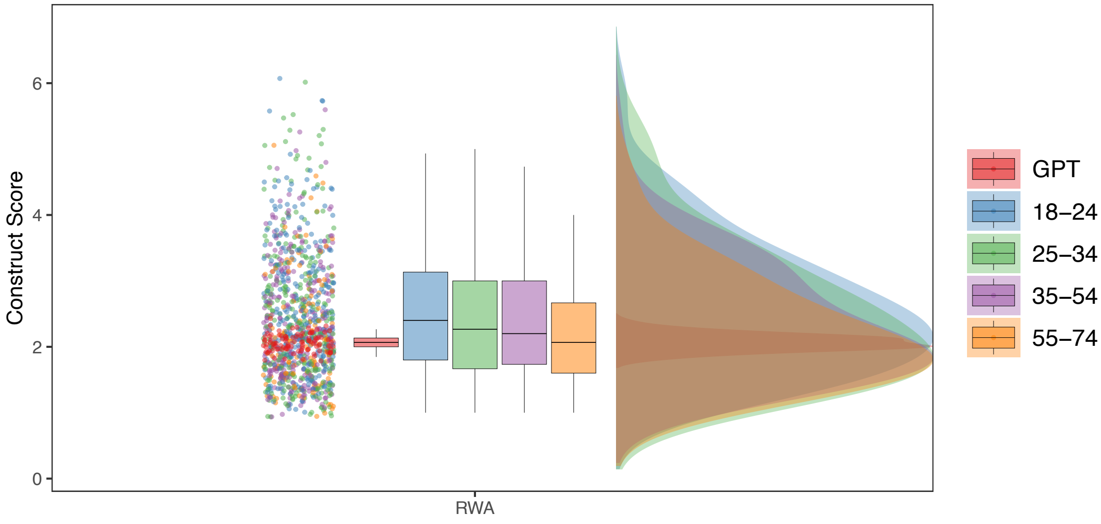

Differences to religious groups. We found that ChatGPT significantly deviated from the responses of various religious groups on most surveys. For example, ChatGPT had lower right-wing-authoritarianism than Christians but higher than Agnostics and Atheists or less neuroticism and open-mindedness while more agreeableness and conscientiousness than all religious groups. See Table S28 and Figures S24-S28 for on overview of ChatGPT's deviations from religious groups across all surveys.

Table S18

Deviation of ChatGPT responses from human responses grouped by religion

Survey	Construct	Demographic	*d*	*p*
BFI	Extraversion	Christianity	0.103	.333
BFI	Agreeableness	Christianity	-0.199	.004
BFI	Conscientiousness	Christianity	-0.381	< .001
BFI	Neuroticism	Christianity	0.589	< .001
BFI	Openness	Christianity	0.271	< .001
Need for Cognition	Need for Cognition	Christianity	0.524	< .001
Need for Closure	Need for Closure	Christianity	-	-
Systemizing and Emphasizing	Systemizing	Christianity	-0.294	< .001
Systemizing and Emphasizing	Emphasizing	Christianity	0.113	.035
Rational-Experiential Inventory	Rational	Christianity	0.347	< .001
Rational-Experiential Inventory	Experiential	Christianity	0.198	.014
Right-Wing-Authoritarianism	Right-Wing-Authoritarianism	Christianity	1.008	< .001
BFI	Extraversion	Atheist	-0.107	.315
BFI	Agreeableness	Atheist	-0.414	< .001
BFI	Conscientiousness	Atheist	-0.532	< .001
BFI	Neuroticism	Atheist	0.640	< .001
BFI	Openness	Atheist	0.427	< .001
Need for Cognition	Need for Cognition	Atheist	0.678	< .001
Need for Closure	Need for Closure	Atheist	-	-

Continued on next page

Table S18 – continued from previous page

**Survey**	**Construct**	**Demographic**	***d***	***p***
Systemizing and Emphasizing	Systemizing	Atheist	-0.215	< .001
Systemizing and Emphasizing	Emphasizing	Atheist	0.039	.581
Rational-Experiential Inventory	Rational	Atheist	0.484	< .001
Rational-Experiential Inventory	Experiential	Atheist	-0.043	.805
Right-Wing-Authoritarianism	Right-Wing-Authoritarianism	Atheist	-0.095	.507
BFI	Extraversion	Agnostic	-0.013	.989
BFI	Agreeableness	Agnostic	-0.356	< .001
BFI	Conscientiousness	Agnostic	-0.560	< .001
BFI	Neuroticism	Agnostic	0.716	< .001
BFI	Openness	Agnostic	0.427	< .001
Need for Cognition	Need for Cognition	Agnostic	0.675	< .001
Need for Closure	Need for Closure	Agnostic	-	-
Systemizing and Emphasizing	Systemizing	Agnostic	-0.268	< .001
Systemizing and Emphasizing	Emphasizing	Agnostic	0.121	.022
Rational-Experiential Inventory	Rational	Agnostic	0.416	< .001
Rational-Experiential Inventory	Experiential	Agnostic	0.217	.006
Right-Wing-Authoritarianism	Right-Wing-Authoritarianism	Agnostic	0.135	.263

Note: *Positive values indicate a higher average response for demographic groups compared to ChatGPT. Significance determined via Dunnett's Test.*

Figure S24

Comparing ChatGPT against Humans grouped by Religion for responses on the Big Five personality questionnaire

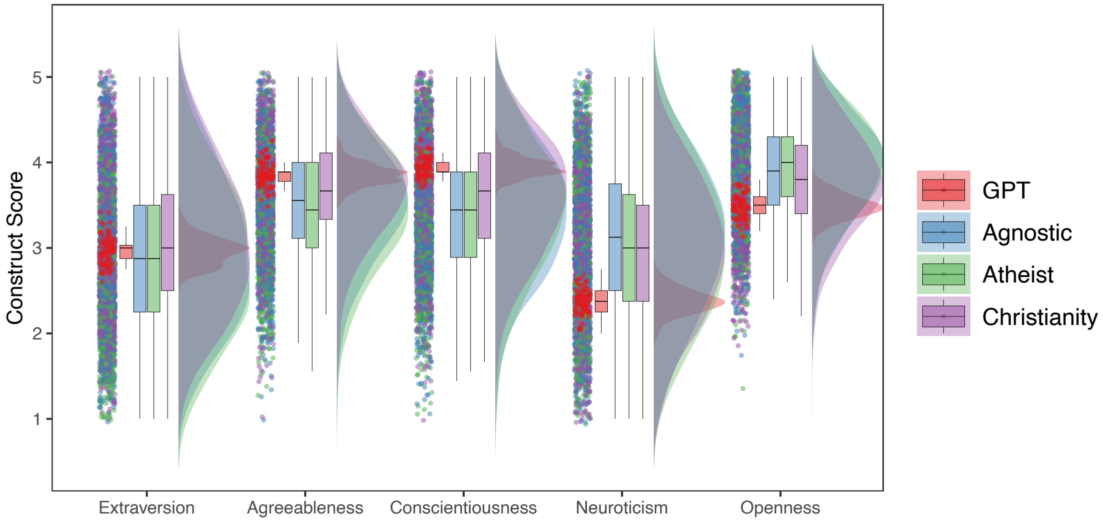

Figure S25

Comparing ChatGPT against Humans grouped by Religion for responses on the Need for Cognition questionnaire

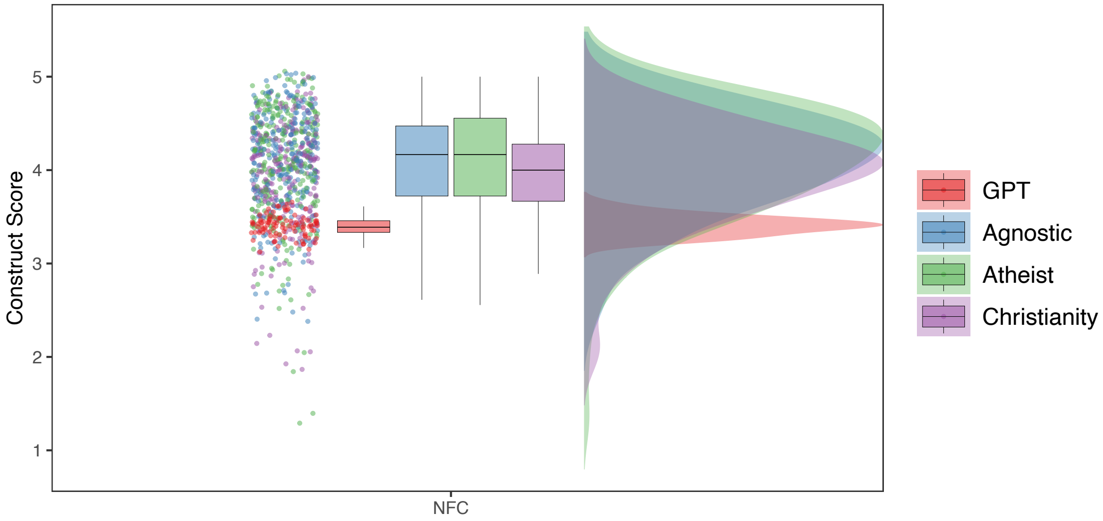

Figure S26

Comparing ChatGPT against Humans grouped by Religion for responses on the Systematizing-Emphasizing scale

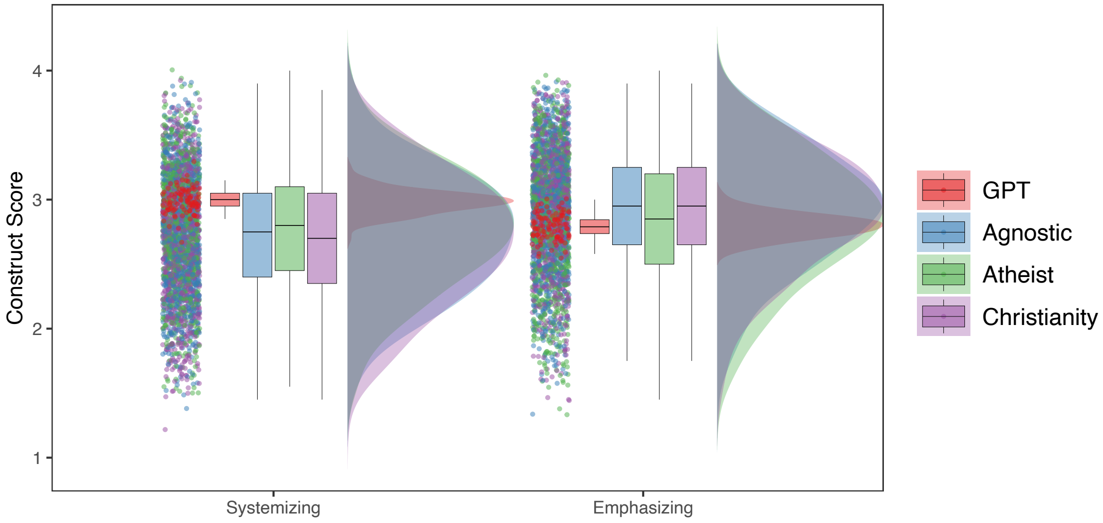

Figure S27

Comparing ChatGPT against Humans grouped by Religion for responses on the Rational-Experiential Inventory questionnaire

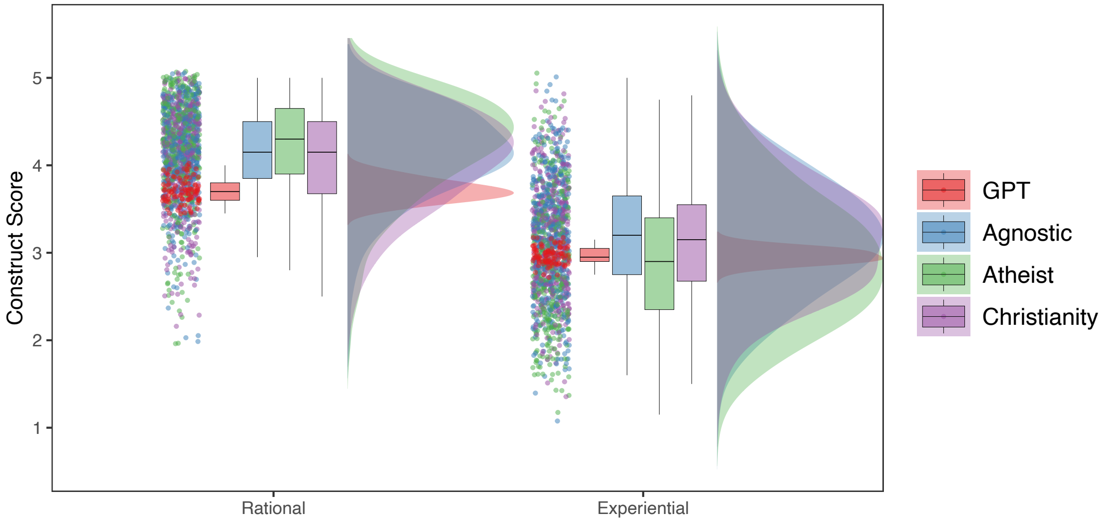

Figure S28

Comparing ChatGPT against Humans grouped by Religion for responses on the Right-Wing-Authoritarianism questionnaire

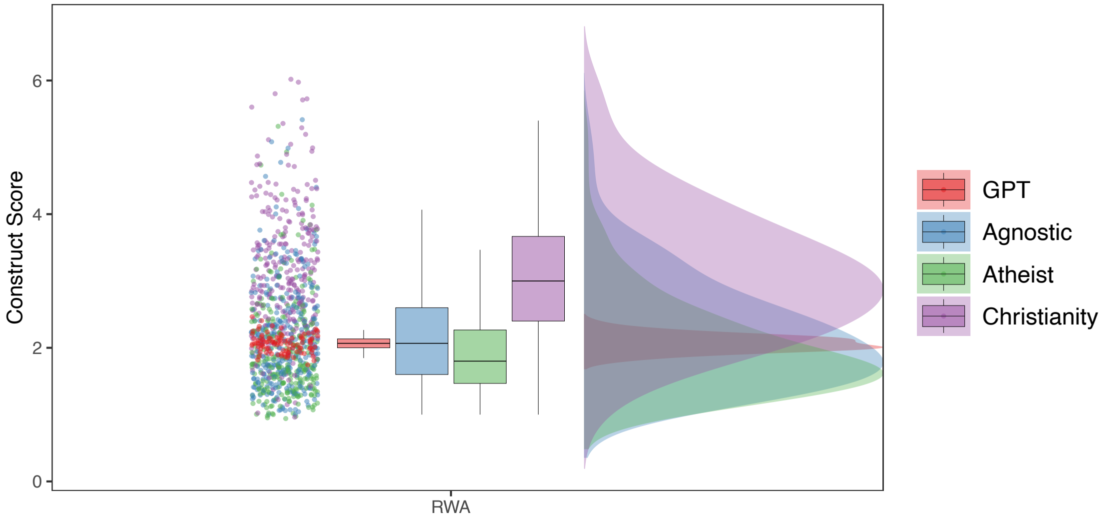

Variance Differences. ChatGPT had significantly less variance in its responses to every survey in comparison to human responses. See Table S20 for an overview of response variance across all surveys.

Table S20

Overview of variance differences between ChatGPT and human responses

Survey	Construct	d	p
BFI	Extraversion	0.68	$< .001$
BFI	Agreeableness	0.51	$< .001$
BFI	Conscientiousness	0.59	$< .001$
BFI	Neuroticism	0.69	$< .001$
BFI	Openness	0.45	$< .001$
Need for Cognition	Need for Cognition	0.47	$< .001$
Need for Closure	Need for Closure	0.62	$< .001$
Systemizing and Emphasizing	Systemizing	0.38	$< .001$
Systemizing and Emphasizing	Emphasizing	0.38	$< .001$
Rational-Experiential Inventory	Rational	0.45	$< .001$
Rational-Experiential Inventory	Experiential	0.61	$< .001$
Right-Wing-Authoritarianism	Right-Wing-Authoritarianism	0.82	$< .001$

Note. Positive values indicate higher variance for humans compared to ChatGPT. Significance determined via Levene-Test. Table shows that this way of prompting is unable to generate human-like variance. See further discussion of meaningfulness of ChatGPT response variance in the main text.

Biases on the BFI. ChatGPT showed significant biases in its BFI responses. In particular, ChatGPT was significantly more similar to females then males, Muslims than Christians, and Christians compared to Jews, Agnostics, and Atheists. See Table S21 for an overview of biases in ChatGPT's BFI responses.

Table S21

Overview of significant ChatGPT biases for the BFI

Bias	Group	Reference Group	b	p
Sex	Male	Female	0.220	$< .001$
Religion	Islam	Christianity	-0.379	.040
Religion	Judaism	Christianity	0.369	.010
Religion	Agnostic	Christianity	0.139	.015
Religion	Atheist	Christianity	0.172	.005

Note. Positive values mean that ChatGPT is biased against a demographic (larger difference to reference group), and negative values mean that ChatGPT is biased toward a demographic (smaller difference). In total, we tested prediction biases for sex, age, political orientation, religion, and race.

RWA ChatGPT biases. ChatGPT showed significant biases in its RWA responses. In particular, ChatGPT was significantly biased against younger people, Conservatives, and Christians compared to their demographic counterparts. See Table S22 for an overview of RWA ChatGPT biases.

Table S22

Overview of significant ChatGPT biases for the RWA

Bias	Group	Reference Group	*b*	*p*
Age	-	0 years	-0.004	$< .001$
Political Orientation	Liberal	Conservative	-1.031	$< .001$
Political Orientation	Moderate	Conservative	-0.876	$< .001$
Religion	Agnostic	Christianity	-0.244	$< .001$
Religion	Atheist	Christianity	-0.223	$< .001$
Religion	Other Religion	Christianity	-0.185	.018

Note. *Positive values mean that ChatGPT predictions are biased against said demographic (larger difference to reference group), and negative values mean that ChatGPT is biased toward said demographic (smaller difference). We tested prediction biases for sex, age, political orientation, religion, and race.*

Biases on the Emphasizing-Systematizing Scale. ChatGPT showed significant biases in its Emphasizing-Systematizing scale responses. In particular, ChatGPT was significantly more similar to older people, males, Conservatives, and Christians compared to their demographic counterparts. See Table S23 for an overview of Emphasizing-Systematizing ChatGPT biases.

Table S23

Overview of significant ChatGPT biases for the Emphasizing-Systemizing Survey

Bias	Group	Reference Group	*b*	*p*
Age	-	0 years	-0.002	$< .001$
Sex	Male	Female	-0.151	$< .001$
Political Orientation	Liberal	Conservative	0.054	.035
Religion	Judaism	Christianity	0.124	.008

Note. Positive values mean that ChatGPT predictions are biased against said demographic (larger difference to reference group), and negative values mean that ChatGPT is biased toward said demographic (smaller difference). We tested prediction biases for sex, age, political orientation, religion, and race.

Biases on the Need for Cognition Scale. ChatGPT showed significant biases in its Need for Cognition scale responses. In particular, ChatGPT was significantly more similar to females, Christians, and Asians compared to their demographic counterparts. See Table S24 for an overview of Need for Cognition ChatGPT biases.

Table S24

Overview of significant ChatGPT biases for the Need for Cognition

Bias	Group	Reference Group	*b*	*p*
Sex	Male	Female	0.083	.005
Religion	Atheist	Christianity	0.097	.014
Race	Asian	White	-0.209	.002

Note. Positive values mean that ChatGPT predictions are biased against said demographic (larger difference to reference group), and negative values mean that ChatGPT is biased toward said demographic (smaller difference). We tested prediction biases for sex, age, political orientation, religion, and race.

Biases on the Need for Closure Scale. ChatGPT showed significant biases in its Need for Closure scale responses. In particular, ChatGPT was significantly more similar to Christians and White people compared to their demographic counterparts. See Table S25 for an overview of Need for Closure ChatGPT biases.

Table S25

Overview of significant ChatGPT biases for the Need for Closure

Bias	Group	Reference Group	*b*	*p*
Religion	Islam	Christianity	1.005	.003
Religion	Atheist	Christianity	0.153	.039
Religion	Other Religion	Christianity	0.229	.037
Race	Asian	White	0.242	.023

Note. *Positive values mean that ChatGPT predictions are biased against said demographic (larger difference to reference group), and negative values mean that ChatGPT is biased toward said demographic (smaller difference). We tested prediction biases for sex, age, political orientation, religion, and race.*

Biases on the Rational-Experiential Scale. ChatGPT showed significant biases in its Rational-Experiential Inventory responses. In particular, ChatGPT was significantly more similar to younger people, females, Moderates, and Christians compared to their demographic counterparts. See Table S26 for an overview of Rational-Experiential Inventory ChatGPT biases.

Table S26

Overview of significant ChatGPT biases for the Rational-Experiential Inventory

Bias	Group	Reference Group	*b*	*p*
Age	-	0 years	0.004	$< .001$
Sex	Male	Female	0.190	$< .001$
Political Orientation	Moderate	Conservative	-0.144	.020
Religion	Hinduism	Christianity	0.405	.048
Religion	Atheist	Christianity	0.215	$< .001$
Religion	Other Religion	Christianity	0.183	.019

Note. Positive values mean that ChatGPT predictions are biased against said demographic (larger difference to reference group), and negative values mean that ChatGPT is biased toward said demographic (smaller difference). We tested prediction biases for sex, age, political orientation, religion, and race.

S3. Prompt Sensitivity

We investigated ChatGPT's sensitivity to changes in prompts when generating psychologically relevant outputs. To that end, we repeated the previous two studies on moral sentiment analysis and survey response collection using ChatGPT while modifying the prompts.

Methods

Data. To test ChatGPT's sensitivity to prompts during sentiment analysis, we utilized the same randomly selected subset of the MFRC (Trager et al., 2022b) as in the original study (2,983 samples). To test ChatGPT's sensitivity to prompts when responding to psychological questionnaires, we utilized the BFI (Fossati et al., 2011) questionnaire. We make all data, study materials, and analysis code available at https://osf.io/nafzy/. The repository contains all the necessary instructions to replicate our analyses.

Text Analysis. We prompted ChatGPT to annotate posts from the MFRC test data via the OpenAI API (https://platform.openai.com/docs/api-reference?lang=python). To access the API and collect ChatGPT's text annotations, we used the OpenAI library (v0.27.8; OpenAI (2023)) in Python. We deployed the "gpt-3.5-turbo-0301" model with a temperature of 0 (for maximal deterministic and thus replicable behavior). In the original study, we prompted ChatGPT in a way analogous to how human annotators are instructed. That is, we described the task of identifying moral sentiment in a text and gave it the definitions of each type of moral sentiment. To test ChatGPT's sensitivity to prompt changes, we modified the prompt systematically, by randomly changing the order in which the definitions of the moral foundations are given to the model. Importantly, we did not change the amount or type of information given to ChatGPT. We also kept sentence structure and word choice constant. The following prompt was applied: "Determine which moral sentiments are expressed in the following text. "purity if the text is about avoiding bodily and spiritual contamination and degradation, "equality if the text is about equal treatment and equal outcome for individuals, "authority if the text is about deference toward legitimate authorities and the defense of traditions, all of which are seen as providing stability and fending off chaos, "thin morality if the text has a moral sentiment but cannot be categorized as either of the above, "loyalty if the text is about cooperating with

ingroups and competing with outgroups, "proportionality if the text is about individuals getting rewarded in proportion to their merit or contribution, "non-moral" if no moral sentiment is expressed in the text, "care if the text is about avoiding emotional and physical damage to another individual. Respond only with these words. Respond with any of the categories that apply, comma separated. Here is the text: [Text to annotate]"

Survey responses. We prompted ChatGPT to respond to survey items via the OpenAI API (https://platform.openai.com/docs/api-reference?lang=python). To access the API and to collect the responses we used the OpenAI library (v0.27.8; OpenAI (2023)) in Python. We deployed the "gpt-3.5-turbo-0301" model with a temperature of 1 (to allow for maximal variability in the responses). The prompts mirrored the study instructions given to human respondents and had the general shape of "For a scientific study, please indicate your level of agreement with the following statement. Use integer numbers from 1 to 5, with 1 meaning strongly disagree and 5 meaning strongly agree. Respond with a single number. The statement is: [Survey item]"

Based on Schwarz (1999), who showed that minor changes in self-report design such as contextually relevant information or even simply the number values of the response scale can change response patterns, we developed two alternative prompts. The first prompt added a study introduction, similar to what human participants may see in a study. The rationale was to give the model context information when responding to the items (e.g., that this is for a study, that these items are measuring personality, etc.). The prompt was as follows: "For a scientific study, we are collecting responses to various psychological questionnaires. In the following, you will be shown statements from a personality test. Please indicate your level of agreement with each statement. There are no right or wrong answers. Use numbers from 1 to 5, with 1 meaning strongly disagree and 5 meaning strongly agree. Respond with a single number. The statement is: [Survey item]"

The second prompt changed the response scale numerical values from 1–5 to -2 – 2. The total range of the scale, center, and meaning of each point remained the same. We were interested in whether a simple change in the numbers presented, with no changes in meaning and information content, would lead to a change in outputs. The prompt was as follows: "For a scientific study, please indicate your level of agreement with the following statement. Use integer

numbers from -2 to 2, with -2 meaning strongly disagree and 2 meaning strongly agree. Respond with a single number. The statement is: [Survey item]"

We then created an additional prompt that had only surface-level semantic changes. Keeping the sentence structure and meaning constant, we exchanged words with synonyms or analogous idioms. The rationale was to test if ChatGPT's output was responsive to minor, meaningless changes in the instructions. The prompt was as follows: "For an ongoing research project, please express how much you agree with the statement shown at the end. Respond with an integer between 1 and 5, with 1 indicating a strong disagreement and 5 indicating a strong agreement. Respond only with a single digit. Here's the statement: [Survey item]"

We collected 100 responses for each item. That is, we prompted ChatGPT to respond to the same question 100 times to allow for analyses regarding the variance within ChatGPT responses. See the project repository for the utilized survey items, ChatGPT prompts, and code containing the API calls.

Sensitivity Analysis. We tested whether changes in a prompt lead to significant changes in the generated outputs. For the sentiment analysis task, we tested whether the modified prompt led to changes in sentiment classification across all types of moral sentiment. Specifically, we tested whether each of the moral sentiments is more or less likely to be classified as present in a text given the respective prompt. We fit a logistic regression model predicting whether a moral sentiment was classified as present in a text (binary: yes/no) as a function of the prompt (original vs. modified). The coefficients of this model expressed how much more or less likely a moral sentiment was for the modified prompt. A significant coefficient, thus, expressed a significant change in output in response to the prompt changes.

For the survey responses, we tested whether the different prompts led to significant differences in the survey responses. Specifically, we tested across all survey constructs (e.g., Openness, Conscientiousness, Extraversion, Agreeableness, Neuroticism) whether the prompts led to different construct scores compared to the original prompt. We tested these differences for statistical significance using Dunnett's test (Dunnett, 1955), which compared each of the alternative prompts (ALT1: adding context information, ALT2: changing the scale values, ALT3: rephrasing using synonyms) against the original prompt, while accounting for multiple

comparisons. Finally, we tested whether the prompt changes also impacted the variance in survey responses. We were particularly interested in whether prompt changes would lead to increased variance, indicating more diversity in responses.

We conducted all statistical analyses in R (v4.1.2; R Core Team (2021)) using the stats (v4.1.2; R Core Team (2021)) package (R Core Team, 2021). See the code repository for instructions to replicate the statistical analyses.

Results

Prompt Sensitivity in Text Annotations. We found that changing the prompt order did not meaningfully change annotation accuracy ($F1 = 0.23$ vs $F1 = 0.22$), but lead to significant changes in the classification of each texts' moral sentiment. Using the modified prompt, ChatGPT was significantly less likely to annotate the care foundation (-56%, $p < .001$), the equality foundation (-62%, $p < .001$), the loyalty foundation (-33%, $p = .009$), and significantly more likely to annotate the authority foundation (+71%, $p < .001$), purity foundation (+164%, $p = .02$), or thin-morality (+54%, $p < .001$). Table S27 shows all differences in the odds of classifying a text as containing a moral sentiment for the modified vs. original prompt.

Table S27

Deviation of modified vs. original prompt in classifying prediction class

Class	$\Delta_{log\ odss}$	p value
Care	-56%	$<$ .001
Equality	-62%	$<$ .001
Proportionality	-31%	.09
Loyalty	-33%	.01
Authority	+71%	$<$ .001
Purity	+164%	.02
Thin-Morality	+54%	$<$ .001
Non-moral	-100%	.95

Note. *Table shows the difference in odds for ChatGPT to predict a class using the modified prompt compared to the original prompt. The table shows that the prompt changes led to significant differences in classifying moral sentiment.*

Prompt Sensitivity in Survey Responses. We found that changes to the response scale values, adding additional contextual information in the form of an introduction, and surface-level semantic changes led to significant changes in ChatGPT responses to surveys. Table S28 shows the differences across constructs of the BFI for each of the modified prompts compared to the original prompt. However, there were no changes in variance in the responses for any of the prompts (Bonferroni corrected p of .0025) as shown in Table S29, again highlighting that the issues with producing meaningful response variance might be a characteristic of LLMs robust to these simple prompting strategies.

Figure S29

Differences in responses across prompts with surface-level differences

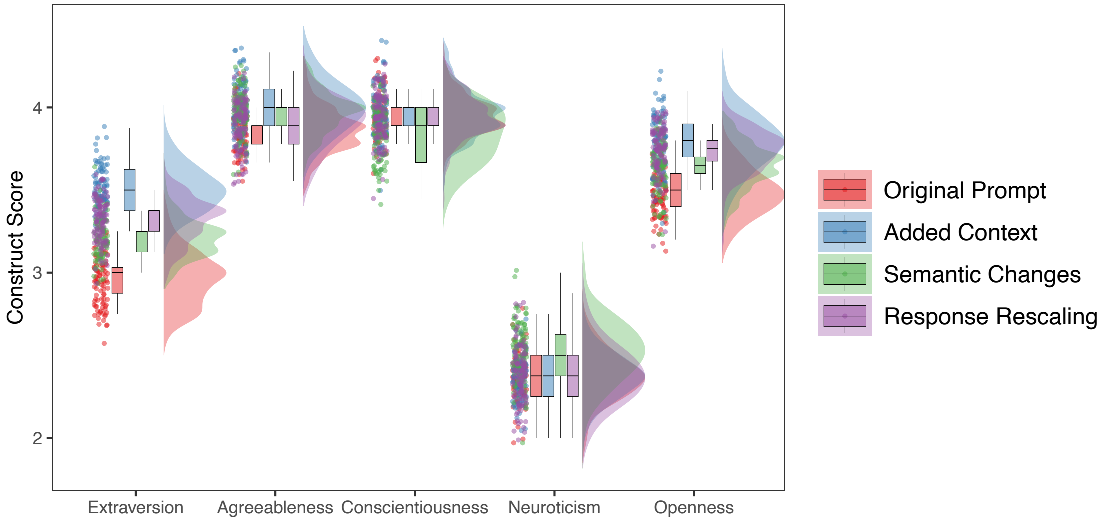

Note. Figure shows the effect of surface-level changes to the prompt on ChatGPT's output. In most cases the changes led to significant changes in output.

Our results highlight how minor changes in prompt design can lead to significant changes in output generated by LLMs for scientific purposes. Notably, we found changes in response averages but not in variance, indicating LLMs' difficulty in generating diverse responses. This is relevant as it goes against the notion that LLMs are an unproblematic off-the-shelf text analysis tool or even a replacement for human study participants and instead highlights the need for future work that develops methods for the robust integration of LLMs in psychology research. Of

Table S28

Differences in ChatGPT responses on the BFI for modified vs. original prompt

Construct	Prompt Modification	d	p
Extraversion	Adds Introduction	0.54	$< .001$
Agreeableness	Adds Introduction	0.14	$< .001$
Conscientiousness	Adds Introduction	0.01	.927
Neuroticism	Adds Introduction	-0.00	1.000
Openness	Adds Introduction	0.37	$< .001$
Extraversion	Synonyms	0.25	$< .001$
Agreeableness	Synonyms	0.11	$< .001$
Conscientiousness	Synonyms	-0.11	$< .001$
Neuroticism	Synonyms	0.15	$< .001$
Openness	Synonyms	0.16	$< .001$
Extraversion	Response scale value	0.36	$< .001$
Agreeableness	Response scale value	0.04	.057
Conscientiousness	Response scale value	-0.01	.986
Neuroticism	Response scale value	0.01	.992
Openness	Response scale value	0.25	$< .001$

Note. *Positive values indicate a higher average response when using the original prompt compared to the modified prompts. Significance determined via Dunnett's Test. Table shows that most prompt modifications lead to significant changes in the generated responses.*

Table S29

Differences in ChatGPT response variance on the BFI for modified vs. original prompt

Construct	Prompt Modification	*d*	*p*
Extraversion	Adds Introduction	0.03	.033
Agreeableness	Adds Introduction	-0.02	.435
Conscientiousness	Adds Introduction	-0.03	.169
Neuroticism	Adds Introduction	-0.01	.646
Openness	Adds Introduction	-0.03	.096
Extraversion	Synonyms	0.05	.010
Agreeableness	Synonyms	0.01	.385
Conscientiousness	Synonyms	-0.05	.002
Neuroticism	Synonyms	-0.03	.022
Openness	Synonyms	-0.00	.359
Extraversion	Response scale value	0.03	.132
Agreeableness	Response scale value	-0.01	.389
Conscientiousness	Response scale value	-0.02	.308
Neuroticism	Response scale value	-0.03	.046
Openness	Response scale value	-0.00	.571

Note. *Positive values indicate higher variance in response values when using the original prompt compared to the modified prompts. Significance determined via Levene-Test.*

course, future work can and will investigate how prompting can be used to generate more reliable and diverse results. However, researchers should be aware of the increased complexity this means for scientific studies (e.g., needing to validate prompts against alternatives) and the current issues that we have showcased.

S4.Theory-driven vs. Bottom-up Methods

We compared ChatGPT as a bottom-up, difficult-to-interpret method against Contextualized Construct Representation (CCR; Atari, Omrani, & Dehghani, 2023b), a top-down, theory-based, easier-to-interpret method. CCR is a theory-driven text analysis method that combines the benefits of contextual language models (e.g., BERT's powerful and flexible semantic representations) with the interpretability and validity of common psychometric scales. In essence, CCR imposes top-down constraints on an embedding model (based on comparing semantic similarities of texts to psychometric scale items), thus achieving higher performance while maintaining interpretability. Our main research question is whether LLMs, specifically ChatGPT, can at this stage of their development already outperform validated top-down methods. This is relevant because, usually, bottom-up methods are utilized because they are more powerful (e.g., higher predictive accuracy) and flexible (e.g., can be used on a wider range of data and tasks) than their theory-based counterparts. This increase in performance, however, is bought with reduced interpretability (e.g., how do text features lead to a prediction in an LLM? What text features were used?). Note that while CCR does incorporate BERT-based embeddings (which is a language model, albeit significantly smaller and more lightweight compared to GPT3.5), it does so with strong theoretical top-down constraints, thus allowing it to provide some level of interpretability. If LLM-based methods cannot outperform these constrained top-down methods, it should be argued that they are not advanced and reliable enough for widespread usage in psychology and related research fields. Of course, this can change in the very near future. However, researchers should be cautious of using these methods before validating their abilities and comparing them to alternative approaches.

Methods

Data. We replicated the analysis in Atari, Omrani, and Dehghani (2023b) that introduced CCR as a method. In this paper, the authors test CCR's capabilities by using it to infer participants' responses to a wide range of questionnaires, including the Portrait Values Questionnaire (PVQ-21; S. H. Schwartz (2003)), Moral Foundations Questionnaire (MFQ-2; Atari, Haidt, et al. (2023a)), Cultural Tightness-Looseness (Oyserman, 1993), Collectivism

(Oyserman, 1993), Need for Cognition (NFC; Cacioppo and Petty (1982b)), as well as self-report ratings on religiosity (Abdel-Khalek, 2007; Afhami et al., 2017) and political orientation (liberal vs. conservative). The predictions were made based on two types of essays that the participants wrote—one about their personal values and one about their everyday life and behaviors. We utilized the same essays to infer participant responses about the different constructs (22 in total) in the original data set.

CCR Predictions. We use the CCR analog to the original paper (Atari, Omrani, & Dehghani, 2023b). CCR creates similarity scores between the participant's essay and the text of each item that was used to measure a participant's response for a respective construct. Then, a linear regression model is fit for each construct predicting the participants' construct scores based on these similarity scores. Finally, the regression model's R^2 score is reported. These scores are then used to compare CCR and ChatGPT performance across all constructs.

ChatGPT Predictions. We prompted ChatGPT to respond to survey items via the OpenAI API (https://platform.openai.com/docs/api-reference?lang=python). To access the API and to collect the responses we used the OpenAI library (v0.27.8; OpenAI (2023)) in Python. We deployed the "gpt-3.5-turbo-0301" model with a temperature of 0 (to allow for maximal reproducibility). We utilized two types of prompts. First, we prompted ChatGPT to predict a participant's rating on a psychological construct (e.g., conservatism) based on one of the essays they wrote. This prompt was supposed to test the model's general ability to make these kinds of predictions without any further information. The prompt was as follows: "For a scientific study, rate how strongly the author of the following text endorses "Individualism" solely based on the text they have written. Here is the text: [TEXT TO INTERPRET]

Respond with a single number of up to two decimal points and between 1 and 5, with 1 meaning "strongly disagree" and 5 meaning "strongly agree". Respond only with this single number and nothing else. Do not use words."

The second prompt was designed to be more similar to CCR's method, in that it included the item texts that were used to measure the respective participant responses. Specifically, we prompted ChatGPT to predict the responses to the respective items, instead of the construct directly. This could potentially improve performance as the items provide context about what the

construct in question means. The prompt was as follows: "For a scientific study, rate how strongly the author of the following text endorses the following psychological items solely based on the text they have written. Here is the text: [TEXT TO INTERPRET]

Here are the items: [ITEMS TO INFER]

Respond to each item with a single digit between 1 and 5, with 1 meaning strongly disagree and 5 meaning strongly agree. Respond with exactly [NUMBER OF ITEMS] numbers, comma separated. Do not use words."

We adapted the scale descriptions to the surveys and constructs as necessary. We collected a separate response for each essay type (that is, we repeated the same prompt twice). See the project repository for the utilized survey items, ChatGPT prompts, and code containing the API calls.

Performance Evaluation. We fit one linear regression model for each construct predicting the participants' scores based on ChatGPT's ratings. We fit these models twice, once based on ChatGPT's ratings on the construct level and once based on the item level. We then report the R^2 scores of each model (construct-level and item-level) for all constructs. These R^2 scores were then compared for statistical significance against CCR's R^2 scores using Dunnett's Test, which tests the two ChatGPT models against the CCR model while accounting for multiple group comparisons. We report the model differences, including p-value and 95% confidence interval. Lastly, we test whether the ChatGPT performance and CCR performance are correlated, which indicates whether constructs are similarly captured by either method (i.e., constructs that are more difficult to capture for one method are also more difficult for the other). We conduct this analysis once for ratings based on values essays, that is, CCR and ChatGPT predictions based on participants' essays about their individual values, and once for ratings based on participants' essays about their everyday life.

We conducted all analyses in R (v4.1.2; R Core Team (2021)) using the stats (v4.1.2; R Core Team (2021)), car (v3.0-11; Fox and Weisberg (2019)), and DescTools (v0.99.44; Andri et mult. al. (2021)) packages.

Results

We found no difference between ChatGPT-based ratings and CCR across all tested constructs except for one, where CCR outperformed construct-level ChatGPT predictions in everyday essays. Specifically, in the value essays (Figure S30) CCR performed on par with ChatGPT ratings on the construct-level (Dunnett's Test; $d = -0.791$, $p = .798$, 95% *CI* [-3.975, 2.392]) and on the item-level (Dunnett's Test; $d = 2.211$, $p = .209$, 95% *CI* [-0.972, 5.394]). In the everyday essay (Figure S31) CCR outperformed ChatGPT ratings on the construct-level ((Dunnett's Test; $d = -2.253$, $p = .005$, 95% *CI* [-3.881, -0.624]) and performed on par with ChatGPT's ratings on the item-level (Dunnett's Test; $d = 0.209$, $p = .940$, 95% *CI* [-1.419, 1.837]). See Tables S30 and S32 for an overview of model performances across all self-report measures. Lastly, ChatGPT's performance correlated highly with CCR across all measures for both essays. Specifically, on the value essays, we found a strong relationship between ChatGPT's performance when using item-level ratings and CCR ($r = .81$, $df = 20$, $p < .001$), as well as when using construct-level ratings ($r = .70$, $df = 20$, $p < .001$). On the everyday essays, we found a strong relationship between ChatGPT's performance when using construct-level ratings and CCR ($r = .65$, $df = 20$, $p = .001$), but not when using item-level ratings ($r = .40$, $df = 20$, $p = .062$).

Overall, we found that ChatGPT could not outperform the less complex, theory-based, method. Furthermore, the high correlation of performances indicates that ChatGPT does not possess capabilities that go notably beyond the more constrained theory-based method (that is, where ChatGPT performs better, CCR also performs better). Lastly, but importantly, the performance differences between ChatGPT when giving ratings on psychological items, instead of constructs or general concepts, show that even LLMs can benefit from the integration of theory-based methodologies and frameworks. Extending this line of work, and developing comprehensive methods that can combine the benefits of both worlds, is a promising endeavor for future work. Again, our intention here is not to posit that LLMs cannot be used in psychology research, be it as a tool or as the focus of a research question. Instead, we aim to show where these methods currently struggle, and how they compare against other established, more interpretable alternatives. The choice of methodology, ultimately, lies with the researcher and should be made after careful consideration of all relevant concerns—and advantages.

Table S30

Model performances (R^2 in %) on the value essays

Measures	CCR	ChatGPT (construct-level)	ChatGPT (item-level)
Individualism	4.58	4.02	12.10
Collectivism	3.70	1.50	3.87
Religiosity	13.30	13.64	28.83
Tightness	5.10	8.07	1.60
Conservatism	10.19	2.55	5.36
Care	6.23	3.12	3.83
Equality	1.31	2.38	1.33
Proportionality	3.10	9.30	5.66
Loyalty	4.81	3.85	5.79
Authority	2.59	7.00	10.34
Purity	9.65	6.58	17.04

Table S31

Model performances (R^2 in %) on the value essays

Measures	CCR	ChatGPT (construct-level)	ChatGPT (item-level)
Need for Cognition	1.01	0.10	1.51
Achievement	2.62	2.87	2.79
Benevolence	0.84	1.76	2.07
Conformity	0.59	0.70	2.28
Hedonism	0.76	3.17	2.31
Power	0.37	0.00	1.64
Security	0.24	0.73	2.13
Self-Direction	1.25	4.48	2.29
Stimulation	2.98	0.17	3.41
Tradition	0.35	0.00	3.00
Universalism	3.36	1.12	3.81

Table S32

Model performances (R^2 in %) on the everyday essays

Measures	CCR	ChatGPT (construct-level)	ChatGPT (item-level)
Individualism	3.07	0.02	4.22
Collectivism	3.58	1.02	2.67
Religiosity	8.94	2.58	10.63
Tightness	2.61	0.01	1.88
Conservatism	6.15	0.10	0.24
Care	4.02	0.02	3.42
Equality	1.63	0.09	2.22
Proportionality	2.71	0.69	2.62
Loyalty	1.15	0.05	1.24
Authority	2.69	0.33	4.27
Purity	12.7	1.75	3.45

Table S33

Model performances (R^2 in %) on the everyday essays

Measures	CCR	ChatGPT (construct-level)	ChatGPT (item-level)
Need for Cognition	3.00	8.66	0.45
Achievement	1.19	0.59	1.53
Benevolence	1.02	0.04	3.67
Conformity	0.14	0.15	0.81
Hedonism	0.74	0.42	0.92
Power	0.28	0.04	1.88
Security	0.16	0.26	0.35
Self-Direction	0.09	0.85	0.93
Stimulation	0.85	0.17	3.15
Tradition	2.08	0.55	6.74
Universalism	3.36	0.97	0.51

Figure S30

Comparing explained variance for CCR, GPT at the construct-level, and GPT at the item-level scores on the values essay

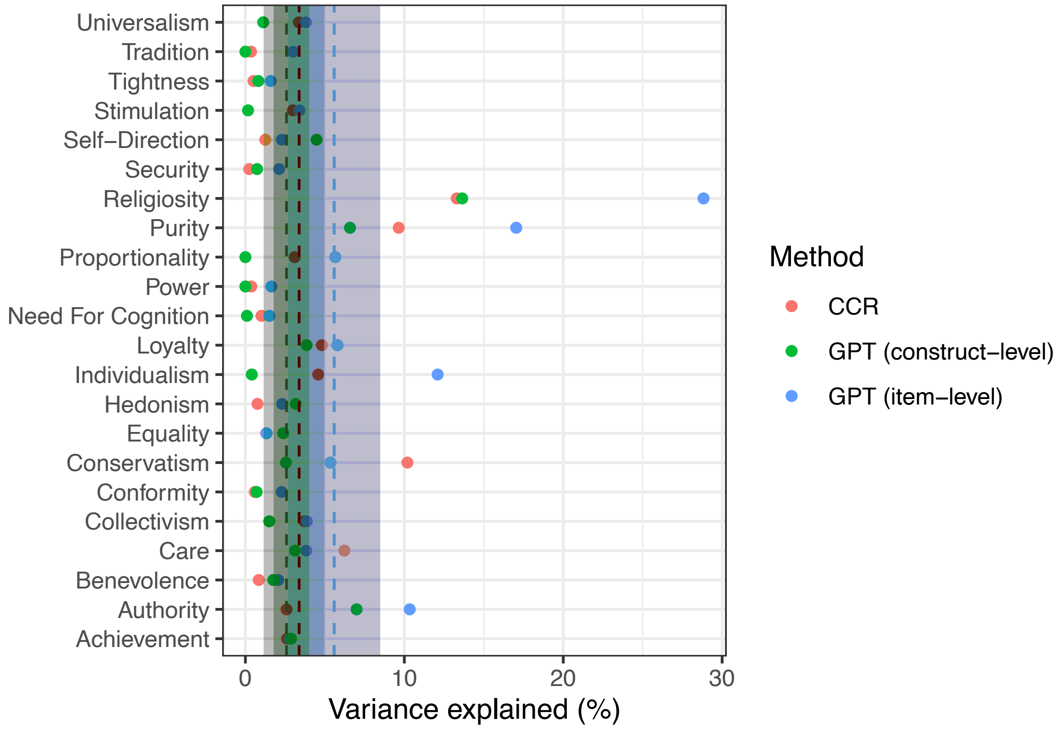

Note. *Shaded area indicates 95% CI for the average variance explained for each prediction method. Figure shows that neither classification method significantly differs from each other.*

Figure S31

Comparing explained variance for CCR, GPT at the construct-level, and GPT at the item-level scores on everyday essays

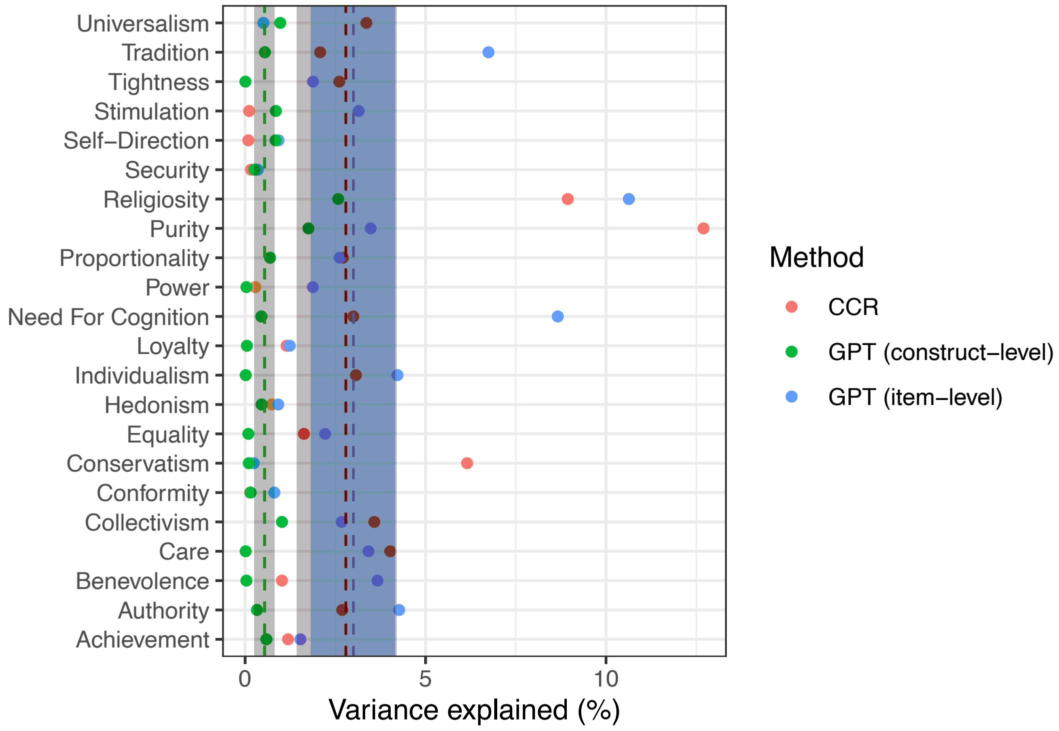

Note. Shaded area indicates 95% CI for the average variance explained for each prediction method. Figure shows that GPT predictions on the construct-level (green) explain significantly variance than CCR (red) and GPT predictions on the item-level (blue)

S5. Open vs. Closed Language Models

To strengthen our arguments for using open-source LLMs over closed proprietary LLMs, we repeated our main analyses using an open-source model. Specifically, we repeat our comparison of LLMs vs. fine-tuned BERT-based models and the bias analysis towards annotator demographics. We then repeat our collection of survey response data on the same range of questionnaires. We finally investigate whether the open-source LLM's output is comparable to that of ChatGPT. Overall, we show that we can generate comparable performance/data distribution to ChatGPT using a locally run open-source LLM on strictly consumer-grade computer hardware.

Specifically, we used a model based on the second generation of Meta's open-source LLM ("LLaMA"; (Touvron et al., 2023)). LLaMA is a foundational LLM intended to be fine-tuned for downstream tasks by researchers or private and commercial users. In contrast to GPT, LLaMA was trained exclusively on publicly available data, such as Wikipedia texts, public GitHub repositories, and forum conversations, and can be freely downloaded and applied. Notably, LLaMA is significantly more efficient in training and operating than its contemporaries and can achieve state-of-the-art performance with significantly fewer parameters. For example, LLaMA with 13 billion parameters outperforms GPT3 with 175 billion parameters on most tasks (Touvron et al., 2023). Thus, LLaMA can be fine-tuned and run on consumer-grade hardware. Recent advances in quantization methods, used to reduce the number of parameters and accelerate inference speed for LLMs, further increase LLaMA's efficiency and applicability on home-grade computer hardware (Dettmers & Zettlemoyer, 2023). Consequently, a plethora of LLaMA derivatives, such as Stanford's "Alpaca" (Taori et al., 2023) and LMSYS' Vicuña (Chiang et al., 2023), have been fine-tuned on public data sets for various tasks, and in the spirit of open-science, have the full model (including model parameters/weights) as well training procedures and data publicly available (see e.g., https://huggingface.co/spaces/HuggingFaceH4/open_llm_leaderboard for an overview of best-performing LLMs on huggingface.com). Thus, LLaMA and its derivatives can be useful tools for researchers who want to be in control of their research pipeline while studying LLMs or applying them for further tasks.

S5a. Text Annotations

Data. Analogue to the main analyses, we tested LLaMA's performance on the Moral Foundations Reddit Corpus (MFRC; Trager et al. (2022b)), a collection of 16,123 Reddit comments that have been hand-annotated by at least three trained annotators for 8 categories of moral sentiment (i.e., Care, Proportionality, Equality, Purity, Authority, Loyalty, Thin Morality, Non-moral) based on the updated Moral Foundations Theory (MFT; Atari, Haidt, et al. (2023a)) framework. The MFRC further provides demographic and psychometric information about each human annotator. We used the same random subsection of the MFRC (2,983 samples) to evaluate the annotation accuracy of LLaMA.

LLaMA Model. We utilized the "Luna-AI-Llama2-Uncensored" model from huggingface.com, which is a Llama2 based chat model that was fine-tuned on over 40,000 long form chat discussions. We intentionally used the smallest and most efficient version ("Luna-AI-Llama2-Uncensored-GPTQ:gptq-4bit-128g-actorder_True"), which has 7 billion parameters and using 4-bit quantization, to show that our pipeline and performance is achievable for the average lab. This version is able to run locally on desktops with a single, mid-range, GPU of 6GB VRAM or even on laptops using the CPU. Note that "Uncensored", here refers to the model not being fine-tuned for appropriate content generation or any other policies. The model is intended to be fine-tuned according to the respective use-cases by the end-user. We chose this model because we wanted to avoid unnatural responses due to fine-tuning that deviates from regular human behavior (e.g., unnaturally helpful, moral, appropriate responses, "As an AI model, ...").

We prompted ChatGPT to annotate posts from the MFRC test data via "text-generation-web-ui" (Anonymous, 2023). This simple interface allows to self-host LLMs on a local machine. See the GitHub repository for full installation instructions, including a one-click installer. After installation, we downloaded the respective LLaMA model from Huggingface using the interface ("TheBloke/Luna-AI-Llama2-Uncensored-GPTQ:gptq-4bit-128g-actorder_True"). We then ran all inferences through Python via the interface's API function. See our code repository for instructions on how to run the LLaMA sentiment analysis via Python interactive notebook. We deployed the LLaMA model with a minimal temperature of 0.01, for maximal

reproducibility. We used the following prompt, which matched the definitions of each moral foundation according to MFT and the human annotators' instructions, to collect the annotations:

"USER: These are definitions of moral sentiments: "care" if a text is about avoiding emotional and physical damage to another individual, "equality" if a text is about equal treatment and equal outcome for individuals, "proportionality" if a text is about individuals getting rewarded in proportion to their merit or contribution, "loyalty" if a text is about cooperating with ingroups and competing with outgroups, "authority" if a text is about deference toward legitimate authorities and the defense of traditions, all of which are seen as providing stability and fending off chaos, "purity" if a text is about avoiding bodily and spiritual contamination and degradation, "thin-morality" if a text has a moral sentiment but cannot be categorized as either of the above, "none" if no moral sentiment is expressed in the text.
Based on this definition, name all moral sentiments that are expressed in the following text:
[TEXT TO ANNOTATE]
Return a comma-separated list of all moral sentiments that were expressed in the text.
ASSISTANT: "

Note that we slightly changed the prompt from ChatGPT, because our LLaMA version uses a different instruction/chat template compared to ChatGPT (e.g., requires "ASSISTANT:" to mark the beginning of response generation). Importantly, we did not change any of the moral sentiment definitions and did not provide any additional information. The changes are surface-level changes in the presentation of the information (e.g., USER/ASSISTANT template).

Local Hardware. We used a consumer-grade computer using an NVIDIA RTX2070s 8GB VRAM GPU, an AMD Ryzen 5 3600 3.6GHz 6-core CPU, 16GB DDR4 3200MHz RAM, and a 1TB HDD. The full set-up, including all additional components, cost less than $1,000 at the time of purchase (February 2021).

BERT-model. To keep the bias analyses comparable, no new BERT-based model was trained for this analysis. Instead, we utilized the previously trained and fine-tuned BERT model (see section X for the details of model training). We used the same model output as in the main

analysis to compare LLaMA's annotation performance against fine-tuned BERT's and for all subsequent analyses, such as the distribution of predicted moral sentiment or deviations from ground truth.

Performance Evaluation. To evaluate the annotation accuracy of the BERT-based model and LLaMA, we calculated each model's F1 score (Goutte & Gaussier, 2005), which is a widely used single metric that assesses the model's ability to make accurate positive predictions while minimizing false positives and false negatives. We calculated the F1 scores with the human annotations as ground truth using the Sklearn library (v1.3; Pedregosa et al. (2011)) in Python. We calculated the macro averaged F1 score, that is the unweighted average of the F1 scores for the prediction of each type of moral sentiment, which expresses the model's accuracy across all moral sentiments. Finally, we fit a logistic regression model predicting the presence of moral sentiment in an annotation (binary, yes/no) as a function of annotator type (Human, LLaMA, BERT). This model expressed how much more likely the computational models were to annotate a moral sentiment compared to human ground truth. This model was used to evaluate whether LLaMA or BERT significantly over-predicted or under-predicted (e.g., false positives, false negatives) the specific moral sentiments.

Bias Analyses. To determine biases in LLaMA's annotations, we investigated how much LLaMA aligned with annotators of specific demographics. To that end, we create a binary variable expressing whether LLaMA's annotation and the human annotators' rating aligned (1 if LLaMA and human annotation were equal; 0 if not). If there were multiple sentiments in one post we compared each one separately. We then fit logistic regression models that predicted human-LLaMA alignment as a function of annotator demographics and psychometric information. These models describe how likely LLaMA is to align with an annotator of certain demographics (e.g., race, gender, age). In total, we tested for bias towards annotators' age, sex, religion, moral values (based on MFT), personality (Five-Factor Model), creativity (creative imagination, aesthetic sensitivity, intellectual curiosity), mental health (anxiety, depression, emotional volatility), political orientation (collectivism, individualism, cultural-tightness, social-conservatism, economical-conservatism), interpersonal attitudes (compassion, trust, respectfulness), and efficiency (productiveness, organization, responsibility).

We conducted all statistical analyses in R (v4.1.2; R Core Team (2021)) using the stats (v4.1.2; R Core Team (2021)) package (R Core Team, 2021). See the code repository for instructions to replicate the statistical analyses.

Results. As expected, we found that fine-tuned BERT outperforms LLaMA by a large margin. Specifically, BERT achieved an F1 score of 0.48 while LLaMA achieved an F1 score of 0.23. Notably, however, LLaMA achieved a higher F1 score than ChatGPT (0.22) despite its significantly smaller model size and having been provided less computing power, highlighting the applicability of open LLMs.

When comparing the prediction errors across moral sentiments, we found that LLaMA and ChatGPT perform comparably. For example, LLaMA was significantly better at predicting Equality and Nonmoral sentiment, performed approximately on par when predicting Purity, Thin-Morality, Authority, and Proportionality sentiment, and performed significantly worse when predicting Care and Loyalty sentiment. See Table S34 for an overview of BERT, ChatGPT, and LLaMA's deviation from human ground truth.

Demographic Biases. We found that LLaMA2 showed significant demographic biases in its moral annotations, aligning more so with certain groups including older over younger(+3%, $p < .001$), male over female (+60 %, $p < .001$), and non-religious over Christian (+68%, $p < .001$). See Table S35 for an overview of all annotation biases.

Moral Biases. We found that LLaMA2 showed significant moral biases in its annotations, aligning more so with human annotators who endorse certain moral values, such as loyalty (+1052%, $p < .001$) and equality(+1358%, $p < .001$), while misaligning with the annotators who endorse other values, such as care (-99%, $p < .001$) and proportionality (-99%, $p < .001$). See Table S36 for an overview of annotation biases regarding annotators' moral values.

Personality Biases. We found that LLaMA2 showed significant personality biases in its annotations, aligning more so with human annotators who load highly on some personality traits, such as conscientiousness (+316%, $p < .001$) and extraversion (+88%, $p < .001$), while misaligning with annotators who load highly on other personality traits, such as neuroticism (-42%, $p < .001$). See Table S37 for an overview of annotation biases regarding annotators' personalities.

Table S34

Deviations from ground truth for each model on each prediction class

Class	BERT	ChatGPT	LLaMA
Care	+12% (p = .162)	+35% (p < .001)	+171% (p < .001)
Equality	-11% (p = .255)	+55% (p < .001)	-7% (p < .001)
Proportionality	-20% (p = .118)	-54% (p < .001)	+270% (p < .001)
Loyalty	-64% (p < .001)	+3% (p < .001)	+811% (p < .001)
Authority	-32% (p = .003)	+96% (p < .001)	+154% (p < .001)
Purity	-74% (p < .001)	-86% (p < .001)	+1,042% (p < .001)
Thin-Morality	+2% (p = .861)	+1,240% (p < .001)	-100% (p < .001)
Nonmoral	+69% (p < .001)	-100% (p < .001)	+12% (p < .001)

Note. *Table shows the difference in odds for the classifier to predict a class compared to ground truth (trained human annotators). The table shows that BERT has a smaller deviation from ground truth except on the Loyalty foundation. For most moral values BERT does* ***not*** *deviate significantly (at p < .05) from ground-truth while both LLMs (GPT, LLaMA2) deviate significantly from ground-truth for all moral values.*

Table S35

Demographic Biases of LLaMA2 annotations on the MFRC

Demographic Variable	$\Delta odds$	p
Female (vs. Male)	-60 %	< .001
Age (years)	+3%	< .001
Non-Religious (vs. Christianity)	+68%	< .001

Note. *Table shows LLaMA2 bias towards respective demographics during annotations. That is, it had higher (+) or lower (-) odds (%) of aligning with human annotators of said demographic.*

Table S36

Moral Biases of LLaMA2 annotations on the MFRC

Moral Foundation	$\Delta odds$	p
Care	-99%	$< .001$
Equality	+1358%	$< .001$
Proportionality	-99%	$< .001$
Loyalty	+1052%	$< .001$
Authority	-22%	.072
Purity	-	-

Note. *Table shows LLaMA2 bias towards respective moral profiles during annotations. That is, it had higher/lower odds (%) of aligning with human annotators endorsing said moral values. All variables are 5-point Likert scales.*

Table S37

Personality Biases of LLaMA2 annotations on the MFRC

Demographic Variable	$\Delta odds$	p
Openness	+0%	.993
Conscientiousness	+316%	$< .001$
Extraversion	+88%	$< .001$
Agreeableness	-10%	.507
Neuroticism	-42%	$< .001$

Note. *Table shows LLaMA2 bias towards respective demographics during annotations. That is, LLaMA2 had higher (+) or lower (-) odds (%) of aligning with human annotators of said personalities. All variables are 5-point Likert scales.*

Political Biases. We found that LLaMA2 showed significant political biases in its annotations, aligning more so with human annotators who have certain political attitudes, such as collectivism (+20%, $p < .001$), tightness (+388%, $p < .001$), social conservatism (+3%, $p < .001$), and economic conservatism (+4%, $p < .001$), while misaligning with the annotators who have other political attitudes, such as individualism (-77%, $p < .001$). See Table S38 for an overview of annotation biases regarding annotators' political orientation.

Table S38

Political Biases of LLaMA2 annotations on the MFRC

Socio-Political Variable	$\Delta odds$	p
Collectivism	+20%	$< .001$
Individualism	-77%	$< .001$
Tightness	+388%	$< .001$
Social conservatism	+3%	$< .001$
Economical conservatism	+4%	$< .001$

Note. *Table shows LLaMA2 bias towards respective demographics during annotations. That is, LLaMA2 had higher (+) or lower (-) odds (%) of aligning with human annotators of said political values. All variables are 5-point Likert scales.*

Creativity-related Biases. We found that LLaMA2 showed significant creativity-related biases in its annotations, aligning more so with human annotators who have certain creativity-related traits, such as openness (+880%, $p < .001$) and aesthetic sensitivity (+81%, $p < .001$) while misaligning with annotators who have other traits, such as creative imagination (-96%, $p < .001$). See Table S39 for an overview of annotation biases regarding annotators' creativity and aesthetic preferences.

Mental Health Biases. We found that LLaMA2 showed significant mental health biases in its annotations, aligning more so with human annotators who have certain mental health traits, such as depression (+47%, $p < .001$) while misaligning with the annotators who have other mental health traits, such as anxiety (-58%, $p < .001$) and emotional volatility (-35%, $p < .001$).

Table S39

Creativity-related Biases of LLaMA2 annotations on the MFRC

Variable	$\Delta odds$	p
Openness	+880%	$< .001$
Creative imagination	-96%	$< .001$
Aesthetic sensitivity	+81%	$< .001$

Note. *Table shows LLaMA2 bias towards respective demographics during annotations. That is, LLaMA2 had higher (+) or lower (-) odds (%) of aligning with human annotators of said creative inclinations. All variables are 5-point Likert scales.*

See Table S40 for an overview of annotation biases regarding annotators' mental health traits.

Table S40

Mental-Health related Biases of ChatGPT annotations on the MFRC

Mental-Health Variable	$\Delta odds$	p
Anxiety	-58%	$< .001$
Depression	+47%	$< .001$
Emotional-Volatility	-35%	$< .001$

Note. *Table shows LLaMA2 bias towards respective demographics during annotations. That is, LLaMA2 had higher (+) or lower (-) odds (%) of aligning with human annotators with said mental-health issues. All variables as 5-point Likert scales.*

Interpersonal virtues Biases. We found that LLaMA2 showed significant interpersonal virtues biases in its annotations, aligning more so with human annotators who have certain interpersonal virtues, such as trust (+126%, $p < .001$) and respectfulness (+140%, $p < .001$) while misaligning with the annotators who have other interpersonal virtues, such as compassion (-36%, $p < .001$). See Table S41 for an overview of annotation biases regarding annotators' interpersonal virtues.

Table S41

Interpersonal Virtues related Biases of LLaMA2 annotations on the MFRC

Virtue Variable	$\Delta odds$	p
Compassion	-36%	$< .001$
Trust	+126%	$< .001$
Respectfulness	+140%	$< .001$

Note. *Table shows LLaMA2 bias towards respective demographics during annotations. That is, LLaMA2 had higher (+) or lower (-) odds (%) of aligning with human annotators of said interpersonal virtues. All variables as 5-point Likert scales.*

Efficiency-related Biases. We found that LLaMA2 showed significant efficiency-related trait biases in it's annotations, aligning more so with human annotators who have high endorsement of all efficiency-related traits, such as productiveness (+25%, $p < .001$), organization (+269%, $p < .001$) and responsibility (+98%, $p < .001$). See Table S42 for an overview of annotation biases regarding annotators' efficiency traits.

Table S42

Efficiency-related Biases of LLaMA2 annotations on the MFRC

Efficiency Variable	$\Delta odds$	p
Productiveness	+25%	$< .001$
Organization	+269%	$< .001$
Responsibility	+98%	$< .001$

Note. *Table shows LLaMA2 bias towards respective demographics during annotations. That is, LLaMA2 had higher (+) or lower (-) odds (%) of aligning with human annotators of said efficiency traits. All variables as 5-point Likert scales.*

S5b. Survey Responses

Data. We repeated our tests on the same questionnaires as in the main analyses, namely, the Big-Five Inventory (BFI; $N = 3924$; Fossati et al. (2011)), Need for Closure Survey ($N = 315$; Webster and Kruglanski (1994b)), Need for Cognition Survey ($N = 900$; Cacioppo and Petty (1982b)), Right-wing-authoritarianism Scale ($N = 1020$; Zakrisson (2005b)), Emphasizing-Systemizing Scale ($N = 3141$; Baron-Cohen et al. (2003b)), and Rational-Experiential Inventory Scale ($N = 1456$; Pacini and Epstein (1999b)). We used the same human response data as in the main analyses, which was collected on https://www.yourmorals.org and contains the participants' responses as well as various demographic information about the participants.

LLaMA Responses. We prompted LLaMA to respond to survey items via the "text-generation-web-ui" interface (Anonymous, 2023) in Python. We deployed the "Luna-AI-Llama2-Uncensored-GPTQ:gptq-4bit-128g-actorder_True" model with a temperature of 0.76 to allow for variability in the responses. Note that ChatGPT's and LLaMA's parameters have different ranges and that LLaMA has significantly more parameters than ChatGPT. Since tuning these parameters for better data generation is beyond the scope of this work, we chose a popular preset of LLaMA parameters that achieves high performances in instruction following and chat response benchmark. However, LLaMA might be able to produce better performances with different sets of parameter values. See our code repository for the set of chosen parameters. We, again, gave LLaMA slightly changed prompts due to its different instruction template compared to ChatGPT. The prompts had this general form:

"USER: You will indicate your general level of agreement with a statement given to you. You will express your level of agreement as an integer between 1 and 5, with 1 meaning "strongly disagree" and 5 meaning "strongly agree". You will respond with nothing but this number. How much do you agree with this statement?
[STATEMENT]
ASSISTANT: "

We did not add any additional information to the LLaMA prompt and only changed the presentation and tone of the instruction details (this LLaMA version expected more direct instructions than ChatGPT, e.g., marking the beginning of response generation with "ASSISTANT"). We adapted the scale descriptions to the surveys as necessary. We collected 100 responses for each item (that is, we repeated the same prompt 100 times) to allow for analyses regarding the variance within LLaMA responses. See the project repository for the utilized survey items, LLaMA prompts, and code containing the API calls.

Bias Analyses. We compared LLaMA's responses to demographic-level average human responses. That is, we investigated the difference between LLaMA's survey responses and the average response of different groups of humans based on their demographics (e.g., age, race, gender). Note, we removed all demographics with less than 100 samples in the data sets as they did not provide enough information to make inferences about the said demographic. We calculated the average LLaMA response for each construct in a given survey and then subtracted it from the group-level average human response on said construct (e.g., the difference between LLaMA's "Openness" score and the average male or female "Openness" score). We then tested these differences for statistical significance using Dunnett's test (Dunnett, 1955), which compares each of a number of treatments with a single control, with the demographic groups as treatments and LLaMA as the control, while adjusting for multiple comparisons. We then repeated this analysis for differences in variance. We investigated whether LLaMA responses had less variance compared to the different human groups (e.g., the difference in the variance of LLaMA's "Openness" scores vs. the variance in male or female "Openness" scores). We used the Levene Test (Levene, 1960) to determine whether the variance in each human group's responses differed from LLaMA's while accounting for multiple comparisons using Bonferroni correction. Second, we investigated the relationship between the participants' demographic characteristics and the similarity of humans to LLaMA responses. We fit a linear regression model that predicted the absolute deviation of LLaMA and human responses (aggregated over all survey constructs) as a function of each human participant's demographic characteristic. These models expressed which participant demographics LLaMA is more or less likely to align with and therefore showed its biases (e.g., are LLaMA's responses more similar to older vs. younger participants).

We conducted all analyses in R (v4.1.2; R Core Team (2021)) using the stats (v4.1.2; R Core Team (2021)), car (v3.0-11; Fox and Weisberg (2019)), and DescTools (v0.99.44; Andri et mult. al. (2021)) packages.

Results. Overall, we found that LLaMA generates responses comparable to ChatGPT. Figures S32 to S42 show the differences between LLaMA, ChatGPT, and human demographic groups. Notably, on some questionnaires, LLaMA achieves significantly more natural responses that are better aligned with the different human demographics. This might be linked to the lack of "corporate guardrails" and censorship policies. For example, LLaMA is significantly less Agreeable and Conscientious than ChatGPT which might be linked to ChatGPT being fine-tuned to be a friendly assistant that avoids sensitive topics. Tables S43 - S51 provide a detailed overview of the differences between LLaMA and various human demographic groups.

Table S43

Deviation of LLaMA responses from human responses grouped by age

**Survey**	**Construct**	**Demographic**	***d***	***p***
BFI	Extraversion	55-74	-0.19	.076
BFI	Agreeableness	55-74	0.08	.495
BFI	Conscientiousness	55-74	0.21	.015
BFI	Neuroticism	55-74	-0.14	.263
BFI	Openness	55-74	0.38	< .001
Need for Cognition	Need for Cognition	55-74	0.63	< .001
Need for Closure	Need for Closure	55-74	-	-
Systemizing and Emphasizing	Systemizing	55-74	-0.24	< .001
Systemizing and Emphasizing	Emphasizing	55-74	0.29	< .001
Rational-Experiential Inventory	Rational	55-74	0.77	< .001
Rational-Experiential Inventory	Experiential	55-74	-0.08	.614
Right-Wing-Authoritarianism	Right-Wing-Authoritarianism	55-74	-0.85	< .001
BFI	Extraversion	35-54	-0.26	.008
BFI	Agreeableness	35-54	-0.01	1.000

Continued on next page

Table S43 – continued from previous page

**Survey**	**Construct**	**Demographic**	***d***	***p***
BFI	Conscientiousness	35-54	0.06	.669
BFI	Neuroticism	35-54	-0.02	.989
BFI	Openness	35-54	0.25	$< .001$
Need for Cognition	Need for Cognition	35-54	0.70	$< .001$
Need for Closure	Need for Closure	35-54	-	-
Systemizing and Emphasizing	Systemizing	35-54	-0.22	$< .001$
Systemizing and Emphasizing	Emphasizing	35-54	0.23	$< .001$
Rational-Experiential Inventory	Rational	35-54	0.83	$< .001$
Rational-Experiential Inventory	Experiential	35-54	0.02	.998
Right-Wing-Authoritarianism	Right-Wing-Authoritarianism	35-54	-0.69	$< .001$
BFI	Extraversion	25-34	-0.35	$< .001$
BFI	Agreeableness	25-34	-0.09	.339
BFI	Conscientiousness	25-34	-0.088	.437
BFI	Neuroticism	25-34	0.100	.457
BFI	Openness	25-34	0.19	.003
Need for Cognition	Need for Cognition	25-34	0.66	$< .001$
Need for Closure	Need for Closure	25-34	-	-
Systemizing and Emphasizing	Systemizing	25-34	-0.32	$< .001$
Systemizing and Emphasizing	Emphasizing	25-34	0.18	$< .001$
Rational-Experiential Inventory	Rational	25-34	0.77	$< .001$
Rational-Experiential Inventory	Experiential	25-34	-0.04	.960
Right-Wing-Authoritarianism	Right-Wing-Authoritarianism	25-34	-0.60	$< .001$
BFI	Extraversion	18-24	-0.30	.001
BFI	Agreeableness	18-24	-0.08	.475
BFI	Conscientiousness	18-24	-0.19	.018
BFI	Neuroticism	18-24	0.289	.001

Continued on next page

Table S43 – continued from previous page

**Survey**	**Construct**	**Demographic**	***d***	***p***
BFI	Openness	18-24	0.06	.608
Need for Cognition	Need for Cognition	18-24	0.48	< .001
Need for Closure	Need for Closure	18-24	-	-
Systemizing and Emphasizing	Systemizing	18-24	-0.43	< .001
Systemizing and Emphasizing	Emphasizing	18-24	0.11	.052
Rational-Experiential Inventory	Rational	18-24	0.56	< .001
Rational-Experiential Inventory	Experiential	18-24	0.04	.956
Right-Wing-Authoritarianism	Right-Wing-Authoritarianism	18-24	-0.51	< .001

Note: *Positive values indicate a higher average response for age groups compared to LLaMA. Significance determined via Dunnett's Test.*

Figure S32

Comparing LLaMA and ChatGPT against Humans by Age for responses on the BFI

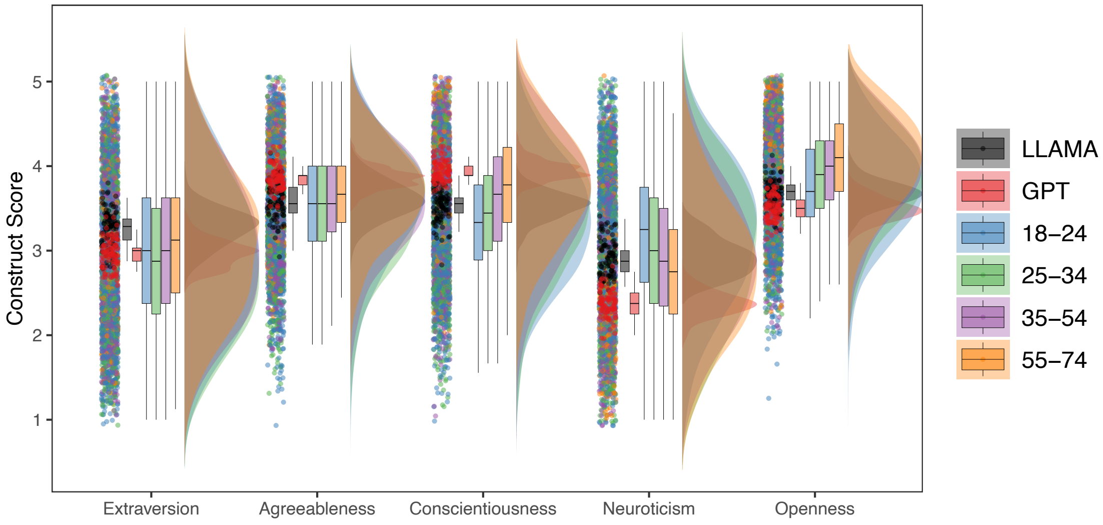

Figure S33

Comparing LLaMA and ChatGPT against Humans by Age for responses on the Need for Cognition questionnaire

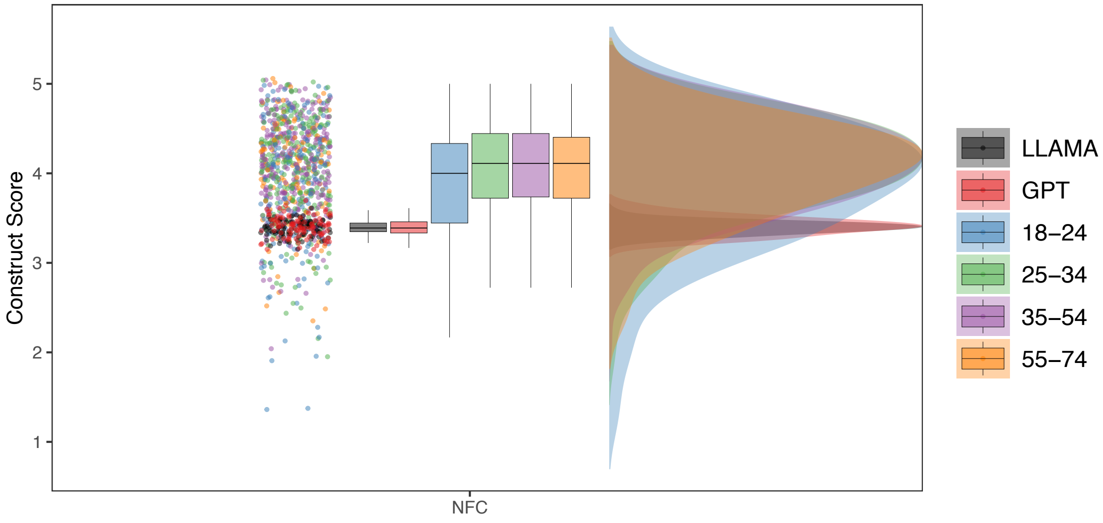

Figure S34

Comparing LLaMA and ChatGPT against Humans by Age for responses on the Rational-Experiential Inventory

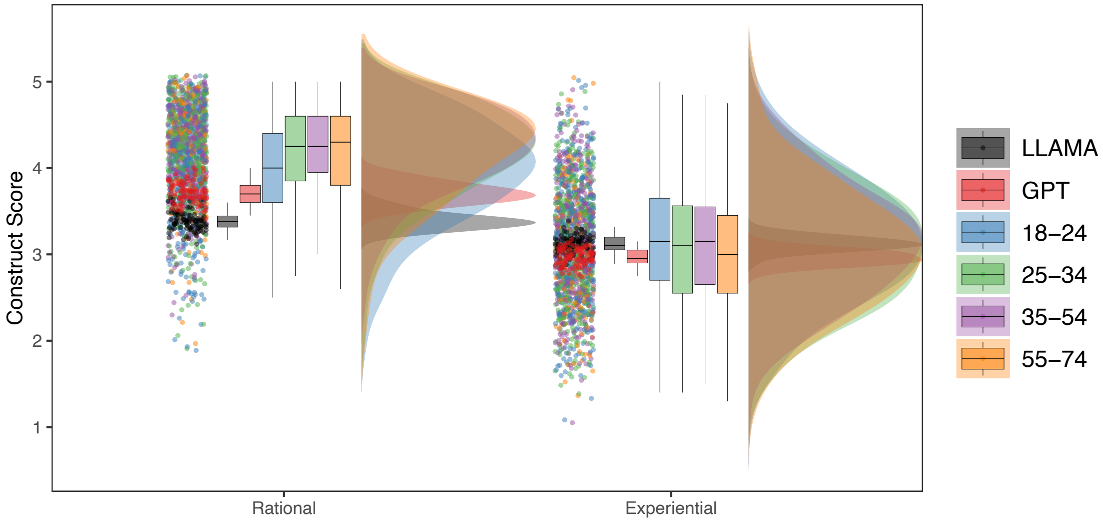

Figure S35

Comparing LLaMA and ChatGPT against Humans by Age for responses on the Right-Wing-Authoritarianism Scale

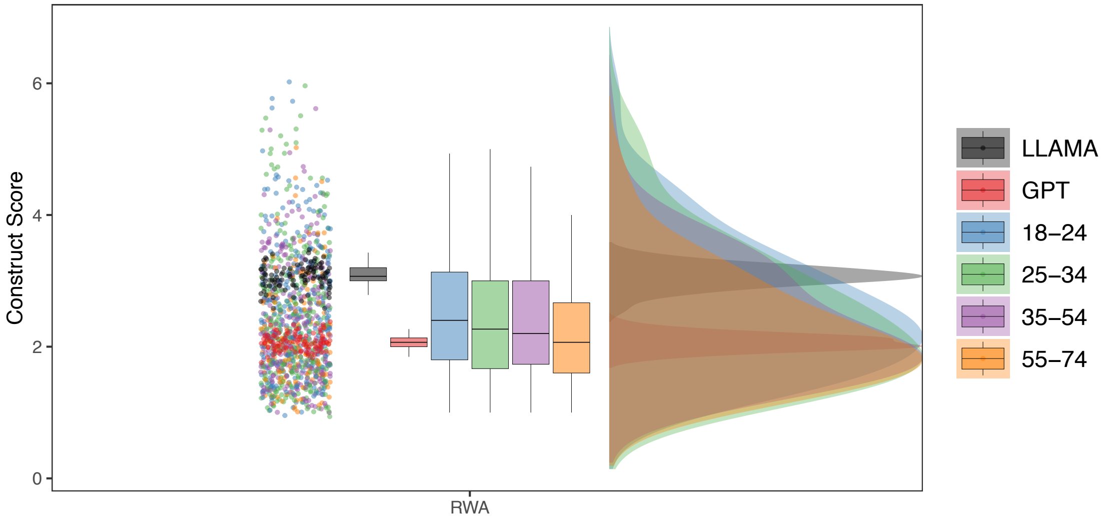

Figure S36

Comparing LLaMA and ChatGPT against Humans by Age for responses on the Systemizing-Emphasizing Scale

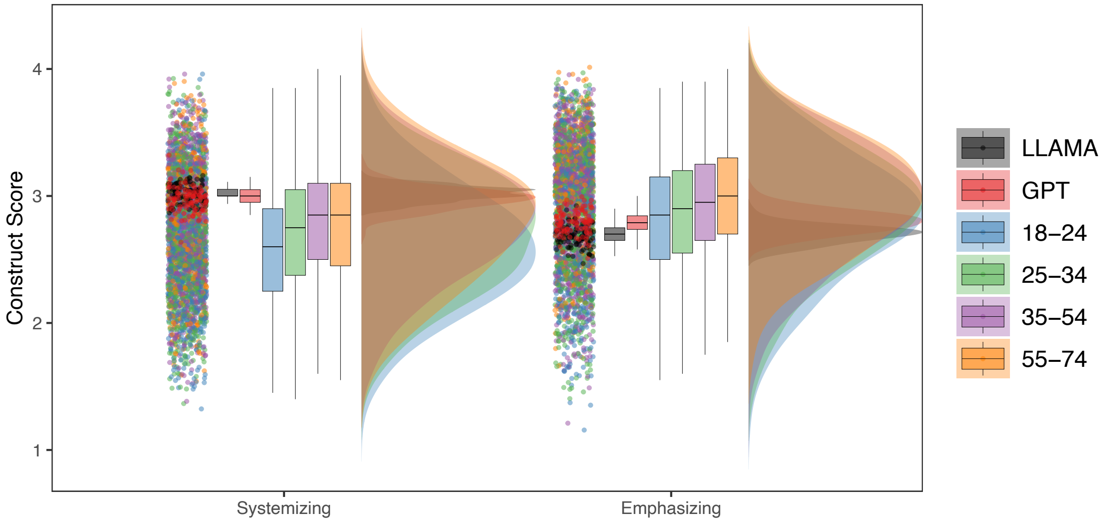

Table S45

Deviation of LLaMA responses from human responses grouped by sex

**Survey**	**Construct**	**Demographic**	***d***	***p***
BFI	Extraversion	Female	-0.20	.032
BFI	Agreeableness	Female	0.05	.637
BFI	Conscientiousness	Female	0.02	.949
BFI	Neuroticism	Female	0.35	$< .001$
BFI	Openness	Female	0.13	.057
Need for Cognition	Need for Cognition	Female	0.54	$< .001$
Need for Closure	Need for Closure	Female	-1.27	$< .001$
Systemizing and Emphasizing	Systemizing	Female	-0.53	$< .001$
Systemizing and Emphasizing	Emphasizing	Female	0.35	$< .001$
Rational-Experiential Inventory	Rational	Female	0.60	$< .001$
Rational-Experiential Inventory	Experiential	Female	0.14	.08
Right-Wing-Authoritarianism	Right-Wing-Authoritarianism	Female	-0.84	$< .001$
BFI	Extraversion	Male	-0.35	$< .001$
BFI	Agreeableness	Male	-0.126	.089
BFI	Conscientiousness	Male	-0.11	.241
BFI	Neuroticism	Male	-0.10	.352
BFI	Openness	Male	0.233	$< .001$
Need for Cognition	Need for Cognition	Male	0.71	$< .001$
Need for Closure	Need for Closure	Male	-1.35	$< .001$
Systemizing and Emphasizing	Systemizing	Male	-0.14	.002
Systemizing and Emphasizing	Emphasizing	Male	0.10	.064
Rational-Experiential Inventory	Rational	Male	0.82	$< .001$
Rational-Experiential Inventory	Experiential	Male	-0.11	.202
Right-Wing-Authoritarianism	Right-Wing-Authoritarianism	Male	-0.56	$< .001$

Continued on next page

Table S45 – continued from previous page

Survey	Construct	Demographic	*d*	*p*

Note: *Positive values indicate a higher average response for sex group compared to LLaMA. Significance determined via Dunnett's Test.*

Figure S37

Comparing LLaMA and ChatGPT against Humans by Sex for responses on the BFI

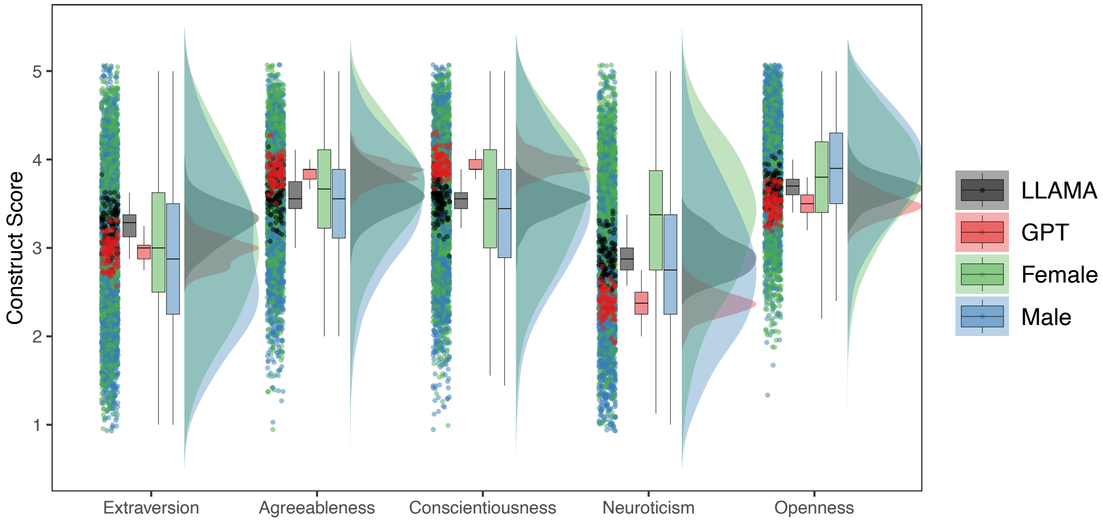

Figure S38

Comparing LLaMA and ChatGPT against Humans by Sex for responses on the Need for Cognition questionnaire

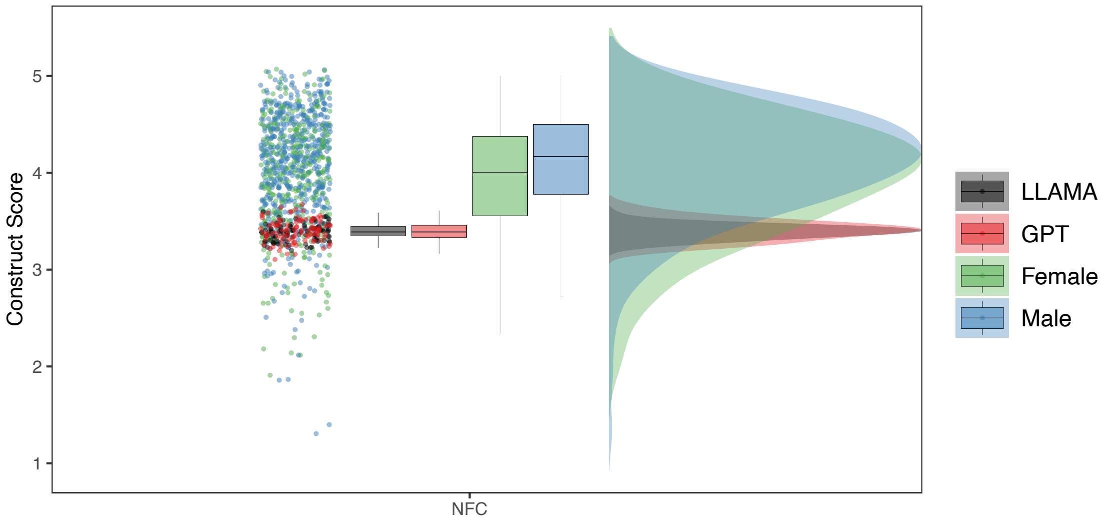

Figure S39

Comparing LLaMA and ChatGPT against Humans by Sex for responses on the Need for Closure Survey

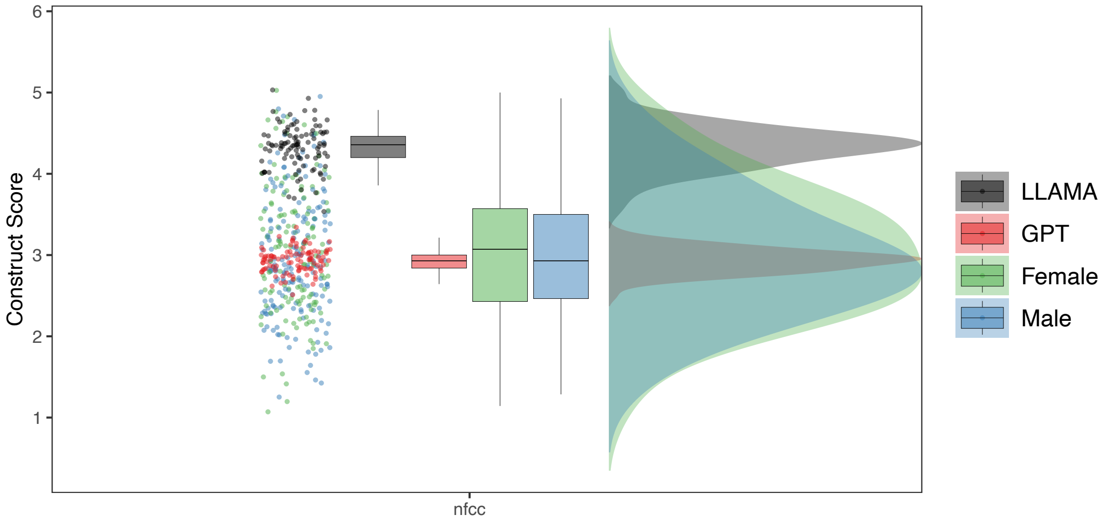

Figure S40

Comparing LLaMA and ChatGPT against Humans by Sex for responses on the Rational-Experiential Inventory

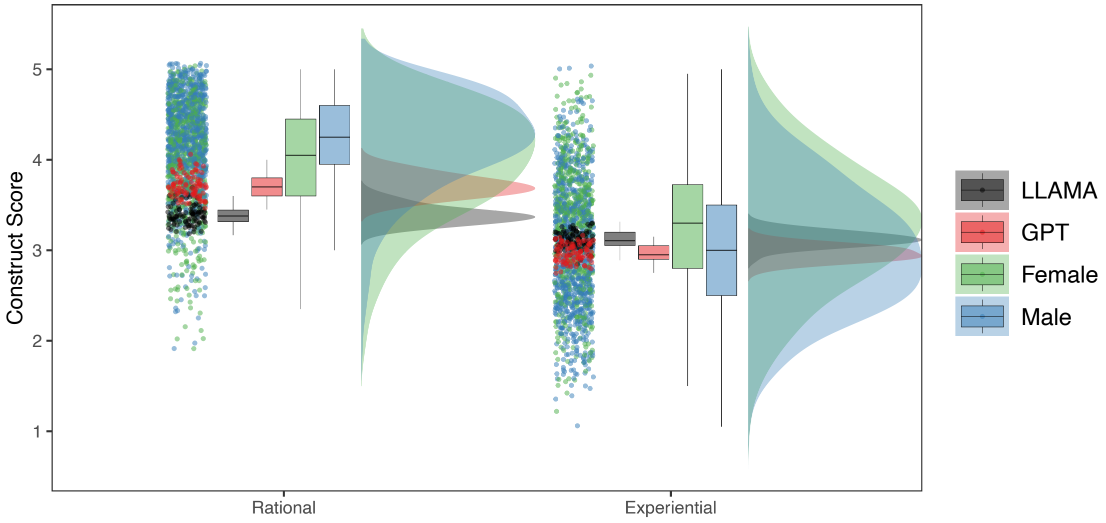

Figure S41

Comparing LLaMA and ChatGPT against Humans by Sex for responses on the Right-Wing-Authoritarianism Scale

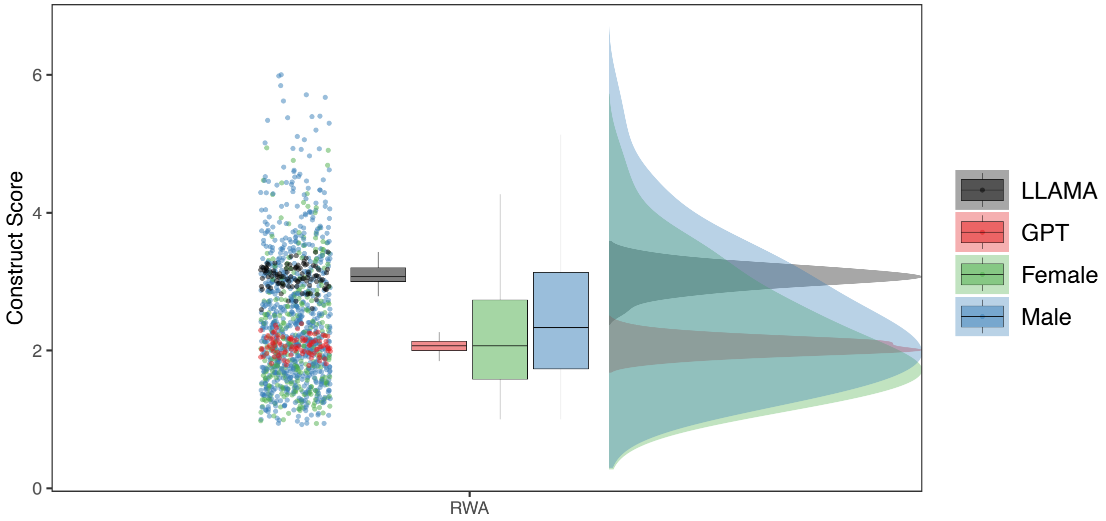

Figure S42

Comparing LLaMA and ChatGPT against Humans by Sex for responses on the Systemizing-Emphasizing Scale

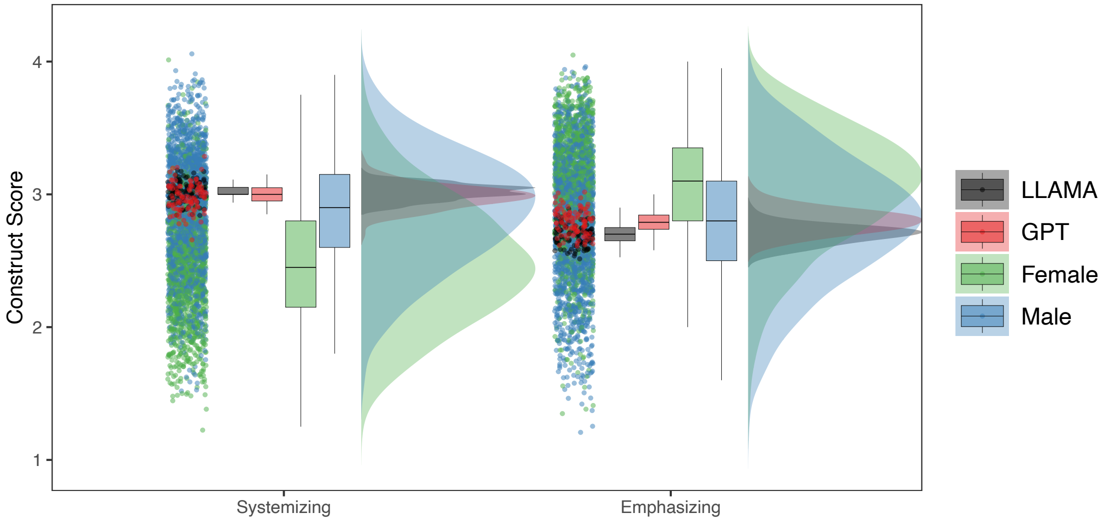

Table S47

Deviation of LLaMA responses from human responses grouped by race

**Survey**	**Construct**	**Demographic**	***d***	***p***
BFI	Extraversion	Asian	-0.39	< .001
BFI	Agreeableness	Asian	-0.16	.06
BFI	Conscientiousness	Asian	-0.20	.042
BFI	Neuroticism	Asian	0.11	.538
BFI	Openness	Asian	0.08	.512
Need for Cognition	Need for Cognition	Asian	-	-
Need for Closure	Need for Closure	Asian	-	-
Systemizing and Emphasizing	Systemizing	Asian	-0.30	< .001
Systemizing and Emphasizing	Emphasizing	Asian	0.14	.029
Rational-Experiential Inventory	Rational	Asian	0.58	< .001
Rational-Experiential Inventory	Experiential	Asian	-0.07	.706
Right-Wing-Authoritarianism	Right-Wing-Authoritarianism	Asian	-	-
BFI	Extraversion	White	-0.27	.006
BFI	Agreeableness	White	-0.29	.974
BFI	Conscientiousness	White	-0.020	.996
BFI	Neuroticism	White	0.09	.566
BFI	Openness	White	0.21	< .001
Need for Cognition	Need for Cognition	White	0.68	< .001
Need for Closure	Need for Closure	White	-1.38	< .001
Systemizing and Emphasizing	Systemizing	White	-0.29	< .001
Systemizing and Emphasizing	Emphasizing	White	0.20	< .001
Rational-Experiential Inventory	Rational	White	0.78	< .001
Rational-Experiential Inventory	Experiential	White	0.01	.998
Right-Wing-Authoritarianism	Right-Wing-Authoritarianism	White	-0.65	< .001
BFI	Extraversion	Hispanic	-0.29	.009

Continued on next page

Table S47 – continued from previous page

**Survey**	**Construct**	**Demographic**	***d***	***p***
BFI	Agreeableness	Hispanic	-0.11	.326
BFI	Conscientiousness	Hispanic	-0.08	.66
BFI	Neuroticism	Hispanic	0.21	.096
BFI	Openness	Hispanic	0.09	.430
Need for Cognition	Need for Cognition	Hispanic	-	-
Need for Closure	Need for Closure	Hispanic	-	-
Systemizing and Emphasizing	Systemizing	Hispanic	-0.38	< .001
Systemizing and Emphasizing	Emphasizing	Hispanic	0.18	.008
Rational-Experiential Inventory	Rational	Hispanic	-	-
Rational-Experiential Inventory	Experiential	Hispanic	-	-
Right-Wing-Authoritarianism	Right-Wing-Authoritarianism	Hispanic	-	-
BFI	Extraversion	Black	-0.33	.008
BFI	Agreeableness	Black	0.05	.935
BFI	Conscientiousness	Black	-0.03	.994
BFI	Neuroticism	Black	0.02	.999
BFI	Openness	Black	0.06	.832
Need for Cognition	Need for Cognition	Black	-	-
Need for Closure	Need for Closure	Black	-	-
Systemizing and Emphasizing	Systemizing	Black	-	-
Systemizing and Emphasizing	Emphasizing	Black	-	-
Rational-Experiential Inventory	Rational	Black	-	-
Rational-Experiential Inventory	Experiential	Black	-	-
Right-Wing-Authoritarianism	Right-Wing-Authoritarianism	Black	-	-

Note: *Positive values indicate a higher average response for racial groups compared to LLaMA. Significance determined via Dunnett's Test.*

Figure S43

Comparing LLaMA and ChatGPT against Humans by Race for responses on the BFI

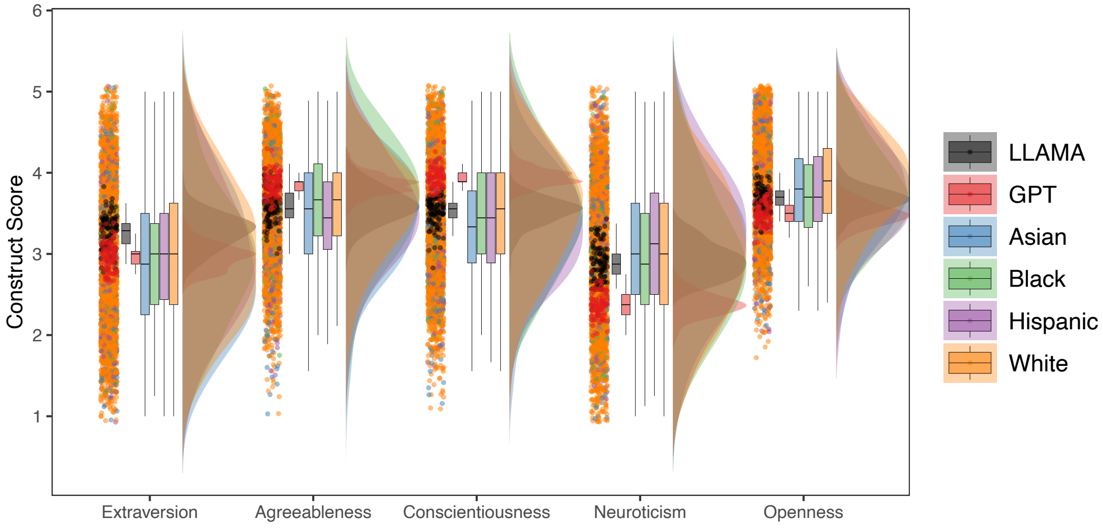

Figure S44

Comparing LLaMA and ChatGPT against Humans by Race for responses on the Need for Cognition questionnaire

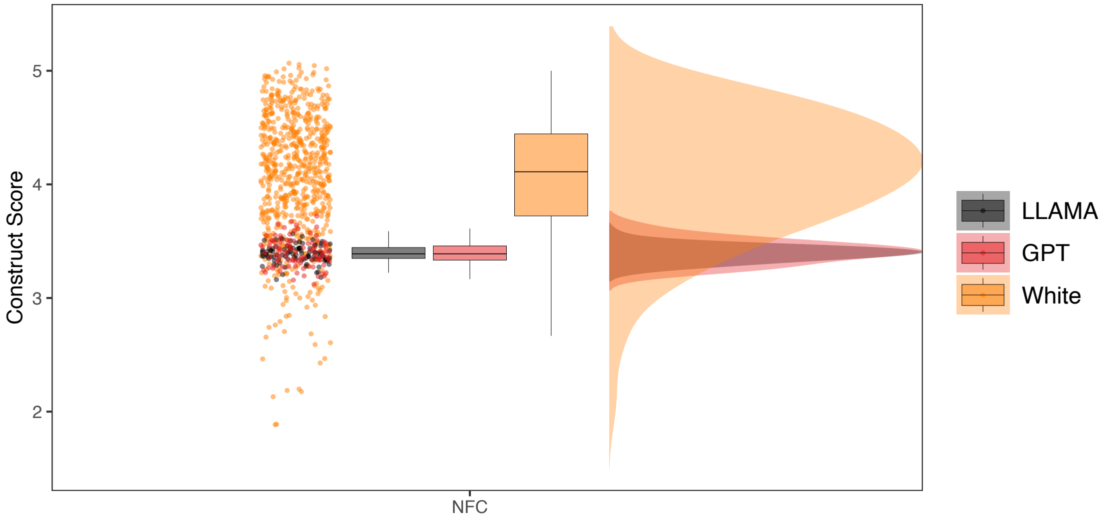

Figure S45

Comparing LLaMA and ChatGPT against Humans by Race for responses on the Need for Closure Survey

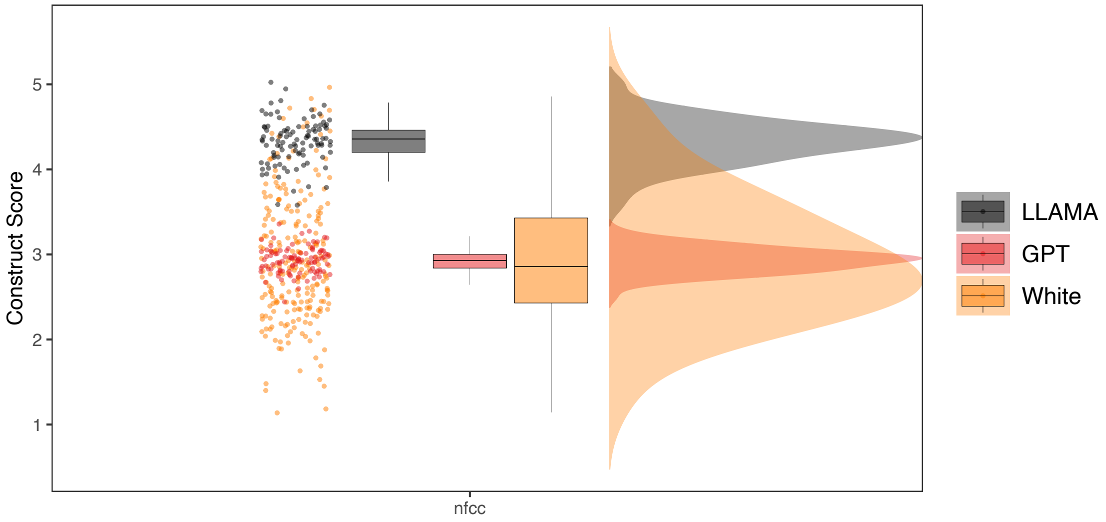

Figure S46

Comparing LLaMA and ChatGPT against Humans by Race for responses on the Rational-Experiential Inventory

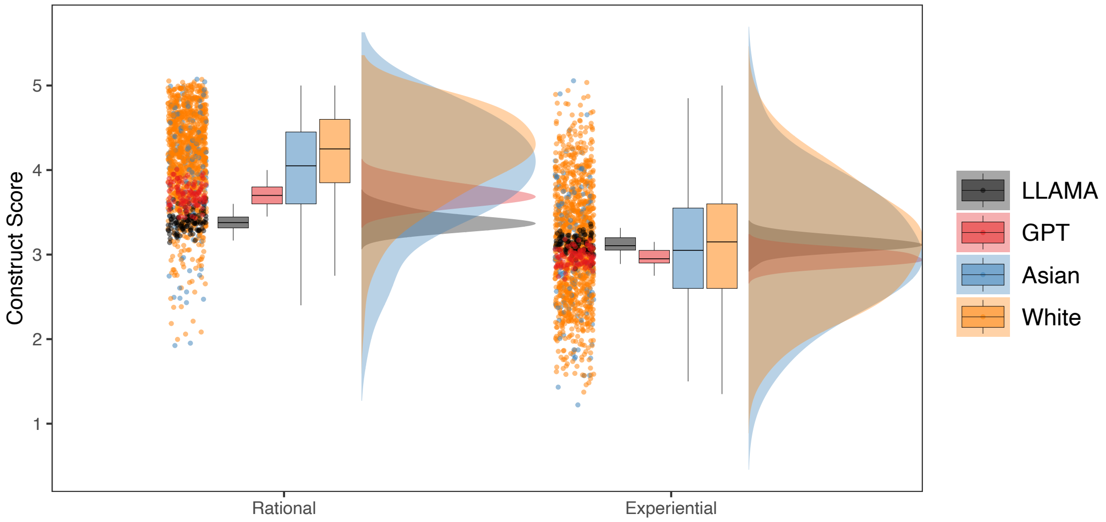

Figure S47

Comparing LLaMA and ChatGPT against Humans by Race for responses on the Right-Wing-Authoritarianism Scale

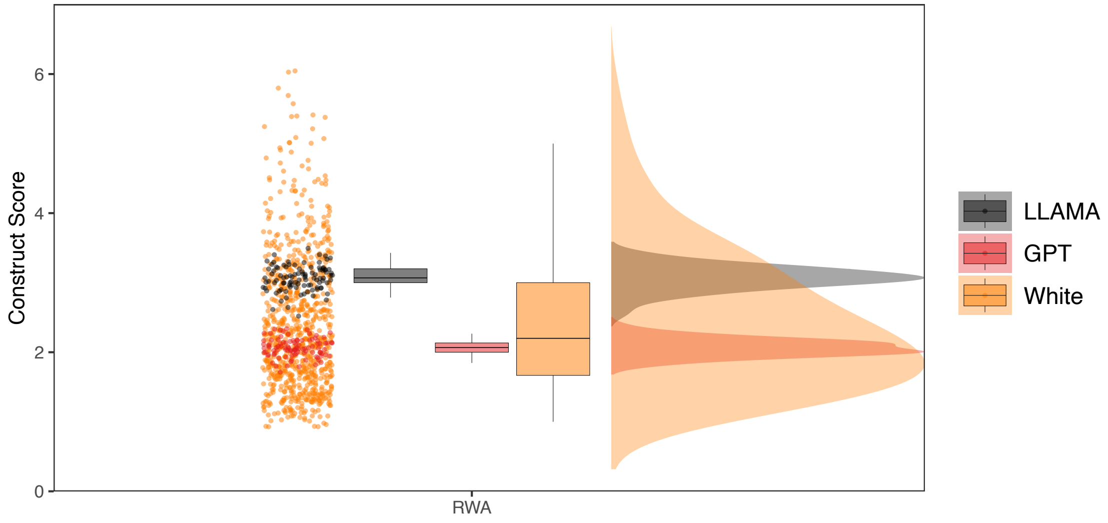

Figure S48

Comparing LLaMA and ChatGPT against Humans by Race for responses on the Systemizing-Emphasizing Scale

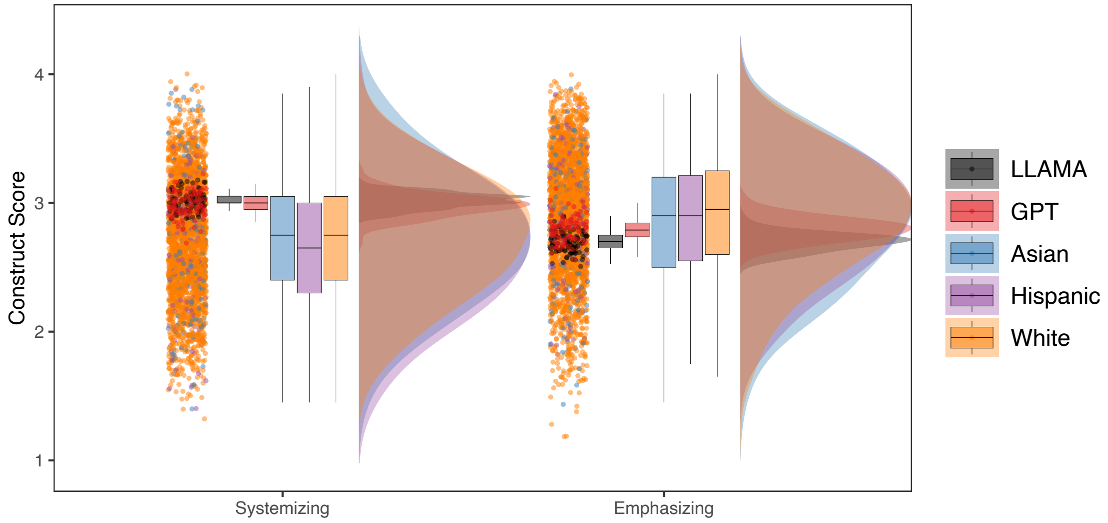

Table S49

Deviation of LLaMA responses from human responses grouped by religion

Survey	Construct	Demographic	*d*	*p*
BFI	Extraversion	Christianity	-0.20	.038
BFI	Agreeableness	Christianity	0.06	.608
BFI	Conscientiousness	Christianity	0.05	.804
BFI	Neuroticism	Christianity	0.05	.807
BFI	Openness	Christianity	0.09	.051
Need for Cognition	Need for Cognition	Christianity	0.54	< .001
Need for Closure	Need for Closure	Christianity	-	-
Systemizing and Emphasizing	Systemizing	Christianity	-0.33	< .001
Systemizing and Emphasizing	Emphasizing	Christianity	0.21	< .001
Rational-Experiential Inventory	Rational	Christianity	0.67	< .001
Rational-Experiential Inventory	Experiential	Christianity	0,04	.908
Right-Wing-Authoritarianism	Right-Wing-Authoritarianism	Christianity	0.01	.999
BFI	Extraversion	Atheist	-0.42	< .001
BFI	Agreeableness	Atheist	-0.16	.039
BFI	Conscientiousness	Atheist	-0.10	.295
BFI	Neuroticism	Atheist	0.11	.402
BFI	Openness	Atheist	0.24	< .001
Need for Cognition	Need for Cognition	Atheist	0.69	< .001
Need for Closure	Need for Closure	Atheist	-	-
Systemizing and Emphasizing	Systemizing	Atheist	-0.25	< .001
Systemizing and Emphasizing	Emphasizing	Atheist	0.14	.008
Rational-Experiential Inventory	Rational	Atheist	0.80	< .001
Rational-Experiential Inventory	Experiential	Atheist	-0.20	.013
Right-Wing-Authoritarianism	Right-Wing-Authoritarianism	Atheist	-1.10	< .001

Continued on next page

Table S49 – continued from previous page

**Survey**	**Construct**	**Demographic**	***d***	***p***
BFI	Extraversion	Agnostic	-0.32	$< .001$
BFI	Agreeableness	Agnostic	-0.10	.256
BFI	Conscientiousness	Agnostic	-0.13	.140
BFI	Neuroticism	Agnostic	0.18	.073
BFI	Openness	Agnostic	0.23	$< .001$
Need for Cognition	Need for Cognition	Agnostic	0.69	$< .001$
Need for Closure	Need for Closure	Agnostic	-	-
Systemizing and Emphasizing	Systemizing	Agnostic	-0.31	$< .001$
Systemizing and Emphasizing	Emphasizing	Agnostic	.22	$< .001$
Rational-Experiential Inventory	Rational	Agnostic	0.74	$< .001$
Rational-Experiential Inventory	Experiential	Agnostic	0.06	.736
Right-Wing-Authoritarianism	Right-Wing-Authoritarianism	Agnostic	-0.87	$< .001$

Note: *Positive values indicate a higher average response for religious groups compared to LLaMA. Significance determined via Dunnett's Test.*

Figure S49

Comparing LLaMA and ChatGPT against Humans by Religion for responses on the BFI

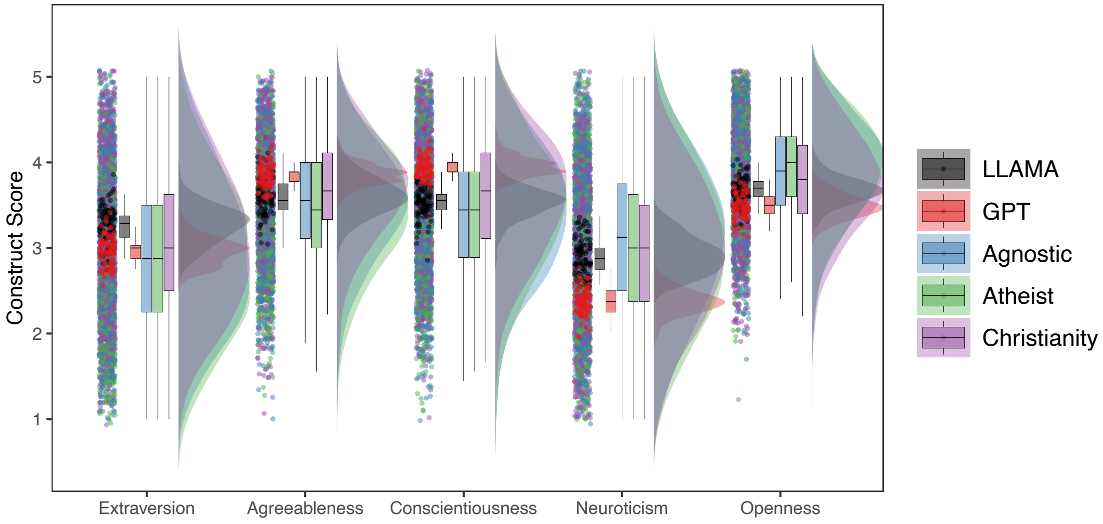

Figure S50

Comparing LLaMA and ChatGPT against Humans by Religion for responses on the Need for Cognition questionnaire

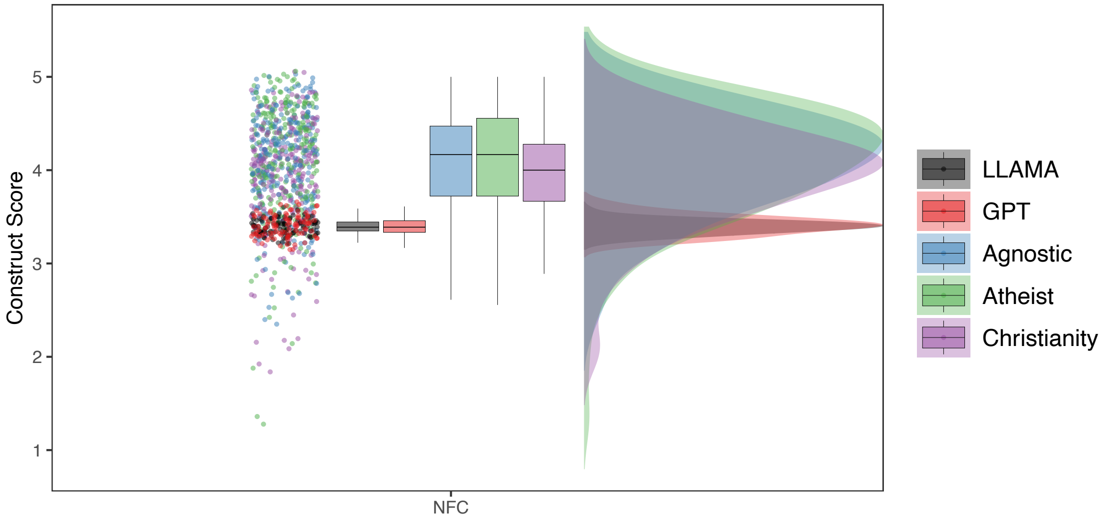

Figure S51

Comparing LLaMA and ChatGPT against Humans by Religion for responses on the Rational-Experiential Inventory

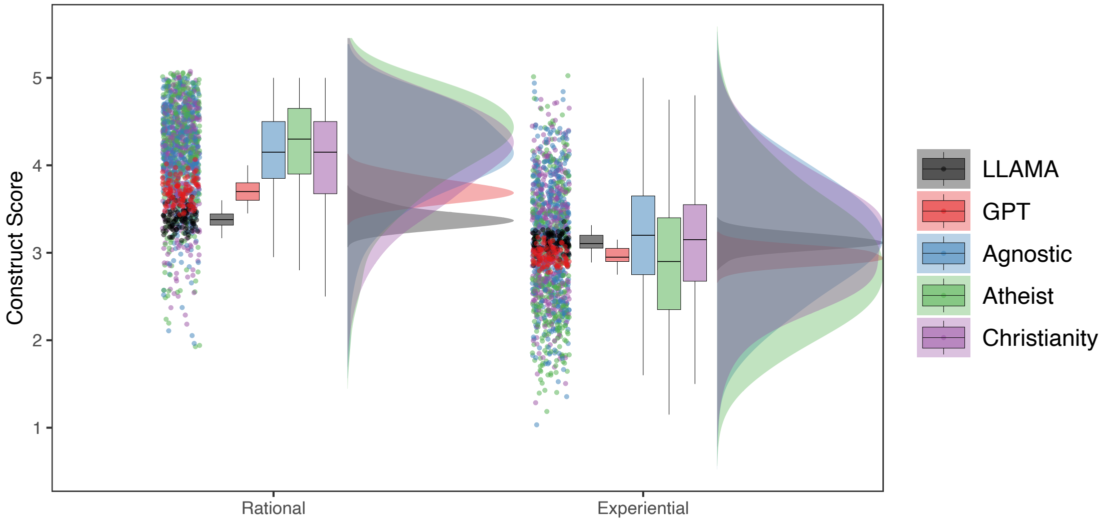

Figure S52

Comparing LLaMA and ChatGPT against Humans by Religion for responses on the Right-Wing-Authoritarianism Scale

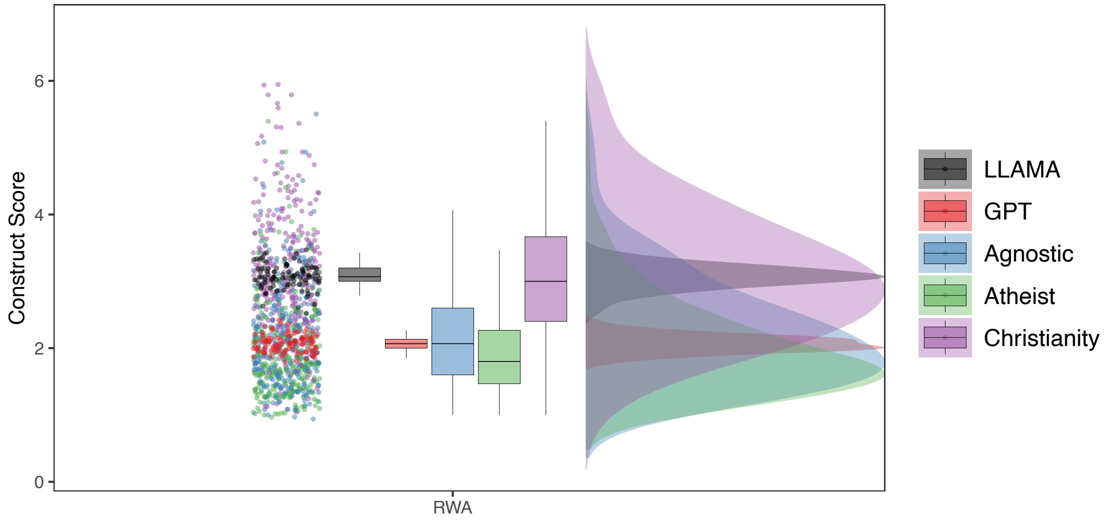

Figure S53

Comparing LLaMA and ChatGPT against Humans by Religion for responses on the Systemizing-Emphasizing Scale

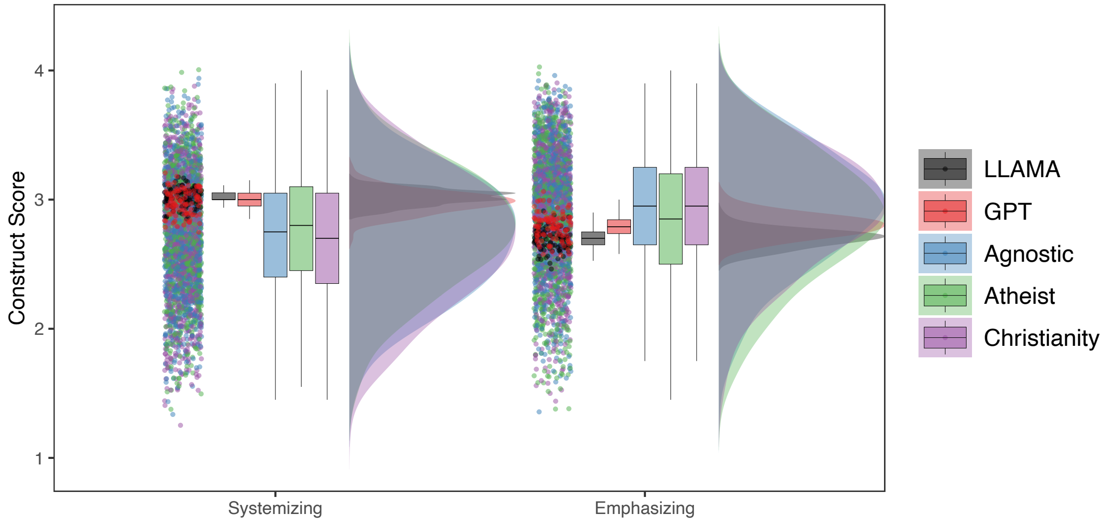

Table S51

Deviation of LLaMA responses from human responses grouped by political orientation

Survey	Construct	Demographic	*d*	*p*
BFI	Extraversion	Liberal	-0.29	.002
BFI	Agreeableness	Liberal	0.028	.926
BFI	Conscientiousness	Liberal	-0.11	.222
BFI	Neuroticism	Liberal	0.25	.008
BFI	Openness	Liberal	0.26	< .001
Need for Cognition	Need for Cognition	Liberal	0.67	< .001
Need for Closure	Need for Closure	Liberal	-1.40	< .001
Systemizing and Emphasizing	Systemizing	Liberal	-0.36	< .001
Systemizing and Emphasizing	Emphasizing	Liberal	0.30	< .001
Rational-Experiential Inventory	Rational	Liberal	0.76	< .001
Rational-Experiential Inventory	Experiential	Liberal	-0.04	.931
Right-Wing-Authoritarianism	Right-Wing-Authoritarianism	Liberal	-1.23	< .001
BFI	Extraversion	Moderate	-0.26	.005
BFI	Agreeableness	Moderate	-0.03	.939
BFI	Conscientiousness	Moderate	0.01	.998
BFI	Neuroticism	Moderate	0.03	.952
BFI	Openness	Moderate	0.13	.052
Need for Cognition	Need for Cognition	Moderate	0.56	< .001
Need for Closure	Need for Closure	Moderate	-	-
Systemizing and Emphasizing	Systemizing	Moderate	-0.30	< .001
Systemizing and Emphasizing	Emphasizing	Moderate	0.19	< .001
Rational-Experiential Inventory	Rational	Moderate	0.67	< .001
Rational-Experiential Inventory	Experiential	Moderate	0.000	1.000
Right-Wing-Authoritarianism	Right-Wing-Authoritarianism	Moderate	-0.48	< .001

Continued on next page

Table S51 – continued from previous page

Survey	Construct	Demographic	*d*	*p*
BFI	Extraversion	Conservative	-0.25	.016
BFI	Agreeableness	Conservative	-0.15	.067
BFI	Conscientiousness	Conservative	0.05	.806
BFI	Neuroticism	Conservative	-0.08	.577
BFI	Openness	Conservative	-0.000	1.000
Need for Cognition	Need for Cognition	Conservative	0.58	< .001
Need for Closure	Need for Closure	Conservative	-	-
Systemizing and Emphasizing	Systemizing	Conservative	-0.23	< .001
Systemizing and Emphasizing	Emphasizing	Conservative	0.08	.187
Rational-Experiential Inventory	Rational	Conservative	0.75	< .001
Rational-Experiential Inventory	Experiential	Conservative	0.03	.966
Right-Wing-Authoritarianism	Right-Wing-Authoritarianism	Conservative	0.62	< .001

Note: *Positive values indicate a higher average response for political groups compared to LLaMA. Significance determined via Dunnett's Test.*

Figure S54

Comparing LLaMA and ChatGPT against Humans by Political Orientation for responses on the BFI

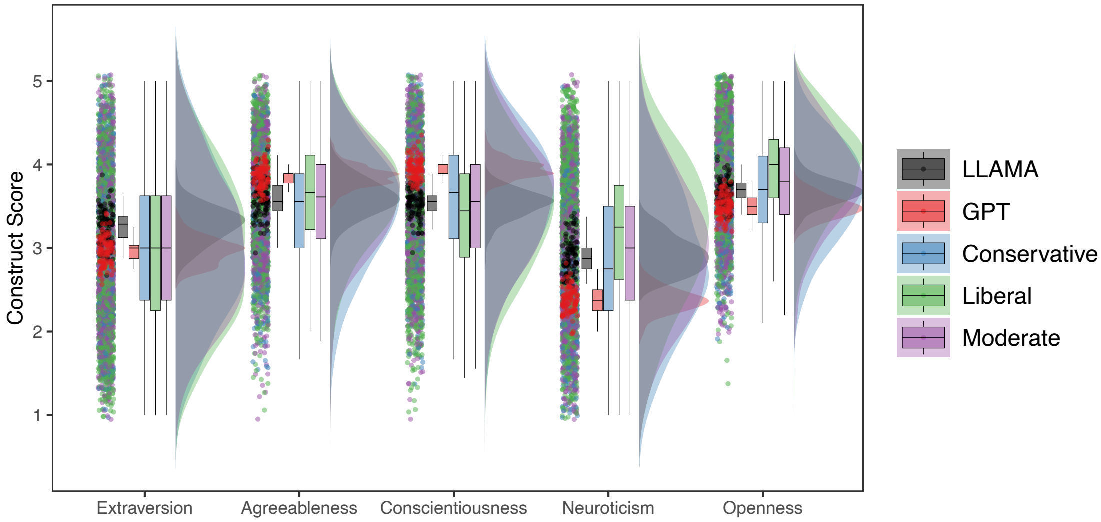

Figure S55

Comparing LLaMA and ChatGPT against Humans by Political Orientation for responses on the Need for Cognition questionnaire

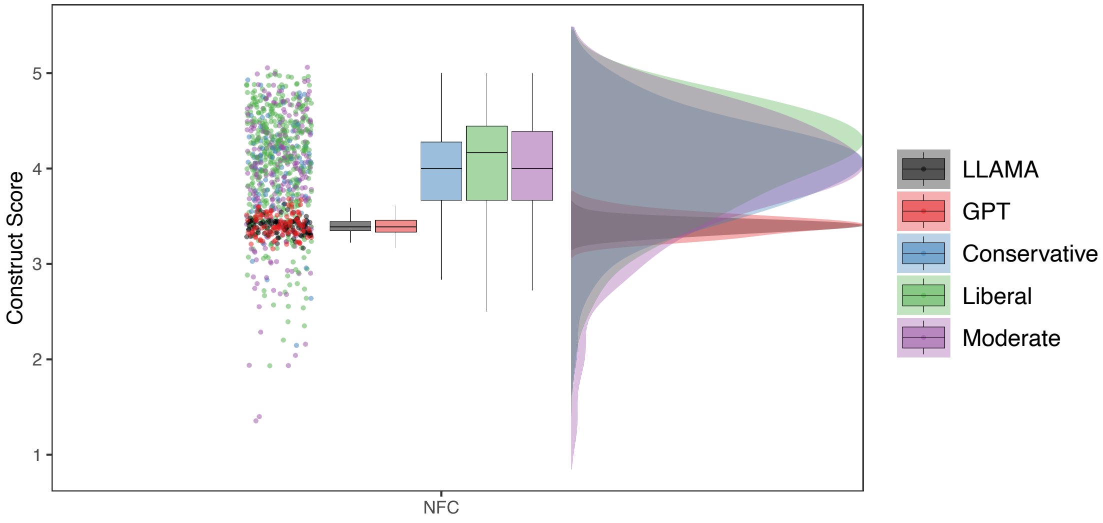

Figure S56

Comparing LLaMA and ChatGPT against Humans by Political Orienation for responses on the Need for Closure Survey

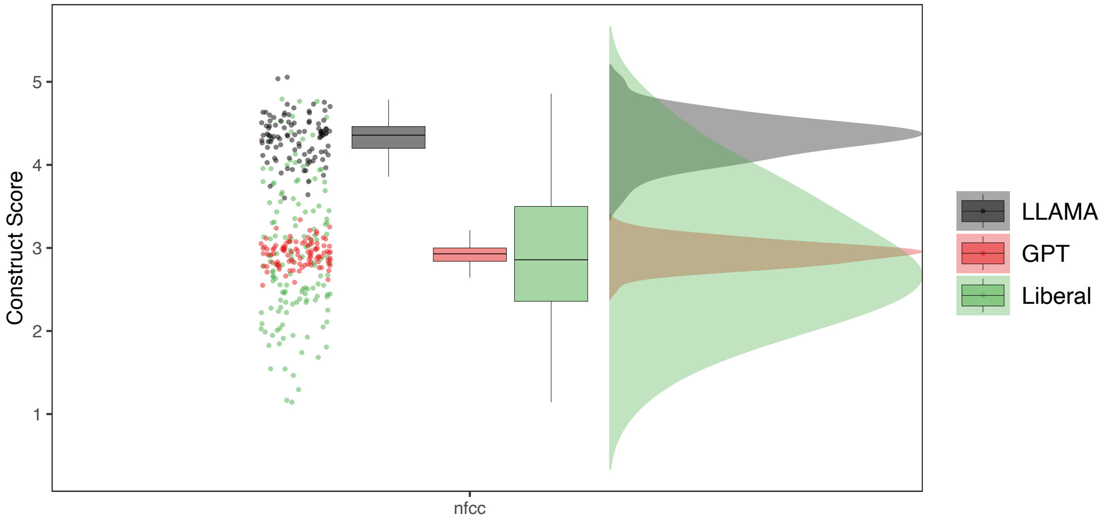

Figure S57

Comparing LLaMA and ChatGPT against Humans by Political Orientation for responses on the Rational-Experiential Inventory

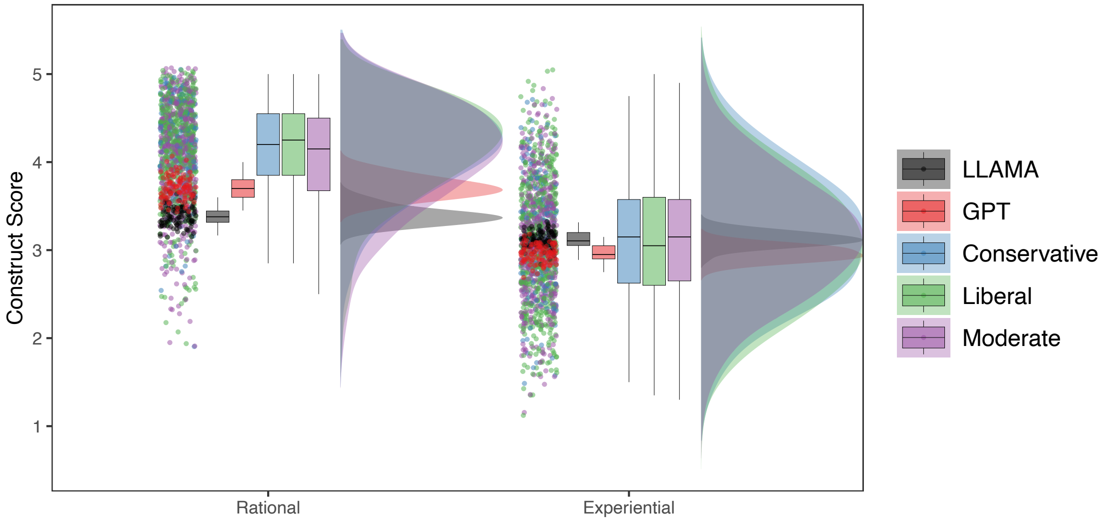

Figure S58

Comparing LLaMA and ChatGPT against Humans by Political Orientaition for responses on the Right-Wing-Authoritarianism Scale

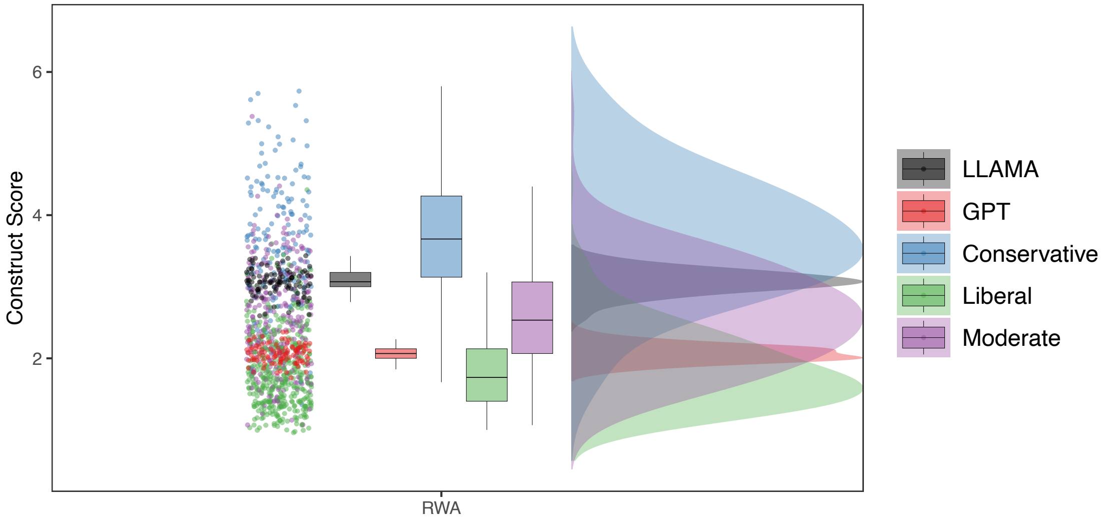

Figure S59

Comparing LLaMA and ChatGPT against Humans by Political Orientation for responses on the Systemizing-Emphasizing Scale

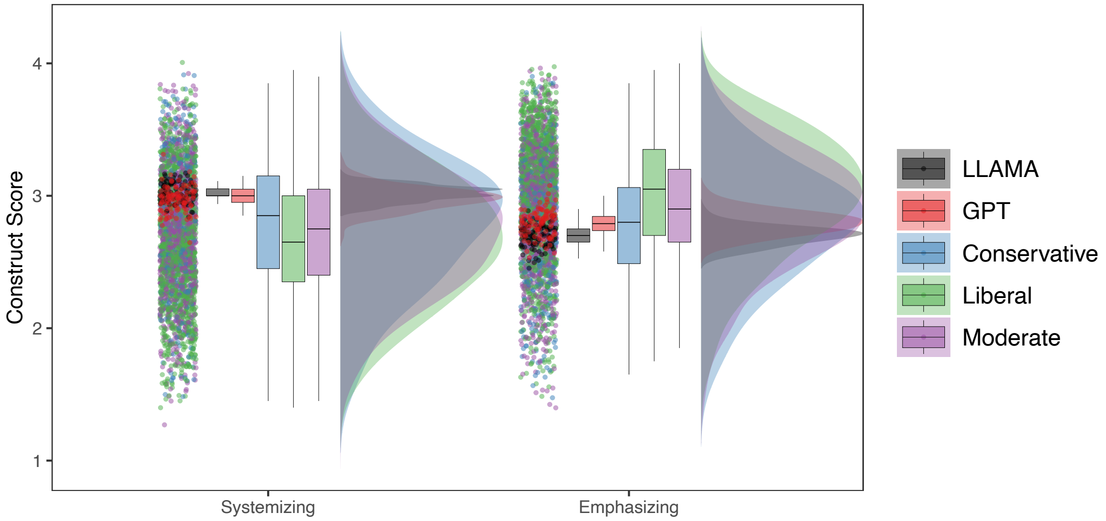

General Summary

In total, we showed that by using a simple pipeline that fully runs on consumer-grade hardware, researchers can conduct LLM analyses with open LLMs. During moral sentiment analysis, our LLaMA model achieved marginally higher accuracies and misclassified some moral sentiments significantly less frequently compared with ChatGPT. For survey responses, we found comparable output quality with similar response distributions. In some cases, LLaMA achieved notably more natural response patterns and less bias against human demographics (especially on personality). Importantly, these results were achieved with a vastly smaller model. Several larger LLaMA alternatives that still run on single GPUs are available (e.g., 13B and 30B parameter versions) and thus improved performances are possible even on limited hardware.

This has relevant implications. Firstly, using open LLMs researchers can control what exact model they use, how it was trained, and on what data, increasing transparency. Additionally, the version applied by a researcher is permanent, meaning it cannot be altered by corporate decisions, policies, or general model updates. Thus, researchers can easily maintain the exact model version by simply uploading the model weights used in their study and thus facilitate reproducibility. Lastly, since the models were run locally instead of through a commercial API, no costs were associated with the model outputs. That is, we did not pay for the LLaMA text annotations and survey responses. In comparison, the ChatGPT model (3.5-turbo) costs $0.002/1000 tokens ($0.012-0.016/1000 tokens after fine-tuning) and ChatGPT4 costs between $0.03 to $0.12/1000 tokens, which can quickly scale up for large data sets or when collecting responses repeatedly to investigate response variances. However, it should be noted here that (non-fine-tuned) ChatGPT3.5 is, currently, quite affordable. In total, we spend around $100 dollars for the different analyses (in total approximately 90,000 data points). On the other hand, fine-tuning currently significantly increases costs. We spend as much on a single fine-tuning task as for all other tasks combined ($\approx$ $100). Additionally, the cost depends on the number of tokens and thus the length of analyzed and generated text. In our analyses, we focused on short questionnaire items or short social media texts and generated very short outputs in the form of single-digit or word ratings. Since ChatGPT takes the length of both instructions and generated outputs into account, affordability strongly depends on both the model quality (e.g., GPT4 being

15-100 times more expensive than GPT3.5) and data size (total length of all inputs). Researchers should thus consider if the long-term costs for large-scale projects using high-quality models (e.g., social media studies using millions of messages) are justified. Beyond financial costs, there are also environmental costs to consider. Training language models is energy intensive. For example, training a BERT model from scratch requires as much energy as a trans-American flight and can therefore cause significant carbon emissions (Bender et al., 2021; Strubell et al., 2019b). These issues are dramatically more severe for the newest generation of LLMs which have undergone an explosion in model size, which accompanied by ever increasing energy demands for both training the model and deploying it (Bender et al., 2021). A push towards more efficiency, instead of only more performance, is therefore urgently needed (Bender et al., 2021; R. Schwartz et al., 2020). Recently, there have been discussions on whether LLMs are getting too big, reconsidering efficiency over performance, and highlighting environmental concerns regarding NLP applications in general (e.g., Bender et al., 2021; R. Schwartz et al., 2020; Strubell et al., 2019a, 2019b) but, for now, improving performance is the dominant perspective in both industry and research (R. Schwartz et al., 2020).

Supplementary Materials' References

Abadi, M., Barham, P., Chen, J., Chen, Z., Davis, A., Dean, J., Devin, M., Ghemawat, S., Irving, G., Isard, M., et al. (2016). Tensorflow: A system for large-scale machine learning. *12th {USENIX} Symposium on Operating Systems Design and Implementation ({OSDI} 16)*, 265–283.

Abdel-Khalek, A. M. (2007). Assessment of intrinsic religiosity with a single-item measure in a sample of arab muslims. *Journal of Muslim Mental Health*, *2*(2), 211–215.

Afhami, R., Mohammadi-Zarghan, S., & Atari, M. (2017). Self-rating of religiosity (srr) in iran: Validity, reliability, and associations with the big five. *Mental Health, Religion & Culture*, *20*(9), 879–887.

Andri et mult. al., S. (2021). *DescTools: Tools for descriptive statistics* [R package version 0.99.44]. https://cran.r-project.org/package=DescTools

Anonymous. (2023). Text-generation-webui.

Atari, M., Haidt, J., Graham, J., Koleva, S., Stevens, S. T., & Dehghani, M. (2023). Morality beyond the weird: How the nomological network of morality varies across cultures. *Journal of Personality and Social Psychology.*

Atari, M., Omrani, A., & Dehghani, M. (2023). Contextualized construct representation: Leveraging psychometric scales to advance theory-driven text analysis. https://psyarxiv.com/m93pd

Baron-Cohen, S., Richler, J., Bisarya, D., Gurunathan, N., & Wheelwright, S. (2003). The systemizing quotient: An investigation of adults with asperger syndrome or high–functioning autism, and normal sex differences. *Philosophical Transactions of the Royal Society of London. Series B: Biological Sciences*, *358*(1430), 361–374.

Bender, E. M., Gebru, T., McMillan-Major, A., & Shmitchell, S. (2021). On the dangers of stochastic parrots: Can language models be too big? *Proceedings of the 2021 ACM conference on fairness, accountability, and transparency*, 610–623.

Boyd, R. L., & Schwartz, H. A. (2021). Natural language analysis and the psychology of verbal behavior: The past, present, and future states of the field. *Journal of Language and Social Psychology*, *40*(1), 21–41.

Cacioppo, J. T., & Petty, R. E. (1982). The need for cognition. *Journal of personality and social psychology*, *42*(1), 116.

Chiang, W.-L., Li, Z., Lin, Z., Sheng, Y., Wu, Z., Zhang, H., Zheng, L., Zhuang, S., Zhuang, Y., Gonzalez, J. E., Stoica, I., & Xing, E. P. (2023, March). Vicuna: An open-source chatbot impressing gpt-4 with 90%* chatgpt quality. https://lmsys.org/blog/2023-03-30-vicuna/

Chollet, F., et al. (2015). *Keras*. https://github.com/fchollet/keras

Cortes, C., & Vapnik, V. (1995). Support-vector networks. *Machine learning*, *20*(3), 273–297.

Dettmers, T., & Zettlemoyer, L. (2023). The case for 4-bit precision: K-bit inference scaling laws.

Devlin, J., Chang, M.-W., Lee, K., & Toutanova, K. (2018). Bert: Pre-training of deep bidirectional transformers for language understanding. https://arxiv.org/abs/1810.04805

Dunnett, C. W. (1955). A multiple comparison procedure for comparing several treatments with a control. *Journal of the American Statistical Association*, *50*(272), 1096–1121.

Fossati, A., Borroni, S., Marchione, D., & Maffei, C. (2011). The big five inventory (bfi). *European Journal of Psychological Assessment*.

Fox, J., & Weisberg, S. (2019). *An R companion to applied regression* (Third). Sage. https://socialsciences.mcmaster.ca/jfox/Books/Companion/

Garten, J., Hoover, J., Johnson, K. M., Boghrati, R., Iskiwitch, C., & Dehghani, M. (2018). Dictionaries and distributions: Combining expert knowledge and large scale textual data content analysis: Distributed dictionary representation. *Behavior research methods*, *50*, 344–361.

Gelfand, M. J., Raver, J. L., Nishii, L., Leslie, L. M., Lun, J., Lim, B. C., Duan, L., Almaliach, A., Ang, S., Arnadottir, J., et al. (2011). Differences between tight and loose cultures: A 33-nation study. *science*, *332*(6033), 1100–1104.

Goutte, C., & Gaussier, E. (2005). A probabilistic interpretation of precision, recall and f-score, with implication for evaluation. *European conference on information retrieval*, 345–359.

Levene, H. (1960). Robust tests for equality of variances. *Contributions to probability and statistics*, 278–292.

OpenAI. (2023, June 6). *Openai python package* (Version 0.27.8). https://github.com/openai/openai-python

Oyserman, D. (1993). The lens of personhood: Viewing the self and others in a multicultural society. *Journal of personality and social psychology*, *65*(5), 993.

Pacini, R., & Epstein, S. (1999). The relation of rational and experiential information processing styles to personality, basic beliefs, and the ratio-bias phenomenon. *Journal of personality and social psychology*, *76*(6), 972.

Pedregosa, F., Varoquaux, G., Gramfort, A., Michel, V., Thirion, B., Grisel, O., Blondel, M., Prettenhofer, P., Weiss, R., Dubourg, V., et al. (2011). Scikit-learn: Machine learning in python. *Journal of machine learning research*, *12*(Oct), 2825–2830.

Pennebaker, J. W., Boyd, R. L., Jordan, K., & Blackburn, K. (2015). *The development and psychometric properties of liwc2015* (tech. rep.).

R Core Team. (2021). *R: A language and environment for statistical computing.* R Foundation for Statistical Computing. Vienna, Austria. https://www.R-project.org/

Schwartz, R., Dodge, J., Smith, N. A., & Etzioni, O. (2020). Green ai. *Communications of the ACM*, *63*(12), 54–63.

Schwartz, S. H. (2003). A proposal for measuring value orientations across nations. *Questionnaire package of the european social survey*, *259*(290), 261.

Strubell, E., Ganesh, A., & McCallum, A. (2019a, July). Energy and policy considerations for deep learning in NLP. In A. Korhonen, D. Traum, & L. Màrquez (Eds.), *Proceedings of the 57th annual meeting of the association for computational linguistics* (pp. 3645–3650). Association for Computational Linguistics. https://doi.org/10.18653/v1/P19-1355

Strubell, E., Ganesh, A., & McCallum, A. (2019b). Energy and policy considerations for deep learning in nlp. *arXiv preprint arXiv:1906.02243*.

Taori, R., Gulrajani, I., Zhang, T., Dubois, Y., Li, X., Guestrin, C., Liang, P., & Hashimoto, T. B. (2023). Stanford alpaca: An instruction-following llama model.

Trager, J., Ziabari, A. S., Davani, A. M., Golazazian, P., Karimi-Malekabadi, F., Omrani, A., Li, Z., Kennedy, B., Reimer, N. K., Reyes, M., et al. (2022). The moral foundations reddit corpus. https://arxiv.org/abs/2208.05545

Turc, I., Chang, M.-W., Lee, K., & Toutanova, K. (2019). Well-read students learn better: On the importance of pre-training compact models. https://arxiv.org/abs/1908.08962v2

Van Rossum, G., & Drake, F. L. (2009). *Python 3 reference manual*. CreateSpace.

Webster, D. M., & Kruglanski, A. W. (1994). Need for closure scale. *Journal of Personality and Social Psychology*.

Zakrisson, I. (2005). Construction of a short version of the right-wing authoritarianism (rwa) scale. *Personality and individual differences*, *39*(5), 863–872.
